# A genome-scale drug discovery pipeline uncovers therapeutic targets and a unique p97 allosteric binding site in *Schistosoma mansoni*

**DOI:** 10.1073/pnas.2505710122

**Published:** 2025-08-29

**Authors:** Dylon R. Stephens, Ho Yee Joyce Fung, Yan Han, Jue Liang, Zhe Chen, Joseph Ready, James J. Collins

**Affiliations:** ^a^Department of Pharmacology, University of Texas Southwestern Medical Center, Dallas, TX 75390; ^b^Department of Biophysics, University of Texas Southwestern Medical Center, Dallas, TX 75390; ^c^Department of Biochemistry, University of Texas Southwestern Medical Center, Dallas, TX 75390; ^d^HHMI, Chevy Chase, MD 20815

**Keywords:** *Schistosome*, parasitology, p97, drug discovery

## Abstract

Schistosomes are parasitic flatworms that infect more than 200 million people globally. However, there is a shortage of molecular tools that enable the discovery of potential drug targets within schistosomes. Thus, praziquantel has remained the frontline treatment for schistosomiasis despite known liabilities. Here, we have conducted a genome-wide study in *Schistosoma mansoni* using the human druggable genome as a bioinformatic template to identify essential genes within schistosomes bearing similarity to catalogued drug targets. Then, we assessed these candidate targets in silico using a set of unbiased criteria to determine which possess ideal characteristics for a ready-made drug discovery campaign. Following this prioritization, we pursued a parasite p97 ortholog as a bona-fide drug target for the development of therapeutics to treat schistosomiasis. From this effort, we identified a covalent inhibitor series that kills schistosomes through an on-target killing mechanism by disrupting the ubiquitin proteasome system. Fascinatingly, these inhibitors induce a conformational change in the conserved D2 domain P-loop of schistosome p97 upon modification of Cys519. This conformational change reveals an allosteric binding site adjacent to the D2 domain active site reminiscent of the “DFG” flip in protein kinases. This allosteric binding site can potentially be utilized to generate new classes of species-selective p97 inhibitors. Furthermore, these studies provide a resource for the development of alternative therapeutics for schistosomiasis and a workflow to identify potential drug targets in similar systems with few available molecular tools.

## Significance

Schistosomes cause widespread infections in humans, leading to severe chronic illnesses in endemic regions. There is no vaccine for schistosomiasis, and there has been limited success using the current standard-of-care treatment, praziquantel. Therefore, it is essential to identify drug targets within these parasites. Here, we identify potential drug targets in schistosomes bearing similarity to established human therapeutic targets, evaluate their essentiality for parasite survival, then prioritize them using an unbiased set of criteria to uncover high-value targets for the treatment of schistosomiasis. We investigated one candidate as a proof-of-concept, a p97 ortholog, to discover newly characterized inhibitors of the parasite enzyme. This study demonstrates that this workflow can lead to the identification of small molecules that kill schistosomes.

Schistosomiasis affects more than 200 million of the world’s poorest people (1), claiming the lives of ~250,000 people yearly (2), while placing a significant clinical and economic burden on endemic regions (1). Among the most practical challenges facing eradication of schistosomiasis is that there is no vaccine, and treatment has relied on a single drug, praziquantel (PZQ), for over 40 y.

Despite its exclusive use in the treatment of schistosomiasis since the 1970s (3–5), PZQ possesses prominent and persistent pharmaceutical and pharmacological liabilities. For instance, cure rates following PZQ treatment vary dramatically in regions with high infection rates (6–8). Because of this, there are valid concerns that mechanisms of PZQ resistance will become widespread. Indeed, reduced sensitivity can be induced rapidly in a laboratory setting (9, 10), and loss of function mutations in a PZQ-sensitive ion channel have already been identified in the field (11). As PZQ has proven unable to lead to the elimination of schistosomiasis in endemic regions on its own (12), serious dedicated efforts are required to identify drug targets for the development of alternative therapeutics to PZQ.

However, identification of novel schistosomicidals has proven difficult. The relatively large size of adult worms (~1 cm) prevent them from being adapted to micronized volumes amenable to high-throughput phenotypic screens, and molecules derived from screening against larval parasites suffer from high rates of attrition when examined in adult worms (13–15). Because of these limitations in identifying chemicals that can kill the adult worms that drive disease pathology, an attractive alternative is the discovery of lead compounds through target-based approaches. As yet, there are few validated drug targets within schistosomes (16, 17). Even with progress in the development of large-scale RNAi approaches (18), a paucity of facile molecular tools has hindered the systematic identification and subsequent functional validation of target proteins in the worm. Deciphering the function of essential genes still requires a large investment in labor and time. And, while there is an obvious requirement that drug targets be essential, essentiality alone is not sufficient to anticipate which targets will lead to successful drug discovery efforts. Many target-based screening efforts failed because the selected targets were unable to bind drug-like molecules with reasonable affinity (19, 20).

This paper expands upon our previous work by 1) identifying essential genes within the *Schistosoma mansoni* genome that bear homology to well-defined drug targets in the context of human disease using bioinformatic and genetic approaches and 2) assessing these genetically essential targets for viability as “bona fide” drug targets utilizing well-defined criteria. Combining our findings from RNAi experiments in this study with those performed previously (18), we have prioritized 18 potential drug targets using an unbiased set of criteria. We investigated one of these targets in-depth, p97, a gene encoding a AAA-ATPase involved in the degradation of proteins via the ubiquitin proteasome system (UPS) (21). Following high-throughput screening efforts, we identified a benzoxazole propiolamide covalent scaffold that inhibits the enzyme, exhibits selectivity for the parasite ortholog over its human counterpart, and displays on-target effects in adult parasites via accumulation of K48 polyubiquitinated proteins (18, 22). Furthermore, we obtained a structure of p97 bound to these covalent inhibitors using cryo-EM, identifying a previously unreported conformational change in the nucleotide (Walker A) binding motif of p97 within its D2 domain (23). This conformational change creates a novel allosteric pocket between p97 monomers that may enable species-selective inhibitors to be developed. Together, these studies highlight a validated set of high-value targets that will serve as the foundation for future drug discovery campaigns.

## Results

### Large-Scale RNAi Screen Reveals 63 Genes Essential for In Vitro Parasite Survival.

**Fig. 1.**
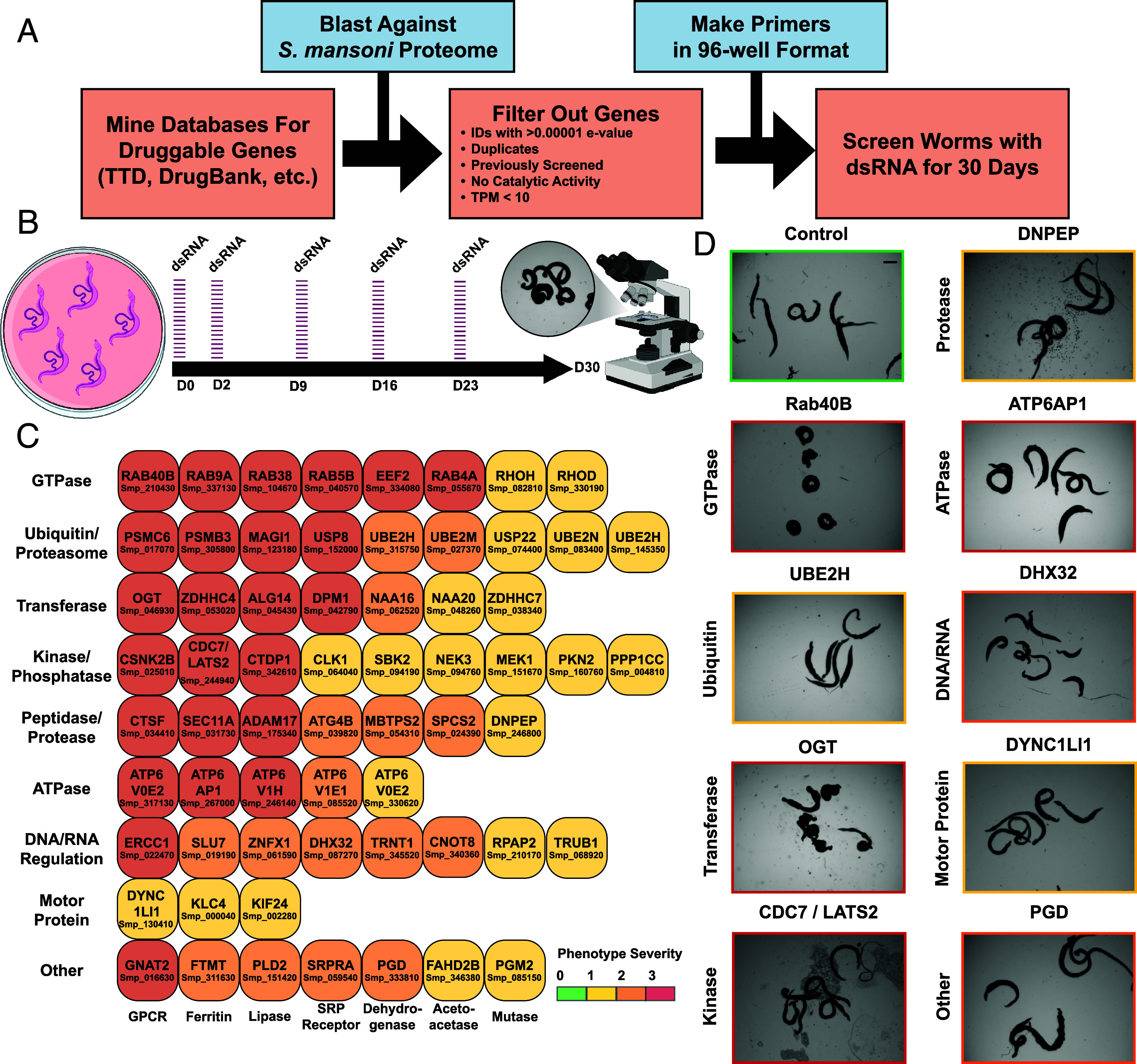
Large-scale RNAi screen targeting [Human] druggable genes. (*A*) Bioinformatic pipeline for the identification of potentially druggable genes. Target genes were identified using databases such as TTD, DrugBank, ChEMBL, etc., BLAST-p was used to identify similar proteins in the *S. mansoni* proteome, then passed through several preliminary qualifications before being included in the screen. Primers possessing a T7 promoter in addition to a 500 to 1,000 bp target gene region were created to generate dsRNA for final candidate genes in 96-well format before treatment. (*B*) RNAi treatment schedule. Parasites were treated with dsRNA on D0 and D2, then every 7 d thereafter (D9, 16, and 23). Gross morphology was monitored over the course of 30 d by light microscopy. Observations on substrate attachment, movement, and morphology were taken every 1 to 2 d coinciding with media changes. Images were taken on Day 30 to assess final phenotypes. (*C*) Categorical arrangement of essential genes producing phenotypes according to enzymatic activity/biological function. Severity of phenotypes that appeared were scored based on how early the phenotype began to appear (D7 to D15, D16 to D25, and post-D25), as well as the number of visual phenotypes that manifested (tissue/gut edema, tegument/head degeneration, hypercontraction, death, etc.). Scoring follows description for RNAi Severity in *SI Appendix*, Table S1. Red (3) represents a gene who presented more than one physical phenotype and appeared between D1 and D15. Orange (2) represents a gene that presented more than one phenotype between D16-D25. Yellow (1) denotes a gene with an intermediate onset (D16-D25) of a modest phenotype. Green (0) represents a modest phenotype that appears after D25. (*D*) Representative images of phenotypes based on enzyme category compared to control dsRNA treatment (pJC53.2). GTPase RAB40B (Smp_210430), Ubiquitin transferase UBE2H (Smp_145350), Protein Kinase CDC7/LATS2 (Smp_244940), Glycosyltransferase OGT (Smp_046930), RNA helicase DHX32 (Smp_130410), V-type proton ATPase subunit ATP6AP1 (Smp_267000), Aminopeptidase DNPEP (Smp_246800), Dynein-light chain DYNC1LI1 (Smp_130410), and 6-Phosphogluconate dehydrogenase PGD (Smp_333810). [Scale bar (*D*), 1,000 μm.]

With the success of previous RNAi studies (18), we revisited the *S. mansoni* genome to determine other potentially attractive targets that remain uncharacterized. To improve our chances of success in finding essential genes, as well as potential drug targets, we utilized known “druggable” targets catalogued in databases such as DrugBank (24), ChEMBL (25), and TTD (26) (Fig. 1*A* and Dataset S1).

After removing genes already characterized by RNAi, we included additional stringencies to generate a list of high confidence genes likely to be essential. We found that the majority (~57%) of “hits” from our initial RNAi study encoded either enzymes or large catalytic complexes such as the proteasome (18). Because enzymes are likely to provide the best rate-of-return, we narrowed our list of genes to those predicted to encode proteins with catalytic activity based on gene ontology terms (27, 28) (Dataset S1). We also refined this list for genes with an expression greater than 10 transcripts per million (TPM) (Dataset S1). Retrospective analysis from Wang et al. found such genes are more likely to result in visible RNAi phenotypes than those with a lower expression level (<10 TPM). This filtering step enabled us to identify 576 genes with significant amino acid similarity (BLAST e-value < 1e-20) to catalogued druggable targets (Dataset S1) (29–31).

Out of the initial 576 genes, we were able to amplify and generate sufficient double-stranded RNAs (dsRNAs) for 507 genes (88%) (Dataset S1). To evaluate RNAi knockdown phenotypes for these genes of interest, we treated adult male and female pairs with dsRNA over the course of 30 d (Fig. 1 *A* and *B*). During these experiments (Fig. 1*B*), we monitored worm health under in vitro culture conditions. Healthy parasites attach to tissue culture substrate using both their oral and ventral suckers and can move inside of the culture vessel. Substrate attachment and normal movement were used as a preliminary readout of worm viability, identifying other previously defined visible defects by light microscopy as they arose (tissue/gut edema, tegument/head degeneration, hypercontraction, death, etc.) (18).

Genes producing phenotypes following RNAi treatment were validated by confirming gene identity using DNA sequencing and knockdown specificity through designing additional dsRNAs targeting a nonoverlapping gene region when possible (Dataset S1). These studies yielded 63 genes (~11% hit rate) which produced fully penetrant phenotypes affecting attachment upon knockdown. Many worms displayed phenotypes in addition to detachment defects following depletion of individual genes of interest (*SI Appendix*, Fig. S1 and Dataset S1).

### Focused RNAi Screen Yields Potential Targets that Can be Grouped According to Enzymatic Function.

Of the 63 genes with demonstrated fitness cost upon knockdown, we found that many fell into broad categories of enzyme activity (Fig. 1 *C* and *D* and Dataset S2). We identified essential genes with predicted functions in the categories of GTPases, ubiquitination/proteasomal degradation, transferases (acetyl, palmitoyl, glycosyl, etc.), kinases and phosphatases, peptidases/proteases, ATPases, DNA/RNA regulation, and motor proteins (Fig. 1 *C* and *D*). We also found additional genes that were predicted to perform functions in other pathways that did not fall into any of these categories (GPCR, mutase, dehydrogenase, lipase, etc.). These data reaffirm many of the trends that we observed in our previous work (18). Particularly, that parasites are especially sensitive to knockdown of genes encoding proteins involved in proteostasis and RNAi targeting large protein complexes like the proteasome are likely to produce a phenotype. The V-type (vacuolar) ATP-dependent proton channel exemplifies another essential large protein complex represented in our dataset, the knockdown of numerous individual subunits producing phenotypes following RNAi.

Two of the most abundant categories that produced severe phenotypes were GTPases and kinases, the former commonly leading to rapid death in adult parasites upon knockdown (*SI Appendix*, Fig. S1). GTPases present as molecular switches for numerous key cellular processes (32, 33), and kinases are frequently involved in vital signaling cascades (34, 35). Thus, it is understandable that depletion of these genes would result in deleterious effects.

In addition, there were several proteases and peptidases whose knockdown produced phenotypes in parasites. Peptidases have often been proposed as potential drug targets for the development of therapeutics for schistosomiasis because of their involvement in critical cellular processes (36), such as the proteolytic invasion machinery of cercariae that enable them to infect their mammalian hosts (37, 38). Another example are the cathepsins (39, 40), a family of cysteine proteases (B1, L1/F, L2, L3, C, and D) that are secreted into the schistosome gut and aid in the digestion of hemoglobin and its subsequent metabolism. Confirming the necessity of these enzymes for normal parasite function, we identified a putative cathepsin F ortholog (Smp_034410) that was essential for parasite survival in vitro (Dataset S1 and *SI Appendix*, Fig. S1). We also identified proteases that function as key regulators in cell signaling and developmental programs (41). A putative ADAM17 ortholog was found to be essential for parasite survival (Dataset S1 and *SI Appendix*, Fig. S1). This metalloenzyme is expressed ubiquitously in mammals and contributes to important physiological processes (42), such as the processing of tumor necrosis factor (TNF)-α, a critical factor in inflammation (43, 44).

### In Silico Prioritization of Potential Drug Targets.

**Fig. 2.**
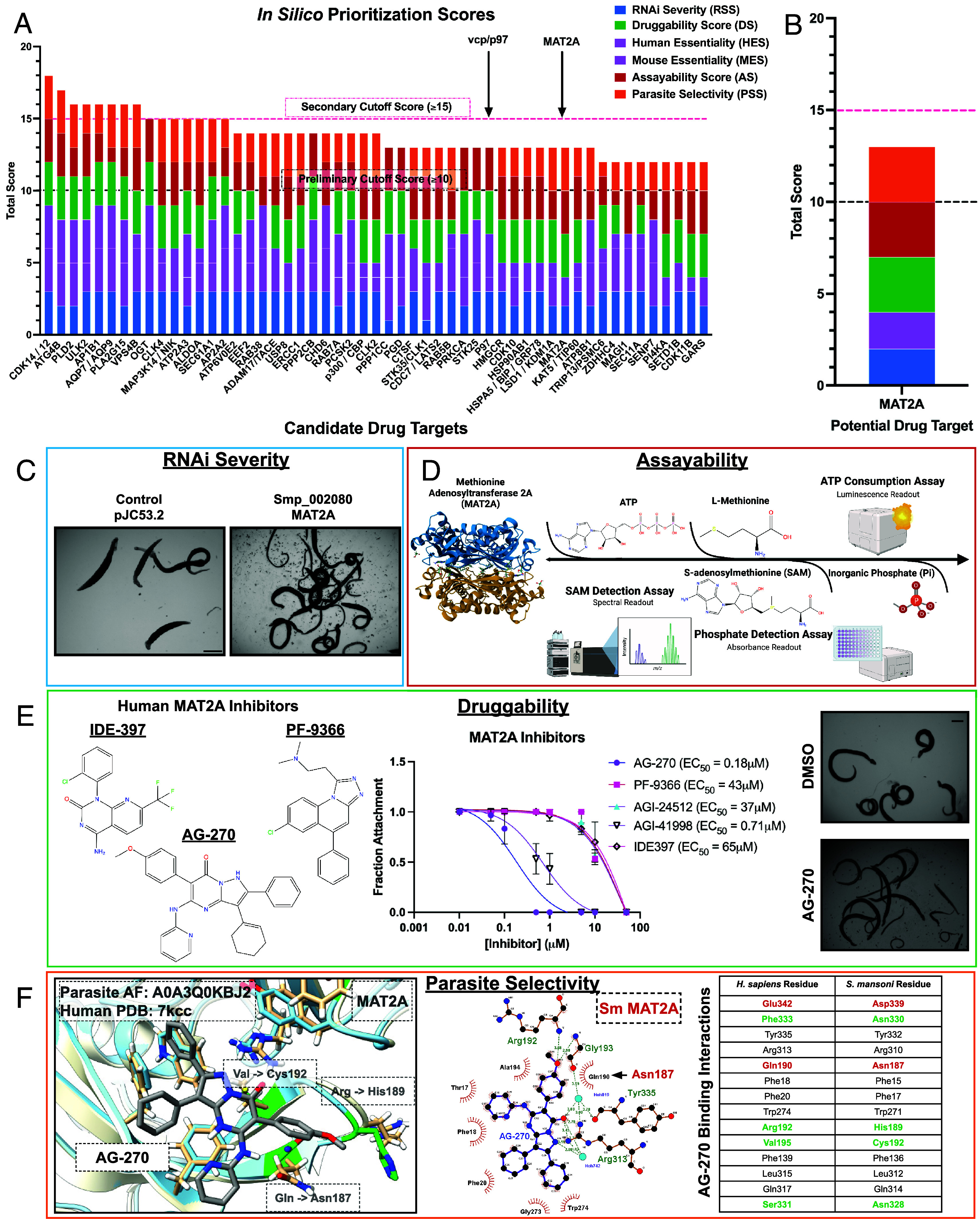
Prioritization scoring of essential schistosome genes reveals potential therapeutic targets. (*A*) In silico prioritization of essential genes identified by RNAi experiments. Representative targets shown were the top 53 highest scoring candidates. Potential targets were listed and prioritized according to scoring criteria detailed in *SI Appendix*, Table S1. Those identifiers that possessed a score ≥10 in initial scoring were scored according to the criteria set in the parasite selectivity section in addition. Thresholds were set at 10 to prioritize targets with ideal characteristics in three or more categories, and ≥15 to represent candidates that scored well in each category. (*B*) Example of scoring for *S. mansoni* MAT2A (SmMAT2A; Smp_002080). (*C*–*F*) Visual representation of experimental data included in prioritization criteria. (*C*) RNAi severity score (RSS). Light microscopy images of adult worms treated with control dsRNA (pJC53.2) and dsRNA targeting SmMAT2A. (*D*) Assayability score (AS). Outline of various techniques used to measure the biochemical activity of SmMAT2A (SAM Detection—RapidFire MS, ATP Consumption—Kinase Glo, Inorganic Phosphate Detection—PiColorLock). (*E*) Druggability score (DS). Structures of known *Homo sapiens* MAT2A (HsMAT2A) allosteric inhibitors AG-270, PF-9366, and IDE-397. Light microscopy images of adult worms treated with DMSO control or HsMAT2A inhibitor AG-270 at 10 μM. Dose–response curve of adult parasites treated with different HsMAT2A inhibitors (AG-270, PF-9366, IDE397, AGI-41998, and AGI-24512). Compounds were tested from a range of 50 μM to 10 nM to determine EC_50_. Values were determined by Prism. (*F*) Parasite selectivity score (PSS). Alphafold structure of SmMAT2A (Blue; AF: A0A3Q0KBJ2) aligned with HsMAT2A (White; PDB: 7KCC) bound to a known inhibitor, AG-270. Unique schistosome residues are outlined in green. LigPlot schematic showing HsMAT2A (PDB: 7KCC) bound to AG-270. Table depicting residues predicted to be involved in binding to AG-270. Conserved amino acid residues involved in binding are labeled black, while residues that are significantly different between the schistosome and human protein are colored green. Minor residue changes, such as small changes in size, are colored red. [Scale bar (*C* and *E*), 1,000 μm.]

Our previous study identified 181 genes producing a phenotype upon knockdown (18), and the experiments performed here revealed an additional 63 (Fig. 1 *C* and *D* and Dataset S1 and *SI Appendix*, Fig. S1). However, not all essential genes are equally targetable with drugs. Thus, it is unlikely the 244 potential targets from our RNAi studies are equally suited for the development of therapeutics. Our goal is not only to identify essential genes encoding potential drug targets, but also to provide data that enable us to decide which targets are best to proceed with for a ready-made drug discovery campaign. Therefore, we have decided upon a set of unbiased criteria that represents characteristics for an ideal drug target, thereby enabling us to distinguish among potential candidates. We selected prioritization criteria based on the rationale of identifying targets whose cellular processes are essential to the survival of parasites, provide an ample therapeutic window for the safe and effective treatment of disease, and have characteristics that are amenable to ready-made drug discovery campaigns. To enable us to prioritize targets for further in-depth experiments, we have assigned each target a numerical score based on the following criteria: RNAi phenotype severity, mammalian nonessentiality, assayability, druggability, and parasite selectivity (*SI Appendix*, Table S1 and Fig. 2*A*).

We reason that an ideal drug target is one whose knockdown leads to rapid and severe effects within the parasite, so the worms are completely debilitated when modulated by a drug and subsequently cleared by the host (RSS). To allow for a wide therapeutic window, we have chosen to prioritize targets whose homologs are nonessential in mammals (human and mice) (Essentiality Score). An ideal target should also encode a soluble protein that can be expressed and purified in a recombinant system in sufficient quantity and whose enzymatic activity can be measured reliably in high-throughput format using an easily adaptable, robust, commercially available assay (AS). Importantly, valuable drug targets need to bind drug-like molecules. Our prioritized drug targets should be closely related to human orthologs that possess commercially available small molecule modulators with drug-like properties (45–48) that show on-target activity in cellulo (DS). High-value targets should also have amino acid differences in critical binding regions, such as active sites, compared to their human counterpart to enable the development of species-selective drugs. This analysis was based on sequence and 3D structural alignments (PSS). One final consideration is that an ideal drug target should have a target-specific biological outcome that can be measured using well-established, robust methods to facilitate demonstration of on-target activity by a small molecule.

### Unbiased Categorization Reveals Several Attractive Targets for the Development of Therapeutics.

Using these metrics (*SI Appendix*, Table S1), we have analyzed a total of 244 genes from both our initial RNAi screen (18), as well as those uncovered in the present work (Fig. 2*A* and Dataset S3). Based on these criteria, we found that there were many potential drug targets that possessed ideal characteristics in most of these categories. Conversely, we found a clear cut-off at a cumulative score of 10 in prerequisite categories (RNAi severity, mammalian essentiality, assayability, and druggability) (Fig. 2*A*). Genes that fell below this threshold lacked ideal characteristics from two or more of these criteria. This does not mean that these are not valid drug targets, only that there are clear barriers in the path to drug discovery, whether that is a need for extensive purification or assay optimization, a lack of clear drug-like small molecule binding characteristics, or a limited therapeutic window.

Because of the intensive nature of structural studies, we utilized this cut-off score of ≥10 in the other composite categories to represent the targets with the most favorable properties for drug discovery. Using this cut-off, 85 potential targets qualified for additional investigation of selectivity using structural analysis (Dataset S3). However, as a number of these potential targets encoded individual subunits of singular complexes, we combined these targets into a single representative, reducing the final candidate pool to 65. Utilizing resources such as the Protein Data Bank (PDB) (49, 50), AlphaFold (51), and Clustal (52, 53), we have identified 51 potential drug targets that present favorable structural data (PSS ≥ 2) toward the development of selective drug-like molecules (Dataset S3), supplying structural and unique residue differences between the parasite and orthologous mammalian structures (*SI Appendix*, Fig. S3) that can be leveraged for drug development.

During our analysis, we uncovered numerous reported small-molecule compounds that act upon the predicted human orthologs of schistosome proteins of interest (Dataset S3 and *SI Appendix*, Table S4). Most of them produced a phenotype in the worms at an initial concentration of 10 μM (*SI Appendix*, Fig. S2 *A* and *B*), but there were interesting trends that these studies revealed to prioritize future targets of interest. First, there were inhibitors that killed parasites quickly (within 24 h), such as ULK-101, which targets ULK1/2 (*SI Appendix*, Fig. S2 *A* and *B*). The current standard of care, PZQ, paralyzes parasites within minutes following application. For a potential alternative therapy to compete with PZQ in a clinical setting, it is important that a drug act quickly, in a single dose. Therefore, drugs that display fast-killing kinetics on their cognate target are of great therapeutic interest (54). Second, there were targets whose inhibitors were potent at submicromolar concentrations (*SI Appendix*, Fig. S2*C*). Each of MEK1, ULK2, USP8, and MAT2A possessed commercial inhibitors that caused detachment of adult worms in culture in the nanomolar range (Fig. 2*E* and *SI Appendix*, Fig. S2*C*). It is sufficient to note that the most attractive targets for further drug discovery are those whose related compounds engage their target with high potency. Last, it was often the case that several classes of inhibitors were available for a given target. This enabled us to test different chemical scaffolds, or perhaps binding sites, for a single target and compare potencies on worms to what is known in literature. There were several targets, such as MEK1, where one known compound (Trametinib) was profoundly effective (EC_50_ = 650 nM), yet the other (Mirdametinib) was ineffective (EC_50_ > 50 μM) (*SI Appendix*, Fig. S2*C*). This suggests that there might be nontrivial differences in how these small molecules interact with their target in parasites compared to humans (e.g., permeability or target engagement). However, additional studies are needed to determine whether these compounds are engaging the predicted target by investigating potential downstream biological outcomes following inhibition of each of these targets (i.e., substrate phosphorylation, detection of metabolite abundance, etc.).

This workflow led to prioritization of 18 schistosome protein targets (Dataset S5) that we believe are the most promising to pursue in a drug discovery campaign. These final candidates represented ideal characteristics of our outlined criteria with the added benefit of a clear method to measure the biological outcome of pharmacologic modulation of the target by an inhibitor to determine on-target action. For instance, one hit, a schistosome ortholog of the methionine adenosyl transferase MAT2A (Smp_002080) embodied many of the ideal characteristics laid out in our prioritization criteria (Fig. 2*B*). MAT2A is an essential metabolic enzyme that catalyzes the synthesis of the primary methyl donor in cells, S-adenosylmethionine (SAM), from ATP and methionine (55). RNAi of the gene encoding schistosome MAT2A resulted in rapid detachment of parasites after roughly 2 wk of dsRNA treatment (Fig. 2*C*). Although MAT2A is essential in mice and various human cell lines, our own structural investigation revealed that this enzyme has an allosteric binding site that is targeted by many drugs and possesses residues distinct from its human counterpart (Fig. 2*F*) (56).

Allosteric inhibitors of MAT2A (Fig. 2*E*), such as PF-9366 (57), IDE-397 (58), and AG-270 (56), bind at the interface between two MAT2A monomers. There are integral residues that are conserved within this binding pocket, which likely explains how we still see activity on worms (Fig. 2*F*). But there are major changes within the surrounding pocket of AG-270, such as HsPhe333 to SmAsn330, HsArg192 to SmHis189, and HsSer331 to SmAsn328. In addition, a change from HsVal195 to SmCys192 creates an opportunity to target this allosteric binding site with covalent warheads, which would confer significant specificity to the schistosome protein over its human counterpart.

MAT2A is easily expressed in recombinant systems (*Escherichia coli*) in soluble fractions with high yield and it catalyzes a simple biochemical reaction to convert ATP and L-methionine into SAM (59). This reaction can be monitored using numerous robust methods that are amenable to high-throughput format, whether it is ATP consumption (Kinase-Glo), inorganic phosphate detection (PiColorLock), or SAM formation (RapidFire) (Fig. 2*D*). Last, the biological outcome of MAT2A inhibition within the context of the parasite can be easily measured through the detection of the common metabolite SAM using liquid chromatography followed by mass spectrometry (LC–MS). There is added credence to MAT2A as a bona fide drug target, as inhibitors of MAT2A are being investigated in Phase I clinical trials for tumors with loss of the gene MTAP, constituting 15% of cancers (56).

### p97 Is a Potential Drug Target for the Treatment of Schistosomiasis.

**Fig. 3.**
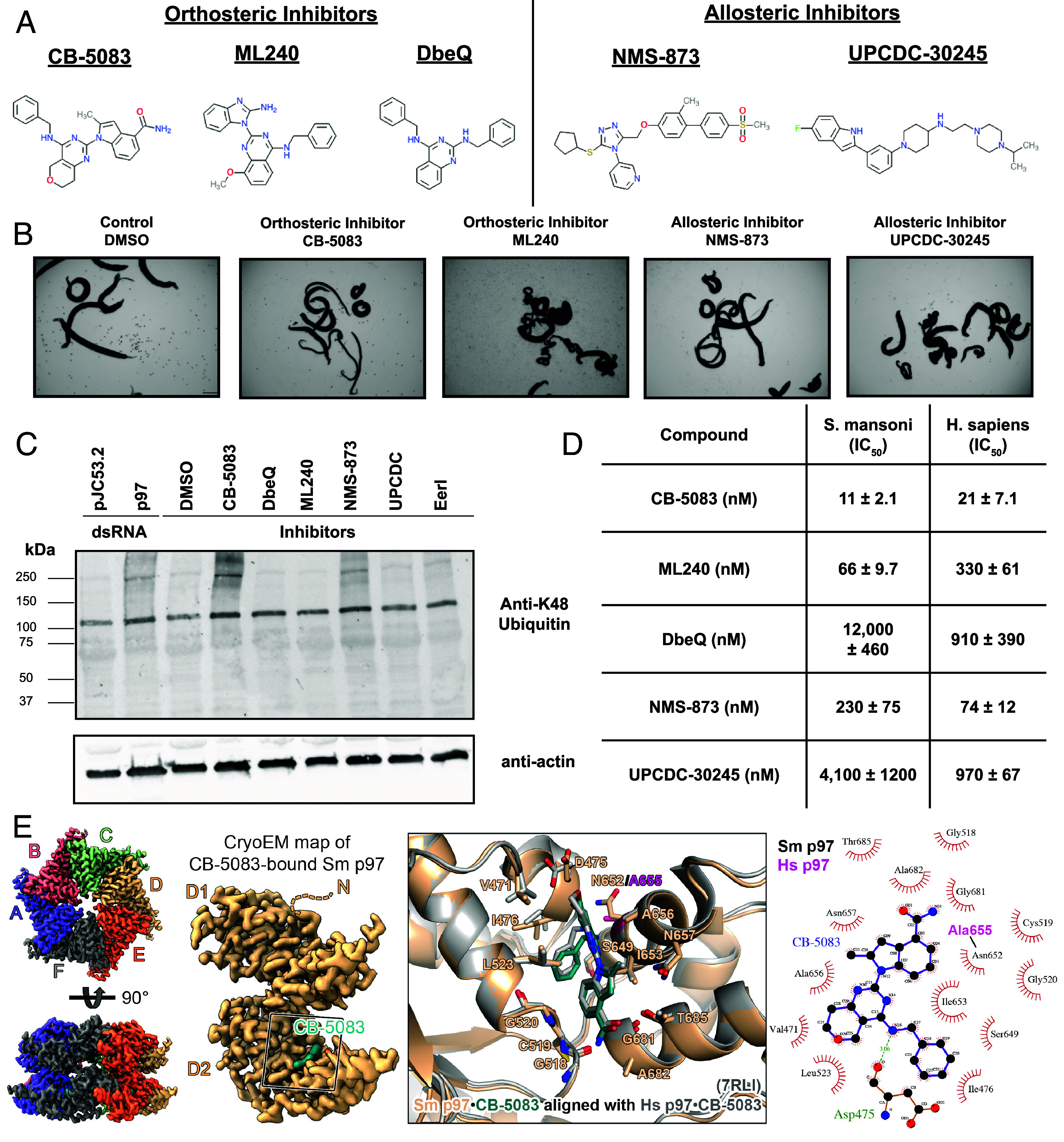
A parasite vcp/p97 homolog is essential for parasite survival and are affected by human inhibitors. (*A*) Chemical structures of established human p97 inhibitors classified as either active site (orthosteric) inhibitors (CB-5083, ML240, and DbeQ) or allosteric binding site inhibitors (NMS-873 or UPCDC-30245). (*B*) Light microscopy images of adult parasites treated with DMSO control or human p97 inhibitors at 10 μM. (*C*) Western blot depicting polyubiquitinated protein profile (K48 antibody) in worm lysate following treatment by dsRNA targeting p97 (pJC53.2 control) or established human p97 inhibitors (DMSO control). (*D*) Compared IC_50_ values of p97 inhibitors tested from 100 μM to 1 nM for *S. mansoni* and *H. sapiens* p97 recombinant enzyme using Kinase-Glo. Values were calculated in Prism. (*E*) Cryo-EM map and structure of the schistosome p97 hexamer in complex with the active site inhibitor CB-5083. Comparison of CB-5083 binding between the schistosome enzyme (Smp97; wheat) and human enzyme (Hsp97; gray, PDB: 7RLI). Ligplots were generated using LigPlot+ v2.2 using the structure of *S. mansoni* CB-5083-bound p97. Residues forming hydrophobic interactions are colored black, while other interactions are depicted in green. Unique human residues are colored pink. [Scale bar (*B*), 1,000 μm.]

As a proof-of-principle in the utility of our prioritization scheme, we conducted further studies with a schistosome protein encoding a putative valosin-containing protein/p97 (vcp/p97/cdc48) ortholog (Smp_018240). p97 is an abundant AAA-ATPase that serves a major part in the regulation of protein quality control, including mediating the degradation of ubiquitinated proteins via the endoplasmic reticulum associated degradation (ERAD) pathway (60, 61). Our lab previously described the rapid and severe phenotypes arising following p97 RNAi and the potent effects of inhibitors optimized for human p97 on adult parasites (18). Treatment with CB-5083 (Fig. 3 *A* and *B* and *SI Appendix*, Fig. S4*A*) (60, 62, 63), an ATP-competitive inhibitor, and NMS-873 (Fig. 3 *A* and *B* and *SI Appendix*, Fig. S4*A*) (64, 65), an allosteric inhibitor, led to parasite death in vitro at submicromolar concentrations (18). However, there have been numerous inhibitors developed to target the human enzyme (Fig. 3*A*) (66) that remain untested on schistosomes, such as an additional allosteric inhibitor, UPCDC-30245 (67, 68), and a physiological inhibitor, Eeyarestatin I (EerI) (69, 70). We revisited these compounds and tested them on adult parasites, finding that they were all effective, and produced similar physical phenotypes (Fig. 3 *A* and *B* and *SI Appendix*, Fig. S4*A*). We assessed on-target action as described previously (18), performing a western blot on drug-treated worm lysate using an antibody that recognizes Lys48 (K48) polyubiquitinated proteins. Like before, we observed an accumulation of proteins targeted for proteasome-mediated degradation for known controls CB-5083 and NMS-873 as expected (Fig. 3*C* and *SI Appendix*, Fig. S4*B*), meaning the function of p97 has been perturbed following drug treatment. However, we did not observe the same accumulation for CB-5083 analogs, DbeQ and ML240, despite seeing activity on the worms. ML240 killed parasites quickly, which could circumvent detectable ubiquitin accumulation. But this suggests that there might be another target in schistosomes for these inhibitors that could lead to parasite death, as is known to be true for human cells (71) and other organisms (72). In addition, we did not see significant increases in K48 abundance with UPCDC-30245 or Eer1 (Fig. 3*C* and *SI Appendix*, Fig. S4*B*). p97 governs diverse pathways separate from protein degradation via the ubiquitin proteasome system (21, 73). Certainly, UPCDC-30245 has been shown to have a unique mechanism of action in blocking endo-lysosomal degradation (74, 75) and exerts only weak effects on protein ubiquitination.

Since *S. mansoni* (804 amino acids) and *H. sapiens* (806 amino acids) p97 possess 82% identity in their amino acid sequence, we wanted to determine whether there were any empirical differences in selectivity when treated with inhibitors. We purified both full-length *S. mansoni* and *H. sapiens* p97, then measured the IC_50_ of described inhibitors (Fig. 3 *A* and *D*) using a luminescence-based biochemical assay, Kinase-Glo, which measures ATP consumption. We determined that, for inhibitors related to the scaffold of CB-5083, there was some preference for the schistosome enzyme (Fig. 3*D*). This selectivity was minor for CB-5083 (~twofold), but more pronounced for ML240 (~fivefold). However, we were hesitant to repurpose this set of compounds for treatment of schistosomiasis, as treatment with CB-5083 in Phase I clinical trials for cancer led to undesired, off-target effects (71, 76). Unfortunately, both allosteric inhibitors, UPCDC-30245 and NMS-873 compounds were selective in favor of the human enzyme (Fig. 3*D*). To further our ability to conduct target-based drug discovery, we obtained a structure of *S. mansoni* p97 using cryo-EM (Fig. 3*E* and *SI Appendix*, Fig. S5 and Table S6). Overall, we found minor changes in the active site of schistosome p97 compared to its human homolog. The main difference being an asparagine (Asn652) in the place of an alanine (Ala655) in the human enzyme that could contribute to selectivity (Fig. 3*E*). To ascertain whether there were any subtle differences in the conformational changes of schistosome p97 compared to its human ortholog, we determined the structure of the *S. mansoni* p97 apo-enzyme (*SI Appendix*, Figs. S6*A* and S7*A* and Table S6) and its complex bound to ATPγS, a slow-hydrolyzing analog of ATP (*SI Appendix*, Figs. S6*B* and S7*B*). We found that the binding modes and conformational changes observed in *S. mansoni* p97 following binding to active site inhibitors (CB-5083) (Fig. 3*E*) and nucleotide substrates (ATPγS) were similar to the human enzyme (67, 77) (*SI Appendix*, Figs. S7 *A*–*C* and S8 *A* and *B*). Indeed, there is a conserved mechanism in a pivot-like movement of the D2 domain and subsequent central pore constriction (~8 Å) following ATPγS nucleotide binding (67) (*SI Appendix*, Fig. S8 *A* and *B*).

### High-Throughput Screen of *S. mansoni* p97 Uncovers Benzoxazole Propiolamide Covalent Inhibitor Scaffold.

**Fig. 4.**
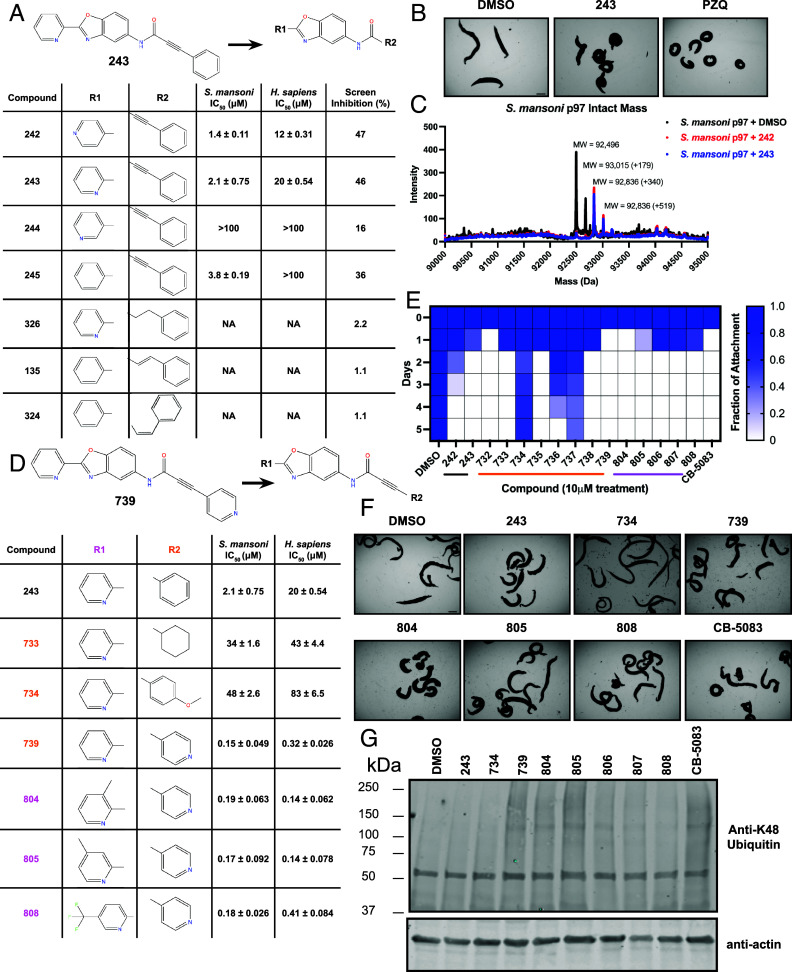
A covalent scaffold identified in high-throughput screen for inhibitors of schistosome p97 yields drug candidates that kill worms. (*A*) Chemical structure of schistosome p97 inhibitor benzoxazole propiolamide analog 243 and its related molecules identified from a high-throughput screen of the UT Southwestern 350 k library. Structural activity relationship between analogous active and inactive compounds included in the high-throughput screen. Compounds were retested to determine the potency against parasite (Smp97) and human (Hsp97) enzyme and confirm selectivity. A value of >100 signifies that the IC_50_ value was predicted to be greater than 100 μM or the maximal inhibition was not achieved by concentrations included in dose–response experiments (100 μM maximum). NA signifies that these compounds did not produce any notable effect on the enzyme at the tested concentrations (100 μM to 1 nM). Values were calculated using Prism. (*B*) Light microscopy images of worms treated with compound 243 compared to DMSO (negative) and praziquantel (positive) control. (*C*) Mass spectrum of recombinant *S. mansoni* p97 in solution with DMSO control (black) or covalent scaffold compounds (242—red and 243—blue). Peak migrations account for a single p97 monomer (~92.5 kDa) and a mass shift following incubation with either covalent inhibitor (339.35 Da) or DMSO. Additional species are detected at 179 Da for either peak. (*D*) Chemical structure of compound 739. Structural activity relationship outlining R1 (800 series) and R2 (700 series) modifications made to the depicted scaffold. Comparative IC_50_ values display the potency of each compound on the recombinant parasite and human enzyme (100 μM to 1 nM). Values were calculated using Prism. (*E*) Heat map showing attachment of a population of 10 adult worms in a culture well over time following treatment with compounds for 72 h, refreshing drug and media every 24 h, then monitoring for phenotypes until the end of the experiment at D5. A value of 1 (dark blue) means that all worms were attached to tissue culture substrate, and a value of 0 (white) denotes that all worms were detached from the plate. (*F*) Light microscopy images of worms treated with inhibitors from this chemical series compared to DMSO negative control and positive control CB-5083, a known p97 inhibitor that is active on adult schistosomes. (*G*) Western blot depicting polyubiquitinated protein profile (K48 antibody) in worm lysate following treatment by DMSO control or p97 covalent inhibitor analogs (actin loading control). [Scale bar (*B* and *F*), 1,000 μm.]

These findings inspired us to conduct a high-throughput screen of *S. mansoni* p97. This would provide the best opportunity to identify scaffolds for enzyme inhibition that are specific for the parasite p97, rather than re-engineering existing compounds that are toxic or display no selectivity. So, we conducted a high-throughput screen of roughly 350,000 compounds (*SI Appendix*, Table S3). We were intrigued by one scaffold, as many compounds bearing a similar scaffold were included in the screen, but only four of the series showed marked inhibition of *S. mansoni* p97 function (Fig. 4*A*). Each active compound possessed the same benzoxazole propiolamide moiety, while the corresponding alkenyl (Z—324 and E—135), or alkyl amide (326) were inactive (Fig. 4*A*). We validated the activity of these compounds on the enzyme by performing dose–response analysis and found ~10-fold selectivity for the parasite enzyme over its human counterpart (Fig. 4*A*). After determining their potency, we treated adult worms with these inhibitors, and three of the four parent compounds were found to cause at least detachment in the parasites after 3 d of treatment with 10 μM compound (Fig. 4*B* and *SI Appendix*, Fig. S9*A*). Two compounds, 242 and 243, led to death in adult parasites (Fig. 4*B* and *SI Appendix*, Fig. S9*A*), 243 being the more potent of the two (*SI Appendix*, Fig. S9C). However, we did not see K48 polyubiquitinated protein accumulation by western blot following 48 h of treatment with these initial inhibitors (*SI Appendix*, Fig. S9*B*), which is expected for the majority of described p97 inhibitors.

As the electrophilic propiolamide group is an essential part of the pharmacophore for p97 inhibition, we hypothesized that these molecules were covalently modifying p97. We confirmed this suspicion by performing intact mass spectrometry and found a shift in abundance at the expected sizes of these molecules (~339 Da) (Fig. 4*C* and *SI Appendix*, Fig. S10 *A* and *B*). A trypsin digest followed by mass spectrometry revealed that the dominant species modified by this scaffold was a peptide ([K].GVLFYGPPGCGK.[T]) containing Cys519, which resides within the ATP binding pocket of p97 (*SI Appendix*, Figs. S7*D* and S10*C*). However, other cysteines along p97’s structure do appear to be modified by this chemical series, albeit at much lower levels (>100-fold). Covalent modification of this residue would explain the inhibition of p97’s ATPase function, as other described inhibitors that modify this residue are effective inhibitors of p97’s capacity for ATP hydrolysis (64).

### Structure–Activity Relationship Analysis Generates Potent Inhibitors of Schistosome p97.

We sought to improve the potency of these compounds and conducted a limited structure–activity relationship (SAR) analysis on this chemical series (Fig. 4*D* and *SI Appendix*, Fig. S9*C*). There were two primary sites where modifications were made to this parent scaffold. The first modification site is the benzene ring proximal (**R2**) to the propiolamide moiety, and the second is the pyridine ring distal to the same propiolamide moiety (**R1**). Compounds belonging to the 700 series (700s) contain alterations made to the **R2** chemical group, while 800 series (800s) compounds possess **R1** modifications.

We first tested the 700 series of compounds, containing modifications to the **R2** benzene ring. We found that adding electron withdrawing groups, such as a chloride, or an electron donating group, such as a methoxy, to the proximal phenyl group (**R2**) reduced potency on the enzyme (Fig. 4*D* and *SI Appendix*, Fig. S9*C*). Swapping the **R2** benzene ring with another nonaromatic cyclic group, like a cyclopropane or cyclohexane, also reduced potency. Altering this benzene ring into a pyridine increased potency, giving us our most potent enzyme inhibitor, 739 (IC_50_ = 150 nM). Then, we tested the 800 series of compounds, determining how modifications to the **R1** pyridine ring affected the potency of these compounds on the enzyme. Adding a hydrophobic methyl group and moving it around the **R1** aromatic pyridine ring had little impact on potency regardless of its position. However, each of these later **R1**-modified compounds displayed submicromolar potency on the enzyme and produced the desired pharmacologic effects by western blot after treating adult parasites with the inhibitors (Fig. 4*G* and *SI Appendix*, Fig. S9 *B* and *C*).

Promisingly, the SAR trends mimicked the potency of these compounds on worms (Fig. 4 *D*–*F* and *SI Appendix*, Fig. S9 *A* and *C*). We treated adult worms with each of these compounds at 10 μM to determine preliminary activity and tracked their attachment to tissue culture substrate as well as their physical morphologies (Fig. 4 *E* and *F* and *SI Appendix*, Fig. S9*A*). From the parent scaffold, only compounds 242 and 243 showed significant activity on worms, causing detachment within 48 h in culture, ultimately leading to their death. While compound 245 did cause detachment, it did not lead to parasite death. Compound 244, which presented minimal activity on the enzyme, did not have any adverse effects on worm health and survival.

Modifications to the **R2** group (700 series) had some variable effects on parasites. Several alterations to the scaffold that decreased potency on the enzyme (734, 736, and 737) also led to decreased activity on the worms (Fig. 4*E* and *SI Appendix*, Fig. S9 *A* and *C*). And the most potent compounds on the enzyme, 738 and 739, had the most potent effects on worms (*SI Appendix*, Fig. S9*C*), ultimately leading to death of the parasites following detachment within 24 h. However, only compound 739 showed on-target activity by western blot, producing the predicted accumulation of K48 polyubiquitin moieties following inhibitor treatment (Fig. 4*G* and *SI Appendix*, Fig. S9*B*). Some compounds of this **R2** modification chemical series (732, 733, and 735) were still active on worms despite abolishing activity on the enzyme. These off-target effects suggest that there might be another molecular target of these compounds, as is true for CB-5083 (71, 76), or pharmacokinetic properties of the compounds (i.e., solubility) might obfuscate the activity seen on parasites.

Each of the compounds with modifications to the **R1** group (800 series) displayed submicromolar potency on the enzyme, produced an on-target biological response in the accumulation of K48 polyubiquitin chains, and killed parasites (Fig. 4 *D*–*G* and *SI Appendix*, Fig. S9). With these later 800 series compounds, we found that there was a strong correlation between potency on the enzyme in vitro with an impact on the worms, supporting an on-target killing mechanism (Fig. 4 *E* and *F* and *SI Appendix*, Fig. S9 *A* and *C*).

We measured the IC_50_ of each of these covalent inhibitor analogs on the human p97 at the same time as the schistosome enzyme. Our data suggest that, as the potency of these compounds increased for the parasite p97, the selectivity for the parasite enzyme over its human counterpart decreased (Fig. 4*D* and *SI Appendix*, Fig. S9*C*). Unfortunately for these covalent inhibitors that appear to have an on-target killing mechanism (800 series and compound 739), there is little to no selectivity for the parasite enzyme (*SI Appendix*, Fig. S9*C*). After seeing these changes to selectivity, we tested these compounds on human cells to determine cytotoxicity (*SI Appendix*, Fig. S11 *A*–*C*). We treated HepG2 cells with the parent compound (243), the most potent compound on the enzyme (739), and each of the 800 series compounds to determine cytotoxicity, using CB-5083 as a control. Each of these compounds were cytotoxic in roughly equal concentrations to the potency (EC_50_) of the compounds on worms (*SI Appendix*, Fig. S11*E*). However, they were noticeably less cytotoxic to cells than CB-5083.

### Cryo-EM Structure of *S. mansoni* p97 Bound to Covalent Inhibitors Reveals Conformational Change.

**Fig. 5.**
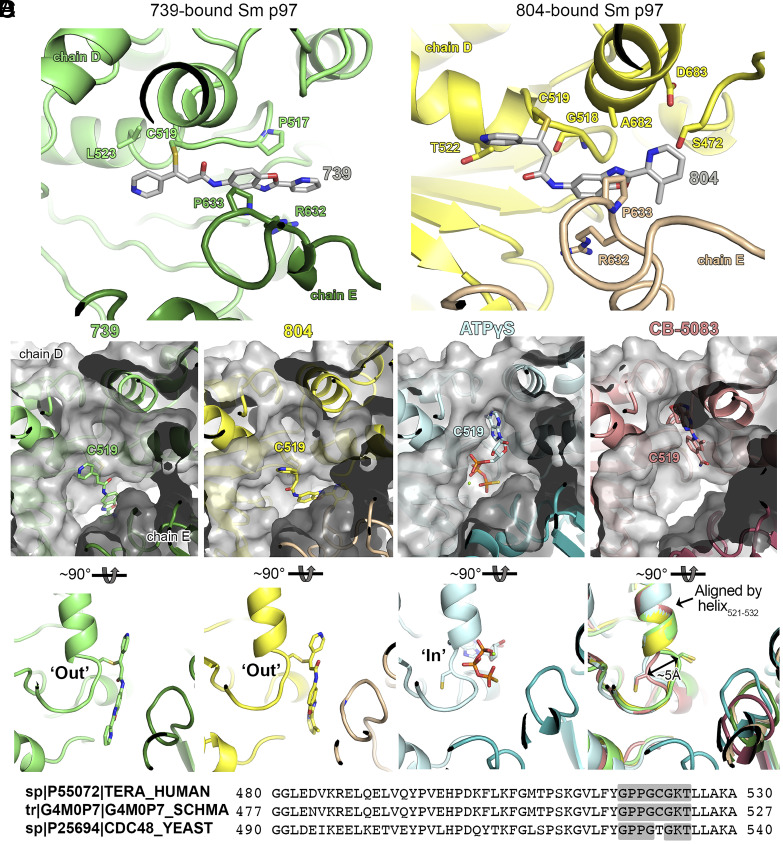
Cryo-EM Structure of *S. mansoni* p97 bound to covalent scaffold. (*A*) Binding pocket of covalent inhibitors 739 (green) and 804 (yellow) within the D2 domain of *S. mansoni* p97 and the adjacent monomer. Potential contact residues of compounds 739 and 804 are shown in sticks. (*B*) Binding pocket of covalent inhibitors 739 (green) and 804 (yellow), shown as gray surfaces, relative to known ligands, ATPγS (blue) and CB-5083 (pink). (*C*) Positioning of P-loop in the “In” or “Out” position within the D2 domain of the schistosome p97, aligned by the indicated alpha helix (residues 521 to 532). (*D*) Sequence alignment of the conserved human and schistosome P-loop within the D2 walker domain of p97 compared to a yeast homolog.

To better understand where these compounds bind p97 in the hopes of improving selectivity, we sought to solve the structure of this scaffold bound to *S. mansoni* p97 using single particle cryo-EM analysis. Two cryo-EM datasets were collected for benzoxazole propiolamide inhibitor analogs 739- and 804-bound to schistosome p97 (Fig. 5*A* and *SI Appendix*, Figs. S6 *C* and *D* and S12 *A* and *B* and Table S6). We identified density belonging to these inhibitors at an interface between two monomers of p97 (Fig. 5 *A* and *B* and *SI Appendix*, Figs. S6 *C* and *D* and S12 *A* and *B*). As suggested by our mass spectrometry experiments, Cys519 appears to be covalently linked to these molecules (Fig. 5*A*). Presumably, this occurs through a Michael reaction conducted by the thiol of Cys519, as the entire mass of the inhibitor can be detected by intact mass spectrometry (Fig. 4*C* and *SI Appendix*, Fig. S10*A*). This suggests that there is no leaving group in this chemical reaction and instead forms a carbon–carbon double bond in addition to the covalent linkage to Cys519 (*SI Appendix*, Fig. S12*E*). Based on our best approximation, 739 and 804 appear to engage in slightly different binding contacts (Fig. 5*A* and *SI Appendix*, Fig. S12 *C* and *D*), even though they only differ by a single methyl group on the distal (**R1)** pyridine ring. For instance, compound 804 makes more putative contacts with residues in its conjugated p97 monomer (**chain D**). Additionally, compound 739 lays flat within its binding pocket, while compound 804 is rotated around the carbon–carbon double bond so that the thiol of Cys519 forms an adduct on the opposite side of the amide (Fig. 5*A* and *SI Appendix*, Fig. S12 *C*–*E*). In the case of 739, it is on the same side. This means that 804 forms an E-configuration olefin, while 739 forms an elongated Z-configuration olefin following nucleophilic attack by Cys519 (*SI Appendix*, Fig. S12*E*), enabling these compounds to engage different residues within their binding pocket. However, given the resolution of the maps and density for these inhibitors, we cannot rule out that these compounds may be bound in a different configuration.

These molecules occupy an adjacent, and overlapping, binding site to CB-5083 and ATPγS (Fig. 5*B*). As a result of covalent conjugation, the flexible loop where Cys519 resides is displaced to an outward (out) position (Fig. 5*C*), which points toward the neighboring subunit. Consistent with the apo and CB-5083-bound states, the conformation of the rest of the enzyme apart from this loop remains unchanged (*SI Appendix*, Fig. S8 *A* and *B*). Cys519 is positioned within a flexible “P- loop” of the Walker A motif (GxxxGKT) in the D2 domain of p97, which appears to be broadly conserved across multicellular eukaryotes, but not certain unicellular organisms such as yeast (*S. cerevisiae*) (78) (Fig. 5*D*). Walker motifs are sequences involved in binding to nucleotides and coordinating their hydrolysis (79). Indeed, the amino backbone of Cys519 is involved in binding ATPγS in both the human (77) and schistosome protein (*SI Appendix*, Fig. S7 *B* and *D*). Cys519 is flanked by a conserved region of flexible amino acids [GPPG**C**G] that may enable the loop to move freely depending on its physiological context. Interestingly, Cys519 naturally occupies an inward (in) position in its apo-form, when bound to nucleotide substrate (ATPγS), or active site inhibitors (CB-5083) (Fig. 5*C*). However, when it binds to these benzoxazole propiolamide inhibitors, the Cys519 moves into an outward (out) position. While the sulfhydryl sidechain of Cys519 is not utilized in the catalysis of nucleotides, the modification of this residue, and the subsequent conformational change of the loop where it resides (Fig. 5*C*), locks the backbone of Cys519 in a position where it can no longer make the contacts required for catalysis. This conformational change would render the enzyme incapable of binding both the inhibitor and ATP.

This is a conformational change that, to the best of our knowledge, has not been seen for structures of p97 deposited in the PDB (49, 50, 67, 74, 77, 80, 81). But equivalent conformational changes are well documented for protein kinases that bind Type II inhibitors (82–84). Kinase activation loops contain a conserved Asp-Phe-Gly (“DFG”) motif. In an active conformation, the Asp within this motif points inwardly to the ATP-binding pocket and assists in coordination of Mg^2+^ ions. In an autoinhibited state, this same Asp flips ~180° to an outward position in a shift that is ~5 Å from the ATP binding site. The shift seen from Cys519 in p97 is also roughly a 5 Å movement that forms the new allosteric pocket adjacent to the ATP binding pocket (Fig. 5*C*). The in/out switch we see in this context for p97 mimics a DFG flip in protein kinases. This represents a significant finding, where new classes of inhibitors can be developed for this allosteric binding pocket of p97 for the numerous disease states (cancer, Alzheimer’s, etc.) it is involved in across species.

Visualizing the binding of this compound further explained the trends we saw with SAR (Fig. 4*D* and *SI Appendix*, Fig. S9*C*). The pocket where these compounds bind is narrow, so there is little room for bulky substituents (Fig. 5 *A*–*C*). These observations suggest the addition of functional groups to the proximal aromatic group (**R2**) decreased potency as this likely interfered with its fit into the pocket, clashing with sidechain density. However, the distal aromatic group (**R1**) is facing solvent, so the addition of other functional groups, even bulky ones, did not significantly reduce the potency of the scaffold. This binding site has been explored with docking experiments (65, 85) and is confirmed to be similar for our scaffold using cryo-EM. Similar to how the discovery of the DFG flip led to the discovery of new classes of inhibitors in kinases, our covalent compounds reveal an opportunity to develop inhibitors that target this unique allosteric pocket in p97.

Both p97 and MAT2A serve as prime examples for our proposed pipeline, where utilizing the human druggable genome as a bioinformatic template yields high-value drug targets in schistosomes. Focusing on these candidate drug targets that bear homology to established human drug targets enhances our chance of success by providing ready access to a wealth of FDA-approved and commercially available small molecule chemical scaffolds for testing on adult parasites as well as established protocols for recombinant expression, purification, and biochemical assay to identify novel inhibitors. Furthermore, even with highly similar molecular targets, such as p97, we have shown that it is possible to identify selective scaffolds that are capable of killing schistosomes. This suggests that this workflow is a viable method and stands as a foundational success for drug target discovery in other organisms with few molecular tools available.

## Discussion

### Schistosome p97 as a Therapeutic Target.

p97 is a pivotal constituent of the UPS that extracts and disassembles its substrates from various cellular compartments. The discovery of p97 inhibitors has provided not only basic research tools, but an avenue for effective therapies in the clinic for cancer and neurodegenerative diseases. Here, we have described a benzoxazole covalent inhibitor series that not only kills adult schistosomes through an on-target mechanism by interfering with UPS integrity but also revealed unique biology regarding a previously unreported p97 allosteric inhibition mechanism. These findings would traditionally provide a boon to antiparasitic drug discovery efforts that would necessitate further characterization of the physiochemical and pharmacokinetic properties of this inhibitor scaffold (e.g., bioavailability, solubility, metabolic stability, etc.) in the hopes of moving into an in vivo model. However, we have demonstrated that these compounds are toxic to a HepG2 human cell line in roughly equivalent concentrations to their potency on adult parasites. Given this, further optimization is required for this chemical scaffold to achieve a more favorable selectivity profile, which would pave the way for impactful in vivo studies.

Our data suggest that there are further opportunities for p97 as a drug target for the treatment of infectious parasitic diseases such as schistosomiasis. Despite there being significant identity between the human and schistosome enzyme (~82%), we have identified numerous other selective compounds for the schistosome enzyme over its human counterpart that kill parasites. Outside of the covalent scaffold explored in this paper, many of these other scaffolds uncovered through our high-throughput screening efforts bear good chemistry (Lipinski’s rule of 5, Veber’s rule, etc.) (45–48) and display on-target activity in adult worms. In addition, there are numerous binding locations where selective chemicals can be developed even if the active site is mostly conserved. Indeed, we have observed decreased potency for the allosteric inhibitors, NMS-873 and UPCDC-30245, on the schistosome enzyme in vitro compared to the human enzyme. This empirical evidence implies that compounds might be engineered to create compounds where the inverse is true, being potent for the schistosome enzyme rather than the human. Not only that, but we have confirmed there are additional allosteric binding sites where molecules can bind, such as the one for covalent inhibitors, 739 and 804, at the interface between two monomers of p97 described in this paper.

Interestingly, elucidating the binding pocket of these benzoxazole propiolamide inhibitors has uncovered an inhibitory mechanism where the flexible nucleotide binding motif within the D2 domain of p97 can be locked in an out position, distinct from its typical in conformation. This provides insights into the potential mechanistic activity of p97 in its native state and its ability to sense substrates using this flexible motif. Presumably, this conformational change mimics a physiological capacity. We hypothesize that this loop is involved in substrate sensing and “breathes” in a naturally flexible state, but the predominant species is found in the in conformation as seen with the apo enzyme or nucleotide-bound state (Fig. 5*C*). p97 is likely constitutively bound to ATP or ADP as the natural concentrations of these substrates are high (0.5 to 5 mM) in its cellular context (86–88). Indeed, most purified recombinant p97 has been found to be prebound to nucleotide that must be removed by heat or apyrase treatment (23, 81, 89–91). However, our enzyme was purified in its apo state, suggesting that the allostery of nucleic acid binding may be different between the human and schistosome enzyme. Flipping into an out conformation could be important for releasing ATP and/or ADP, or for binding to another substrate or cofactor. Alternatively, the flexibility of this motif could be important for intersubunit communication between monomers or domains as is known to occur within p97 (77, 81, 90, 92). Regardless, binding to this scaffold has revealed a unique conformational change in p97 that could uncover more biology of the hexameric ATPase and lead to further development of therapeutics that target it. While our own covalent scaffold was cytotoxic on human cells comparable to activity on adult worms (*SI Appendix*, Figs. S9*C* and S11*E*), it does not mean that more selective inhibitors cannot be developed in the future that target this pocket. And it would be intriguing to investigate whether noncovalent scaffolds could also access this allosteric binding pocket. Furthermore, p97 is evolutionarily conserved across phyla, and the more removed from metazoans, the more unique differences are present in the structure of the enzyme. This leads to greater opportunities to discover selective therapeutics for the treatment of disease.

### Other Potential Drug Targets.

For the purposes of this paper, we have focused primarily on p97 and MAT2A to illustrate the benefits of the outlined prioritization criteria in taking an unbiased approach to pursuing potential schistosome drug targets. However, 65 potential targets scored well in our criteria (cumulative score ≥ 10), and 51 of them possessed favorable structural data (PSS ≥ 2) that suggests selective inhibitors could be identified or synthesized (Dataset S3). Among these were drug targets in various enzymatic categories, such as USP8, a deubiquitylating enzyme, LATS2, a kinase involved in cell proliferation, ADAM17, a peptidase involved in cell signaling, and others. Among those possessing favorable structural data, there are several targets where a nucleophilic residue has replaced a more inert residue present the human ortholog. In the schistosome orthologs of PLD2 and MAT2A, cysteines were located within binding pockets that are not present for their human counterparts (Fig. 2*F* and *SI Appendix*, Fig. S3*B*). These residues could not only confer selectivity, but provide a means for targeted covalent strategies to modulate activity.

Many of the potential drug targets identified from RNAi experiments, however, were found to fall below a threshold of 10 when compiling the scores from each individual category. The primary reason for this was the candidates lacked ideal characteristics from two or more of the listed categories. For example, subunits of the proteasome were listed below this threshold, thus deprioritized from future studies. However, it is well known that proteasome inhibitors are effective against many different types of disease, including parasitic infections (93). Schistosomes are present among those that can be killed by proteasome inhibitors like bortezomib (18). However, it is essential in human cells and difficult to recombinantly express the entire proteasome, thus it must be purified from entire worms. While the feat has been done (94), it remains to be seen whether it can be done at a scale that enables high-throughput screening of hundreds of thousands of compounds against the enzyme complex. Alternatively, a significant amount of time can be contributed to optimizing an appropriate recombinant system for purification. Therefore, the proteasome is likely a viable target, but not ideal for a ready-made drug discovery campaign.

### Final Thoughts.

Drug discovery is a rapidly changing landscape, where nontraditional methods of chemical intervention are revealed each year. As such, it would be inappropriate to completely dismiss a single gene encoding an essential protein from becoming a therapeutic target. It is our goal, however, to provide sound data on which potential drug targets provide the path of least resistance for a drug discovery campaign to develop alternative treatments to schistosomiasis. This paper provides a valuable resource to the community to enable the rational discovery of alternative therapeutics to the current standard of care, praziquantel.

## Materials and Methods

Detailed materials and methods are described in *SI Appendix*, *Materials and Methods*.

### Worms and Culture.

Adult *S. mansoni* (NMRI strain) (6 to 7 wk post-infection) worms were harvested from infected female Swiss Webster mice as described previously (18). Experiments with and care of vertebrate animals were performed in accordance with protocols approved by the Institutional Animal Care and Use Committee (IACUC) of UT Southwestern Medical Center (approval APN: 2017-102092).

### Bioinformatic Identification of Potential Drug Targets, Primer Design, and Large-Scale RNAi.

To identify druggable genes within schistosomes, we compiled known human drug targets from databases such as the Therapeutic Target Database (TTD) (26), Drug-Gene Interaction Database (DGIdb) (95), ChEMBL (25), and DrugBank (24). Drug targets were also pulled from literature reviews (96), prioritizing kinases as potential drug targets (97, 98). Identifiers (Ensembl and Uniprot) (99, 100) were used in combination with BLASTp (29–31) to establish schistosome genes with similarity to these drug targets (Schistosome proteome: PRJEA36577) (Human Proteome: Homo_sapiens.GRCh38). Once schistosome accessions were retrieved, we removed any IDs with an e-value > 0.0001. Then, we filtered out duplicate IDs or those that have previously been cloned by the Collins Lab (18). Next, we removed any IDs that did not possess a predicted catalytic activity according to GO terms (27, 28). Last, remaining IDs were prioritized and included for screening if they had >10 transcripts per million (TPM). Raw and processed RNA-Seq data for adult male parasites have been deposited in NCBI (**GSE290988**). Primers were designed for target genes and modified for adaptability to large-scale RNAi screening efforts as previously described (18). Unless otherwise noted, all RNAi experiments were performed using previously described methods for large-scale identification and validation of essential genes in adult parasites (18).

### In Silico Prioritization Rationale and Scoring Criteria.

Candidate genes were scored on a numerical scale in whole integers from 0 (unfavorable qualities) to 3 (highly favorable qualities) in each category based on how well they fit the criteria described in *SI Appendix*, Table S1. Their cumulative score determined how suitable any given candidate drug target was for a ready-made drug discovery campaign. Further details on rationale and scoring are described in *SI Appendix*, *Materials and Methods*.

## Supplementary Material

Appendix 01 (PDF)

Dataset S01 (XLSX)

Dataset S02 (XLSX)

Dataset S03 (XLSX)

Dataset S04 (XLSX)

Dataset S05 (XLSX)

## Data Availability

CryoEM density maps and refined atomic models have been deposited in the Electron Microscopy Data Bank (EMDB) with accession numbers EMD-71062 (101), EMD-71063 (102), EMD-70961 (103), EMD-71064 (104), and EMD-71066 (105), and in the Protein Data Bank (PDB) with matching accession numbers of PDB-9P00 (106), PDB-9P01 (107), PDB-9OX9 (108), PDB-9P02 (109), and PDB-9P07 (110) for apo *S. mansoni* p97 and Smp97 in the presence of known ligands ATPγS and CB-5083, and covalent ligands 739 and 804, respectively. RNAseq data have been deposited in NCBI (GSE290988) (111). All other data are included in the manuscript and/or supporting information.
