# Supplementary material for: A genome-scale drug discovery pipeline uncovers therapeutic targets and a unique p97 allosteric binding site in Schistosoma mansoni
Source: Proc Natl Acad Sci U S A. 2025 Aug 29;122(35):e2505710122. doi: 10.1073/pnas.2505710122 (PMC12415213; doi:10.1073/pnas.2505710122)
Supplement: Supplementary file 1 — Appendix 01 (PDF) [file pnas.2505710122.sapp.pdf]

Supporting Information

A genome-scale drug discovery pipeline uncovers new therapeutic targets and a unique p97 allosteric binding site in *Schistosoma mansoni*

Dylon R Stephens, Ho Yee Joyce Fung, Yan Han, Jue Liang, Zhe Chen, Joseph Ready and James J Collins III

Supplementary Data contain:

Supplementary Materials and Methods

Supplementary References

Supplementary Tables 1 - 3

Supplementary Figures 1 – 12

Supporting Information - Benzoxazole propiolamide inhibitor analog chemical synthesis NMR

Materials and Methods

Worms and culture

Adult *S. mansoni* (NMRI strain) (6-7 weeks post-infection) worms were harvested from infected female Swiss Webster mice by hepatic portal vein perfusion using 37°C DMEM (Mediatech, Manassas, VA), 8% Horse Serum, and heparin. Parasites were rinsed in DMEM + 8% Horse Serum and cultured at 37°C, 5% CO_2 in Basch's Medium[127] and Antibiotic Antimycotic (Gibco/Life Technologies, Carlsbad, CA 92008). Experiments with and care of vertebrate animals were performed in accordance with protocols approved by the Institutional Animal Care and Use Committee (IACUC) of UT Southwestern Medical Center (approval APN: 2017-102092).

Bioinformatic identification of potential drug targets

To identify druggable genes within schistosomes, we compiled known human drug targets from databases such as the Therapeutic Target Database (TTD)[1], Drug-Gene Interaction Database (DGIdb)[2–4], ChEMBL[5], and DrugBank[6]. Drug targets were also pulled from literature reviews[7], prioritizing kinases as potential drug targets[8,9]. Identifiers (Ensembl and Uniprot)[10,11] were used in combination with BLASTp[12–14] to establish schistosome genes with similarity to these drug targets (Schistosome proteome: PRJEA36577) (Human Proteome: Homo_sapiens.GRCh38). Once schistosome accessions were retrieved, we removed any IDs with an e-value > 0.0001. Then, we filtered out duplicate IDs or those that have previously been cloned by the Collins Lab[15]. Next, we removed any IDs that did not possess a predicted catalytic activity according to GO terms[16,17]. Lastly, remaining IDs were prioritized and included for screening if they had > 10 transcripts per million (TPM). Raw and processed RNA-Seq data for adult male parasites have been deposited in NCBI (**GSE290988**).

Large-scale RNAi screen

For our screen, we designed primers to amplify ~700bp (500-1000bp) fragments using BatchPrimer3 (http://batchprimer3.bioinformatics.ucdavis.edu/index.html). If genes were shorter than 700bp, primers were designed to cover as much of the transcript as possible. To enable RNA synthesis via reverse transcription, we added a T7 promoter (GAATTTAATACGACTCACTATA) sequence to the 5' end of each oligo. Following the T7 site, and flanking the gene-specific sequence, we inserted a NotI (GC^GGCCGC) and AscI (GG^CGCGCC) restriction enzyme site, respectively, to facilitate DNA sequencing of amplified cDNAs to validate RNAi

phenotypes observed with the encoded sequence. Oligos were synthesized and prepared in 96-well format. Target genes were amplified using PCR from adult schistosome mixed sex cDNA template. 5 μL of PCR product was used for *in vitro* transcription (IVT) to generate a total volume of 100 μL dsRNA. IVT reactions proceeded at 37°C overnight, then underwent successive 3-minute annealing steps at 95°C, 75°C, and 55°C, finally cooling to room temperature until use or storage at -20°C. Successful synthesis was verified using agarose gel electrophoresis to determine presence and size of PCR products and resulting dsRNA. RNAi experiments were conducted on approximately 5 adult worm parasite pairs (or 5 adult males) in 12-well plates. Worms were cultured in 3 mL Basch 169 media and treated with 20 μL dsRNA on D0 and D2, then every 7 days afterward (D9, D16, and D23). Experiments finished on D30, where videos were captured using light microscopy (Axio Zoom V16) to document visible phenotypes that manifested during the treatment. Media was changed every 1-2 days, where worm attachment, morphological changes, and any other aberrant observations were recorded in addition.

The identity of hits from the RNAi screen were confirmed by digesting PCR products using NotI (NEB) for 30min at 37°C. DNA bands were purified from agarose gels using Zymoclean Gel DNA Recovery Kit, then sequenced using a T7 primer. For hit validation, primers of non-overlapping gene fragments were designed using BatchPrimer3. In the case of gene sequences too short to design non-overlapping primers, the original primers were used in the absence of initial modifications (T7, NotI, and AscI) added to facilitate large-scale IVT. Amplified PCR products using these genes were inserted into pJC53.2 using TA cloning. Plasmids from positive clones were purified from *E. coli*, then sent for sequencing to verify identity of the inserted constructs. Upon confirmation, these plasmids were used to generate dsRNA to repeat RNAi using the same treatment schedule as before. Hits were only considered validated if they displayed similar, fully penetrant phenotypes in three independent experiments using biologically unique batches of dsRNA and worms.

***In silico* prioritization rationale**

We prioritized potential drug targets in schistosomes based on the rationale that 1). Complete debilitation of parasites upon interfering with a target fares best for host clearance (RNAi Severity), 2). Non-essential homologs in mammals (human and mouse) provide the widest therapeutic window (Mammalian Essentiality), 3). Encoded proteins should be expressed and purified in recombinant systems in sufficient quantity to be measured reliably

in high-throughput format to pursue a target-based drug discovery method (Assayability), 4). Potential targets should possess features that can bind drug-like molecules (Druggability), and 5). Sequence and 3D alignment data should supply unique amino acid differences in critical binding regions (Parasite Selectivity). Candidate essential genes were scored on a numerical scale in whole integers from 0 (unfavorable qualities) to 3 (highly favorable qualities) in each category based on how well they fit the criteria described above. Their cumulative score determined how suitable any given candidate drug target was for a ready-made drug discovery campaign.

RNAi severity

It is reasonable that targets whose RNAi phenotypes occur rapidly and with the most deleterious effects represent the most attractive targets for the development of therapeutics. Therefore, we have prioritized targets based on the time a phenotype manifested relative to the start of the experiment, the severity, and number of phenotypes that manifested (**Dataset S1**). Highest priority (a score of 3) was given to essential genes whose phenotypes began to manifest within the first two weeks (D1-D15) of the 30-day RNAi experiment and whose knockdown produced more than one morphological defect (tissue/gut edema, tegument/head degeneration, hypercontraction, death, etc.). Next, a score of 2 was attributed to essential genes who had more than one phenotype as listed above but occurred later in the experiment schedule (D16-25). A score of 1 was given to essential genes who had a modest phenotype (i.e. detachment only) that manifested at an intermediate point in the experiment (D16-25). A score of 0 was given to essential genes producing a modest phenotype late in the RNAi experiment (D25-D30). Attachment and morphologies were compared to negative vector controls (treated with pJC53.2 IVT product encoding the bacterial gene *CcdB*).

Mammalian essentiality

The current standard for treatment of schistosomiasis, PZQ, poses minimal toxicity to humans, while effectively clearing hosts of parasite burden[18]. Thus, it is vital that an alternative drug provide a large therapeutic window. We reason that targets essential for schistosomes, but dispensable for mammalian (human and mouse) survival are most likely to provide this therapeutic window. Using our initial bioinformatic searches for schistosome genes with high similarity to drug targets, we have defined human and mouse orthologs using data available in Wormbase Parasite[19] and BLAST[12–14] (**Dataset S3**). Then, we examined these orthologs in the Online Gene

Essentiality Database[20], which catalogs essentiality information from both mouse knockout studies and RNAi/CRISPR screening panels of human cell lines. We have given each target a score for each human and mouse essentiality, giving priority to targets whose orthologs are non-essential following mouse knockout and disruption in human cell lines. The highest possible score (3) was given to targets who are non-essential in mice and human cell lines. A score of 2 was awarded to any candidate whose homolog was essential in some human cell lines, but not others, or essential in mice, yet did not lead to embryonic lethality. If no information was available for mice and human cell lines, then a potential drug target was given a score of 1. The lowest score (0) was given to candidates whose homologs were essential in humans and mice.

Assayability

Target-based screening is an attractive avenue for the identification of small molecules for drug discovery, especially for those targets whose functions have been thoroughly vetted within their cellular context. It provides a wealth of scaffolds that can engage the target, and is also economically feasible, as large quantities of recombinant protein can be readily purified from other organisms like *E. coli* or insect cells. We have given priority to targets bearing homology to proteins that are documented to be well-expressed recombinantly, solubly, and in their native fold. Furthermore, these ideal targets must possess a predicted biochemical activity that can be measured using a commercially available, high-throughput amenable assay and readout (absorbance, colorimetric, fluorescence, luminescence, etc.). Extensive research has been done in literature to determine if a target protein's ortholog has been purified and if its biochemical activity has been measured using commonly available reagents and platforms. References have been provided for assays and purification schemes of human orthologs that have been identified (**Dataset S3**). We have prioritized targets with orthologs that have historically been purified from recombinant systems in sufficient amounts to perform a large compound library screen, and whose activity can be measured using an easily adaptable, commercially available assay. The most ideal potential drug targets were awarded a score of 3 if there were established purification schemes for a given target ortholog, and a method existed to measure its biochemical activity using a robust, commercially available assay. A score of 2 was given if target purification and assay could be achieved with minimal optimization. This means that the target can be purified, and its activity assayed. However, there are additional steps that may require some

optimization, but can generally be achieved with products that can be purchased commercially or protocols that exist and need to be adapted slightly. A lower score (1) was given if purification and assay could be achieved but required extensive optimization, meaning protocols need to be developed or extensively adapted, and reagents to achieve purification and assay are not available commercially, and may need to be made in-house. The lowest score (0) was given if there was no clear path to recombinant purification of the target, and no currently available way to measure its biochemical activity with amenability to high-throughput format. There are many enzymes that can be purified for which a general assay can be applied to determine if a given molecule is interacting with a protein, such as Isothermal Titration Calorimetry (ITC), Differential Scanning Fluorimetry (DSF), and Surface Plasmon Resonance (SPR). These were rarely considered because we wanted to utilize assays that measured the distinct biochemical activity of a given target protein. Likewise, virtual screening is a powerful method to identify potential ligands for enzymes where there is no simple biochemical assay to utilize for traditional high-throughput screening, yet does not factor into our ranking for potential drug targets.

Druggability

For our purposes of identifying potential drug targets that could yield alternatives of PZQ, it is not sufficient that a gene is essential. Indeed, the gene must also encode a protein whose activity can be modulated by binding a small, drug-like molecule. Here, we define drug-like molecules as small molecules having characteristics that are similar to existing drugs according to well-known criteria such as Lipinksi's rule of 5 and Veber's rules (i.e. LogP < 5, molecular weight < 500 Da, < 10 hydrogen bond acceptors, < 5 hydrogen bond donors, ≤ 10 rotating bonds, < 140 $Å^2$ polar surface area)[21–25]. We have already narrowed our initial search to proteins with high amino acid identity to well-documented drug targets in databases such as ChEMBL, DrugBank, and TTD[1,5,6]. This greatly enhances the chance of identifying a druggable schistosome protein. However, some of these targets represent "theoretical" targets that are heavily implicated in diseases but have never been the subject of drug-discovery campaigns. Others are 'bona fide' drug targets that have undergone drug discovery efforts, with some compounds making it into clinical trials. Therefore, we have assessed the predicted biochemical properties and function of each potential schistosome target to determine if they have features that can bind to drug-like molecules[21–25]. We have also searched these databases for existing compounds that have been developed or identified to bind human

orthologs of these potential schistosome targets. Simultaneously, we have searched through chemical vendors such as Selleck Chem and MedChemExpress to find inhibitors of target protein orthologs. Targets have received priority if these compounds are available commercially, possess drug-like properties, and have shown on-target activity *in cellulo*. Where possible, we have provided references for inhibitors of target protein orthologs (**Dataset S3**). If a potential schistosome drug target has similar properties to its human ortholog, and there are one or more one drug-like compounds catalogued in databases such as DrugBank[6] or on commercial vendor websites such as MedChemExpress, then it was given a score of 3. A score of 2 was given if a candidate target was similar to its druggable ortholog, and there were described inhibitors, but these compounds were not drug-like in nature[21–25]. A target was awarded a score of 1 if a candidate target was similar to its druggable homolog, but there were no inhibitors described. Lastly, a potential target was given a score of 0 if it was only weakly related to a druggable target in humans.

Parasite selectivity

As many of our potential targets are derived from the [Human] "Druggable Genome", it is important to confer specificity where available to reduce potential toxicity. Utilizing resources such as AlphaFold[26] and Clustal[27,28], we have identified drug targets that supply unique differences between the parasite and mammalian ortholog. This enables us to compare sequence alignments and 3D structural information available in the Protein Data Bank (PDB)[29,30] to determine inherent selectivity between these enzymes. We have used these databases to compare protein sequence alignments and generate homology models to determine predicted similarity of active sites and potential allosteric pockets **(SI Appendix, Fig. S3)**. Higher priority was given to potential targets bearing critical amino acid differences in these positions or possessing allosteric binding sites where selective drugs can be synthesized. Because of the intensive nature of structural studies, we have limited this category to only potential targets that yielded the highest composite score in the other listed categories (**Dataset S3**). If a gene scored ≥10 in the categories of RNAi Severity, Mammalian Essentiality, Assayability, and Druggability, they qualified for additional investigation for parasite selectivity. This cut-off afforded structural analysis for 65 essential genes.

Structural analysis was first performed by identifying binding pockets and key binding residues from data available in literature or on the Protein Data Bank (PDB)[29,30] for human orthologs of schistosome target proteins.

For sequence alignment, corresponding human and schistosome protein sequences were retrieved using UniProt[11] and WormBase Parasite[19], respectively. Sequences were aligned using Clustal[27,28]. Then, we looked for differences in key residues involved in substrate or drug binding interactions or the formation of binding pockets. For 3D homology modelling, we searched the PDB[29,30] for solved structures (x-ray crystallography, cryo-EM, etc.) of the human orthologs of potential parasite target proteins. Structures were used if they were of sufficient quality or involved binding of a drug included in inhibitor testing. Here, we define sufficient quality according to wwPDB validation reporting for each PDB dataset, where good quality structures have metrics (Rfree, Clashscore, Ramachanran outliers, Sidechain outliers, RSRZ outliers) that rank as 'better' relative to X-ray structures of similar resolution and bearing 'better' quality ligand structure fit. Additionally, we retrieved theoretical models of schistosome proteins available on AlphaFold. We have provided identifiers for both AlphaFold and PDB models used in these studies (**Dataset S3**). Homology modelling was conducted using ChimeraX[31] and Pymol[32], identifying residue overlap using the matchmaker and/or alignment function. In some instances, we also utilized LigPlot[33,34] to visualize binding pockets of human orthologs of schistosome target proteins to display important binding interactions.

We awarded the highest score (3) to potential targets whose active sites and allosteric binding pockets are well-defined, and there are unique residues in either of these respective features that could be exploited for the development of selective therapeutics. The next highest score (2) was given to candidates whose active sites were not well known or had <70% identity based on sequence alignment using Clustal[27,28]. A score of 1 was given to a target whose active site residues are known and conserved but only based on sequence alignment. The lowest score (0) was given to a target whose active site residues are known and conserved based on 3D structure and sequence alignment.

Biological outcomes

A final consideration to the amenability of a candidate target to drug discovery is whether on-target activity can be established. It is vital to reliably measure target-specific biological outcomes as a result of its modulation by a small molecule. Some biological processes are highly conserved in species and can be readily applied, such as protein degradation by the proteasome, which can be monitored with a western blot using antibodies that

recognize various ubiquitin linkages (DUBs, p97, proteasome, etc.). Additional examples include the maturation of lysosomes using an LC3B antibody (ULK2, ATG4B), or histone methylation (KMT2, KDM1A, etc.). Alternatively, other targets produce an essential metabolite whose abundance can be measured through robust techniques like LC-MS/MS (MAT2A, PGD, PLD2, etc.). Kinases, while heavily studied, may not be ideal in this manner because understanding the downstream outcomes of inhibition may require the conservation of a phosphorylation site or process. Because of this, antibodies detecting phosphorylated substrates might not be reliable for detection of on-target effects of inhibitors without additional studies to validate these tools. To be given final consideration, a potential drug target must have ideal characteristics in a majority of the above criteria, and a viable means to measure on-target pharmacologic action of a small molecule on its target. To establish whether a biological outcome could be measured following inhibition, we performed literature searches on known inhibitors of human orthologs of schistosome target proteins. If there was an established, robust method to measure target engagement and pharmacologic action, then we prioritized these genes for additional studies.

Identification of inhibitors for drug targets to test on adult schistosome parasites

Because each essential gene in our RNAi screen originated from a specific human ortholog included in our initial bioinformatic queries, we revisited this dataset to determine which orthologous human drug target prompted discovery of our drug target candidates in schistosomes. After this, we manually searched various databases (GeneCards, DrugBank, Google, Therapeutic Target Database, etc.)[1,6,35] and chemical vendors (Medchemexpress, Selleck Chem, etc.) for inhibitors that target these human proteins. In each instance, we consulted published literature to determine which inhibitors were worth testing. We pursued inhibitors for targets that displayed favorable characteristics in each of our *in silico* prioritization criteria, and whose actions could possibly be assessed for on-target activity. Preference was also given to inhibitors that were more drug-like in nature[21–25], had been validated in other systems and had sub-micromolar IC_{50} or EC_{50} values determined experimentally. Also, we pursued drugs that were in clinical trials, FDA-approved, or whose targets had published structures in complex with these inhibitors.

Evaluation of commercially available inhibitors

Selected compounds were evaluated *in vitro* for preliminary potency at a single concentration (10 μM in 0.1% DMSO). All selected compounds were obtained from commercial vendors (Medchemexpress, Selleck Chemicals, Sigma-Aldrich, ApexBio, Tocris, Santa Cruz Biotechnology, and Cayman Chemical Company) (**Dataset S4**) with a minimum compound purity of 98% to reinforce that effects observed on schistosome parasites were related to the activity of the desired compounds. Roughly 5 adult worm pairs were placed in 3mL Basch medium in a 12-well culture vessel, and incubated at 37°C, 5% CO_2 for the length of the experiment. Compound was administered at D0, D1, and D2, media and drug refreshed every 24 hr. Worms were then allowed to remain in culture until D5, where a final assessment of worm attachment to tissue culture substate was made by light microscopy. Observations on movement and morphology were also recorded. A negative control (0.1% DMSO) and positive control (PZQ; 10 μM in 0.1% DMSO) were included in each experiment, and each experiment was repeated in triplicate for validation. For compounds that caused complete detachment in replicate experiments, the treatment was expanded to three concentrations (10 μM, 5 μM, and 1 μM) following the same treatment schedule and controls as detailed above. If compounds still displayed potency at 1 μM, then a full dose-response titration (50 μM - 10 nM) of compound was performed to assess schistosomicidal potency. Each titration experiment was performed in triplicate according to the treatment regimen described above, using DMSO and PZQ as appropriate controls. Morphologies and motility were assessed by light microscopy, and dose-response curves were calculated using the primary readout of parasite attachment to tissue culture substrate in GraphPad Prism.

Western blot analysis

For drug treatment studies, 10 male adult worms (single or paired with females) were supplemented with either 0.1% DMSO or inhibitor (0.1% DMSO final). For all experiments assessing on-target effects of potential parasite p97 inhibitors, CB-5083 was used as a positive control (0.1% DMSO). After 48 hr, male parasites were separated from females using 0.25% tricaine in Basch Media 169, flash frozen in liquid nitrogen, and stored at -80°C until further processing. Male worm samples were homogenized with a pestle in 50 μL lysis buffer containing 2X sample buffer [0.471 M Tris (pH 6.7 with phosphoric acid), 20% glycerol, 5% SDS), protease inhibitor cocktail (Roche, cOmplete Mini, EDTA-free Tablets) and 10 mM DTT. The lysates were then sonicated on high for 5 min (30 sec on, 30 sec off) using a Bioruptor UCD-200. Lysates were centrifuged for 5 min at 10,000 g to remove

debris. Total protein was measured using the Detergent Compatible Bradford Assay (Pierce). 50 μg of protein samples denatured in SDS Sample buffer (95°C for 5 min) were separated on a Bio-Rad 4-20% TGX Stain-Free gel along with Precision Plus Protein Dual Color Standards (Bio-Rad) as a marker. Proteins were then transferred to a nitrocellulose membrane (Bio-Rad). The membrane was blocked in a 1:5 solution of Li-Cor Odyssey Blocking buffer in PBST for 1 hr before being immunoblotted overnight at 4°C with 1:500 K48-linkage Specific Polyubiquitin Antibody (Cell Signaling Technology, 4289S) and 0.01 μg/mL mouse anti-actin antibody (1:10,000) (Developmental Studies Hybridoma Bank, JLA20) diluted in a 1:5 solution of Li-Cor Odyssey Blocking buffer in PBST. The membrane was washed 3x in TBST and then incubated in 1:5 Li-Cor Odyssey Blocking buffer containing the secondary antibodies (1:10,000 Li-Cor, 925-68071, goat anti-rabbit IRDye 680 RD, and 1:20,000 Li-Cor, 925-32280, goat anti-mouse IgM IRDye 800CW) for 1 hr at RT. The blot was washed in TBST 3x before being imaged on a LiCor Odyssey Infrared Imager.

Purification of recombinant p97

For recombinant expression, full-length wildtype *Schistosoma mansoni* p97 (Smp_018240) or *Homo sapiens* p97 (P55072) were synthesized and cloned into pET28a(+) with an N-terminal 6x His tag by GenScript (Piscaaway, NJ). *E. coli* BL21 (DE3) containing the desired plasmid were grown in LB medium containing 50 μg/L kanamycin while shaking at 37 °C to an OD600 of 0.6-0.8. The temperature was reduced to 18°C and 0.8 mM isopropyl-beta-D-thiogalactopyranoside (IPTG) was added. The bacterial culture was harvested 16hr later by centrifugation. The resulting pellet was suspended in 60 mL lysis buffer/2L bacterial culture [20 mM Tris (pH 7.4), 300 mM NaCl, 5 mM MgCl2, 20 mM imidazole, 1% Triton X-100, 1 mg/mL lysozyme, 10% glycerol, 3 mM β-mercaptoethanol, 0.2 mM PMSF, and protease inhibitor tablet (Roche)]. The cells were incubated for 30min at 4°C, rocking, then subjected to subsequent lysis by dounce hom ogenization (10x) and sonication (3x 30s pulses, 65% amplitude, 3min rest on ice in between). The lysate was centrifuged at 40,000 x rpm for 35 min at 4°C. The resulting supernatant was incubated with Ni-NTA beads equilibrated to lysis buffer for 1 hr at 4°C before being loaded onto a gravity column. The bead bed was washed with wash buffer [20 mM Tris (pH 7.4), 500 mM NaCl, 5 mM MgCl2, 40 mM imidazole, 0.05% Triton X-100, 10% glycerol, 3 mM β-mercaptoethanol, 0.2 mM PMSF, 0.001 mg/mL Aprotinin, and 0.002 mg/mL Leupeptin], then eluted in the same buffer with 250 mM imidazole in

wash buffer. Fractions containing p97 were concentrated using Pierce Protein Concentrators (100k MWCO) and loaded onto a gel-filtration column (Superdex 200PG) and eluted using SEC buffer [20 mM Tris (pH 7.4), 180 mM NaCl, 5 mM MgCl2, and 10% glycerol] at 0.5 mL/min flow rate on a BioRad NGC Chromatography System. Fractions corresponding to an apparent molecular weight of 500 – 600 kDa were collected and analyzed by 4–20% SDS/PAGE to evaluate purity. Concentration was determined using a BSA standard. Fractions containing purified protein were aliquoted, snap frozen in liquid nitrogen, and stored at −80 °C until use.

Cryo-EM Sample Preparation

Prior to grid preparation, 20 µl of a freshly thawed aliquot of 1 mg/mL purified p97 [20 mM Tris (pH 7.4), 180 mM NaCl, 5 mM MgCl2, and 1 mM tris(2-carboxyethyl)phosphine (TCEP)] was incubated with 100 µM inhibitor (0.5% DMSO final) for 30 min at room temperature. For experiments with ATPγS, the protein was pre-incubated with 1 mM ATPγS (dissolved in H_2O) for 30 minutes at room temperature before making grids. 3 µL were applied to holey carbon grids (Quantifoil R1.2/1.3, 300 mesh copper) and plunge frozen using the Vitrobot Mark IV System (Thermo Fisher). Grids were glow-discharged using a PELCO easiGlow glow discharge apparatus (Ted Pella) for 80 s at 30 mA before use sample application. Grids were screened at UTSW Cryo Electron Microscopy Facility (CEMF) on a 200kV microscope and the best grid with optimal particle distribution was used for data collection.

Cryo-EM Data Acquisition and Processing

Apo *S. mansoni* p97 dataset was collected at the CEMF on a 300kV Titan Krios microscope equipped with BioQuantum energy filter and K3 detector (Gatan) at CEMF in non-CDS mode at 105 kX magnification with SerialEM[36] at ~15 e^-/pix/sec, with defocus range -0.9 to -2.2 µm and total dose of 50 $e^-/Å^2$ in 24 hr, which yielded 6,939 movies at pixel size of 0.83 Å. ATPγS- and CB-5083- bound data sets were collected on the same microscope in CDS mode at 105 kX magnification with SerialEM at ~9 e^-/pix/sec, with defocus range -0.9 to -2.2 µm, and -1.0 to -2.4 µm, respectively, and total dose of ~60 $e^-/Å^2$ in 24 hr. 5,730 and 4,579 movies were yielded, respectively. 739-bound sample was collected on a Titan Krios microscope equipped with a Selectris energy filter and Falcon 4i detector (Thermo Fisher Scientific) at CEMF at 165 kX magnification with SerialEM at ~9 e^-/pix/sec, with defocus range -0.9 to -2.2 µm and total dose of 60 $e^-/Å^2$ in 24 hr, which yielded 9,352 movies

at pixel size of 0.738 Å. 804-bound sample was collected at the Pacific Northwest Cryo-EM Center (PNCC) for 48 hr data collection on a Titan Krios microscope equipped with a Selectris energy filter and Falcon 4i detector at 165 kX magnification with EPU at ~9 e^-/pix/sec, with defocus range -0.9 to -2.2 μm and total dose of 50 $e^-/Å^2$, yielded a total of 17,748 movies at pixel size of 0.7296 Å.

All data processing was performed using the software cryoSPARC v4.2 (Apo, ATPγS- and CB5083- bound) or v4.5 (739- and 804-bound) using default parameters[37]. For K3 datasets, a ½ F-crop factor was applied during patch motion correction, followed by patch CTF estimation. For Falcon 4i datasets, eer upsampling factor of 1 was used during motion correction and CTF estimation in cryoSPARC Live.

The first dataset collected was the CB-5083-bound dataset, a subset of 135 micrographs was selected and blob picker was used to pick 18,949 particles, which went through two rounds of 2D classification to yield 8,843 particles that were used as input for Topaz training. Topaz was used to repick 16,463 particles from the same subset, which were used to generate three *ab initio* reconstructions with C6 symmetry. Topaz was then used to pick particles from the whole dataset of 4,174 micrographs, which yielded an initial 968,168 particles. After one round of 2D classification, 529,148 particles and the three *ab initio* models were used as input for heterogenous refinement with C6 symmetry. 75% of the particles were partitioned in a class that resembles p97. These particles were further submitted in *ab initio* reconstruction to obtain three new models and cleaned up with two rounds of heterogenous refinement with C6 symmetry. In the last heterogenous refinement, 88% of the particles, which includes 174,830 particles, resembled p97 and were submitted for final refinement using homogenous refinement with C6 symmetry applied, with global CTF refinement and local motion correction to obtain a 2.85 Å map. The map was sharpened by deepEMhancer[38], which was used for modeling.

For the Apo dataset, the CB-5083-bound map was used to generate templates which was used for template picking on the whole dataset to yield 1,949,076 initial particles. These were cleaned up for two rounds of 2D classification to yield 878,454 particles. After two rounds of heterogenous refinement with C6 symmetry (using three of the same CB-5083-bound map as input model), 85% of the particles, which includes 478,635 particles, resembled p97 in the last iteration and were submitted for final refinement using homogenous refinement with C6 symmetry

and global CTF refinement to obtain a 2.72 Å map. The map was also sharpened by deepEMhancer to obtain final map used for modeling.

Similar procedure was applied to the ATPγS-bound dataset. Template picking yielded 1,681,589 initial picks, which were cleaned up by two rounds of 2D classification. 1,074,975 particles were submitted to one round of heterogenous refinement with C6 symmetry. 62% of the particles, which includes 665,490 particles, were submitted to final refinement using homogenous refinement with C6 symmetry, global and local CTF refinements, to obtain a 2.2 Å map. DeepEMhancer sharpened map was used for modeling.

For compound 739-bound dataset, the same CB-5083-bound map was used to generate templates for Template picking in cryoSPARC Live, which yielded 2,812,039 particles. After two rounds of 2D classification, 373,213 particles were selected and used in two rounds of heterogenous refinement using two of the same CB-5083-bound map as initial references and C6 symmetry. 78% of the particles in the last heterogenous refinement job, which includes 101,826 particles, were submitted to final refinement using non-uniform refinement with C6 symmetry applied, with global and CTF refinements, to yield a 3.06 Å map. Unsharpened map was used for modeling.

Similar procedure was applied for compound 804-bound dataset. Template picking in cryoSPARC Live yielded 2,272,397 particles, which were cleaned up in one round of 2D classification to 966,087 particles. After two rounds of heterogeneous refinement, 95% of the particles in the last iteration, including 823,143 particles, were submitted to final refinement with homogenous refinement with C6 symmetry applied, and global and local CTF refinements, to yield a 2.76 Å map. DeepEMhancer sharpened map was used for modeling.

Cryo-EM model building, refinement and analysis

ModelAngelo[39] was used to generate an initial 3D-model for CB-5083-bound p97 in one chain. Errors were fixed manually in Coot[40]. The other chains were built based on the initial ModelAngelo model by manually fitting into the map in Chimera, followed by merging all chains in Coot. Modeling of the CB-5083 is kept consistent with previous published models (PDB: 7RLI, 6MCK) as the ligand density is ambiguous for the primary amide extension on the inhibitor. Model refinement was carried out by multiple cycles of real-space refinement in Phenix[41] and manual model building using Coot. The same initial model from ModelAngelo was used to build the Apo model, where the single chain was docked into the map in ChimeraX and undergone manual modeling

in Coot. The rest of the chains were built using molrep in CCP-EM[42]. Model refinement was carried out as described above. The ATPγS-bound model was built using the fully built Apo model with 6 chains as an initial model, which underwent model refinement using ISOLDE[43] in ChimeraX. ATPγS and Mg^{2+} were first modelled manually in coot and included in refinement in Phenix in later steps. For compound 739- and 804-bound models, the fully built Apo model was used and first refined against their respective maps in Phenix. Restraints for the compounds and its conjugation to a cysteine side chain were generated in JLigand[44] and used in Coot for manual building of the compounds into chain D. Other copies of the ligands were built in Coot using NCS ligand tool and refined individually to optimize map fitting and geometry. All model validation was performed and analyzed in PyMOL 2.2.3 or 3.1.3 where the final figures were generated[32]. Ligplot[33] was used to generate interaction figures.

Evaluation of p97's ATPase activity and compound potency

For manual ATP consumption assays, 1 μM p97 was incubated with 1% DMSO or inhibitor (1% DMSO final) in assay buffer [50 mM Tris (pH 7.5), 20 mM MgCl2, 1 mM EDTA, 0.5 mM tris(2-carboxyethyl)phosphine (TCEP)] for 5 minutes prior to the addition of 20 μM ATP in a 384-well plate for a total reaction volume of 10 μL, and a final concentration of 500nM enzyme and 10 μM ATP. The reaction was allowed to proceed for 50 minutes at room temperature before adding equivalent volume of Kinase-Glo Plus reagent (Promega), and a further incubation of 10 minutes in the dark. Luminescence was read using a BioTek Synergy 2 plate reader (model no. 3375752).

To determine IC_{50} values, compounds were assayed at a range of concentrations (100 μM, 50 μM, 10 μM, 5 μM, 1 μM, 500 nM, 100 nM, 50 nM, 10 nM, 5 nM, 1 nM, and 0 nM) in triplicate, maintaining a concentration of 1% DMSO. The percent of inhibition was calculated using CB-5083 as a control for 100% inhibition. IC_{50} values were calculated using GraphPad Prism.

High-throughput screening (HTS) assay

Kinase-Glo (Promega, part K1214) was purchased from Promega. Microtiter plates (384 wells) were purchased from Perkin Elmer. All chemicals included in the UTSW library were sourced from commercial sources [ChemDiv (150,000 compounds), ChemBridge (125,500), ComGenex (22,000), Prestwick Chemical, (1,100) and TimTec (500)] or UT Southwestern chemists (https://www.utsouthwestern.edu/research/core-facilities/high-

throughput-screening/libraries/). For initial screening, compounds were dispensed by Echo 555 robot for a final concentration of 10 μM (0.2% DMSO). Luminescence was read by EnVision multimode plate reader (Perkin-Elmer). Assay conditions were consistent between manual and high-throughput assays [10 μM ATP, 50 mM Tris (pH 7.5), 20 mM MgCl2, 1 mM EDTA, 0.5 mM tris(2-carboxyethyl)phosphine (TCEP)]. The plates were then incubated for 50 min at room temperature before addition of Kinase-Glo reagent and further incubation in the dark for 10 minutes before reading luminescence. CB-5083 was used as a control for 100% inhibition, 10 μM ATP for maximal signal, and DMSO for 0% inhibition. Raw values and values normalized to CB-5083 control were obtained to determine activity of compounds on the enzyme. Hit compounds were validated using a dose-response curve (19.8 μM, 9.92 μM, 4.96 μM, 3.72 μM, 2.48 μM, 1,24 μM, 0.619 μM, 0.342 μM, 0.256 μM, 0.171 μM, 0.0854 μM, 0.0427 μM, and 0 μM) in triplicate. Compounds were also subjected to a counter screen using a dose-response curve against the full-length human p97 enzyme to determine preliminary selectivity.

Mass spectrometry analysis of benzoxazole propiolamide covalent inhibitors

1mg/mL aliquots of recombinant *S. mansoni* p97 [20 mM Tris (pH 7.4), 180 mM NaCl, 5 mM MgCl2, and 1 mM tris(2-carboxyethyl)phosphine (TCEP)] were thawed to room temperature. Purified enzyme was then incubated with 100 μM inhibitor (242 and 243) (1% DMSO) or negative control (1% DMSO only) for 20 min prior to submission for intact mass spectrometry analysis. In-gel LC-MS/MS experiments were performed similarly on recombinant *S. mansoni* and *H. sapiens* p97. However, the reaction with 100 μM inhibitor (242 and 243) (1% DMSO) or negative control (1% DMSO only) was halted by the addition of 4X loading (1X final concentration). The samples were denatured in SDS sample buffer (95°C for 5min), then separated on a Bio-Rad 4-20% TGX Stain-Free gel along with Precision Plus Protein Dual Color Standards (Bio-Rad) as a marker. Following separation, bands were detected using GelCode™ Blue Safe Protein Stain (Cat# 24594). Peptides were excised from the gel corresponding to the molecular weight of p97, then submitted to UTSW Proteomics Core for in-gel trypsin digestion prior to extraction, desalting, and downstream LC-MS/MS analysis.

HepG2 cytotoxicity assay of benzoxazole propiolamide covalent inhibitors

HepG2 cells (ATCC) were plated at 2500 cells/well in 50 μL/well, dispersing cells using a 25g needle to break up clumps prior to plating. Plates were incubated for 24hr at 37C, 5% CO2. Following 24hr, 50 μL media

containing 0.6% DMSO was added to a set of wells on one plate. The plate and Cell Titer Glo 2.0 reagent were brought to RT. A T=0 read was taken by adding 40 µL reagent/well, rocking the plate at 100 rpm for 2 min, then reading luminescence ~10 min later. Serial 1:3 dilutions of compound at 333 x concentration were made in DMSO. These stocks were then diluted 167-fold into media to make a 2x concentrated stock containing 0.6% DMSO. 50 µL 2x concentrated compound was added to wells containing cells in 50 µL 24 hr after plating. Concentrations were tested in triplicate at following values: 30, 10, 3.3, 1.1, 0.37, 0.12, and 0.04 µM. Plates were incubated for 72 hr at 37C, 5% CO2. Following equilibration to room temperature, 40 µL undiluted reagent was added to each well, the plate rocked at 100 rpm for 2 min and then read for luminescent signal ~10 min later.

GI50 is the concentration that causes 50% growth inhibition. It corrects for the cell count at time zero; thus, GI50 is the concentration of test drug where 100 × (T - T0)/(C - T0) = 50. IC_{50} is the concentration that results in 50% growth inhibition relative to control and is calculated as the test drug concentration where 100 x (T/C) = 50. Values are plotted in excel and the 50% point determined by interpolation. Values were revisualized in Prism.

Chemical Synthesis Methods

General procedure 1 (Amidation by HATU)

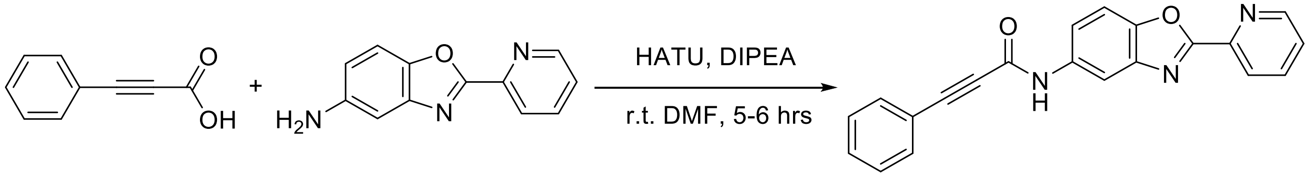

Experimental procedure (General Procedure 1, Amidation by HATU)

To a solution of phenylpropiolic acid (0.3 mmol, 1 equiv) in *N,N*-dimethylformamide (2.0 mL) was added amines (0.3 mmol, 1 equiv), HATU (125.5 mg, 0.33 mmol, 1.1 equiv) and *N,N*-diisopropylethylamine (104.5 µL, 0.6 mmol, 2 equiv). The mixture was sealed in a 20-mL scintillation vial and stirred at room temperature for 5 to 6 hours. The reaction crudes were poured onto ice to form off-white precipitates. The precipitates were collected by filtration and washed with water to afford off-white solid as products, yields are 23.7-99.6%.

General procedure 2 (Amidation by T_3P)

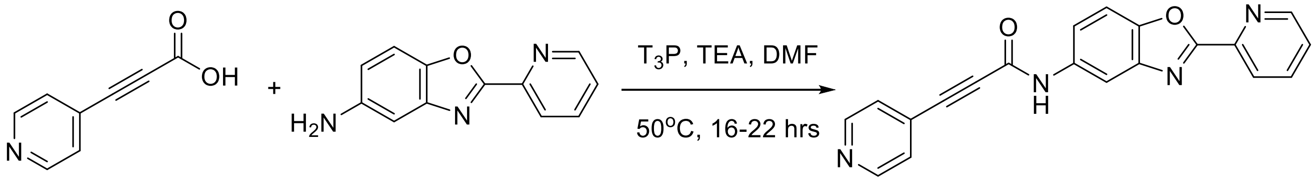

Experimental procedure (General Procedure 2, Amidation by T_3P)

To a solution of 3-(4-pyridyl)propiolic acid (0.1 mmol, 1 equiv) in *N,N*-dimethylformamide (0.8 mL) was added amines (0.1 mmol, 1 equiv), T_3P (propylphosphonic anhydride solution, ≥50 wt. % in ethyl acetate, 95.5 mg, 0.15 mmol, 1.5 equiv) and triethylamine (44.6 µL, 0.32 mmol, 3.2 equiv). The mixture was sealed in a 20-mL scintillation vial and stirred at room temperature to 50°C for about 20 hours. The reaction crudes were poured onto ice to form grey to brown precipitates. The precipitates were collected by filtration and washed with water and/or dichloromethane and hexane to afford off-white solid as products, yields are 32.1-68.5%.

General procedure 3 (Synthesis of Pyridinyl Benzoxazole Amines by PPA)

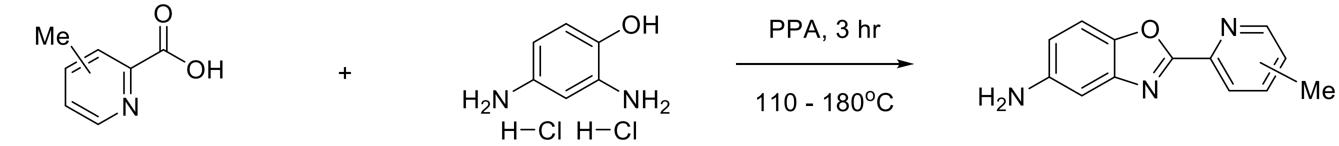

Methylpicolinic acid **2,4-diaminophenol dihydrochloride (Amidol)**

Experimental procedure (General Procedure 3, Synthesis of Pyridinyl Benzoxazole Amines by PPA)

To PPA (polyphosphoric acid, 1.0 g, 0.5 mL) at 110°C were added simultaneously 2,4-diaminophenol dihydrochloride (98.5 mg, 0.5 mmol, 1.0 equiv) and methylpicolinic acid (68.6 mg, 0.5 mmol, 1.0 equiv). The resulting mixture was then heated to 180°C for 3 hours. The mixture turned very gluey, hard to stir. It may or may not form bubbles at this moment. After cooling, ice chips were added to the crude and then sodium carbonate powder (about 1.2 g) was added in small portions. Lots of bubbles formed in the beginning. When no more bubbles formed, it turned to greenish-black suspension, and pH is about 11. The greenish-black suspension was extracted by dichloromethane. The combined organic layers were concentrated on vacuum to yield yellow crude solid. It was further purified by column chromatography or trituration. Yield 33.6-78.6%.

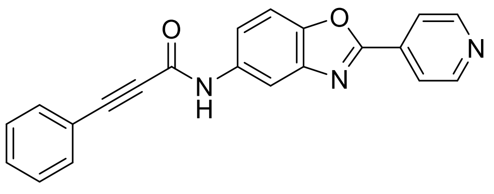

3-phenyl-*N*-(2-(pyridin-4-yl)benzo[*d*]oxazol-5-yl)propiolamide (**242**)

Experimental procedure

242 was synthesized by using phenylpropiolic acid (44.8 mg, 0.3 mmol, 1 equiv) and 2-pyridin-4-yl-benzooxazol-5-ylamine (61.2 mg, 0.3 mmol, 1 equiv) in General Procedure 1. The reaction was stirred at room temperature for 5 hours. The product was washed by water and 1 M NaOH as 70.0 mg brown solid. Yield 71.2%. ^{1}H NMR (400 MHz, Chloroform-*d*) δ 8.62 (d, J = 5.5 Hz, 2H), 8.01 (d, J = 2.1 Hz, 1H), 7.94 (d, J = 5.2 Hz, 2H), 7.59 (dd, J = 8.9, 2.1 Hz, 1H), 7.43 (d, J = 8.0 Hz, 3H), 7.28 (d, J = 7.2 Hz, 1H), 7.26 – 7.22 (m, 2H). ^{13}C NMR (101 MHz, Chloroform-*d*) δ 161.0, 151.8, 150.1, 147.5, 141.5, 135.6, 134.4, 132.4, 130.1, 128.4, 121.1, 119.9, 119.5, 111.7, 110.8, 85.9, 83.2. ESI-MS *m/z* = 340.1 ([M+H]$^{+}$), $C_{21}H_{13}N_{3}O_{2}$ requires 340.1

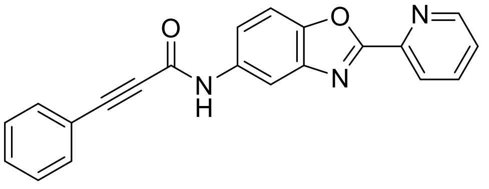

3-phenyl-*N*-(2-(pyridin-2-yl)benzo[*d*]oxazol-5-yl)propiolamide (**243**)

Experimental procedure

243 was synthesized by using phenylpropiolic acid (44.4 mg, 0.3 mmol, 1 equiv) and 2-pyridin-2-yl-benzooxazol-5-ylamine (61.5 mg, 0.3 mmol, 1 equiv) in General Procedure 1. The reaction was stirred at room temperature for 5 hours. The product was washed by water as 98.4 mg off-white solid. Yield 99.6%. ^{1}H NMR (400 MHz, Chloroform-*d*) δ 8.59 (d, J = 4.9 Hz, 1H), 8.11 (d, J = 7.9 Hz, 1H), 8.01 (s, 1H), 7.72 (t, J = 7.5 Hz, 1H), 7.54 (d, J = 8.8 Hz, 1H), 7.36 (d, J = 8.8 Hz, 1H), 7.33 – 7.25 (m, 3H), 7.19 (d, J = 7.4 Hz, 1H), 7.12 (t, J = 7.5 Hz, 2H). ^{13}C NMR (101 MHz, Chloroform-*d*) δ 162.0, 151.8, 149.9, 147.6, 145.2, 141.5, 137.3, 135.3, 132.3, 130.0, 128.3, 125.8, 123.5, 119.8, 119.2, 111.8, 110.9, 85.9, 83.3. ESI-MS *m/z* = 340.1 ([M+H]$^{+}$), $C_{21}H_{13}N_{3}O_{2}$ requires 340.1

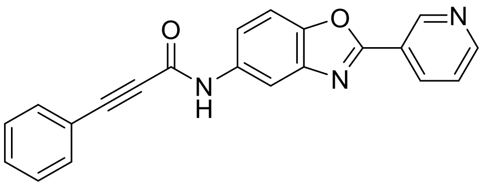

3-phenyl-*N*-(2-(pyridin-3-yl)benzo[*d*]oxazol-5-yl)propiolamide (**244**)

Experimental procedure

244 was synthesized by using phenylpropiolic acid (45.4 mg, 0.3 mmol, 1 equiv) and 2-(3-pyridinyl)-5-benzoxazolamine (63.1 mg, 0.3 mmol, 1 equiv) in General Procedure 1. The reaction was stirred at room temperature for 5 hours. The product was washed by water as 95.4 mg red-brown solid. Yield 94.1%. ^{1}H NMR (400 MHz, Chloroform-*d*) δ 9.36 (s, 1H), 8.69 (d, J = 4.9 Hz, 1H), 8.45 (d, J = 8.0 Hz, 1H), 7.98 (d, J = 2.1 Hz, 1H), 7.67 (dd, J = 8.9, 2.1 Hz, 1H), 7.56 – 7.51 (m, 2H), 7.45 (dd, J = 8.0, 4.9 Hz, 2H), 7.38 (dd, J = 8.0, 1.7 Hz, 1H), 7.35 – 7.29 (m, 2H). ^{13}C NMR (101 MHz, Chloroform-*d*) δ 161.1, 151.8, 151.5, 148.0, 147.4, 141.5, 135.4, 135.1, 132.4, 130.1, 128.4, 1241, 123.4, 119.9, 118.9, 111.4, 110.6, 85.8, 83.2. ESI-MS *m/z* = 340.1 ([M+H]$^{+}$), $C_{21}H_{13}N_{3}O_{2}$ requires 340.1

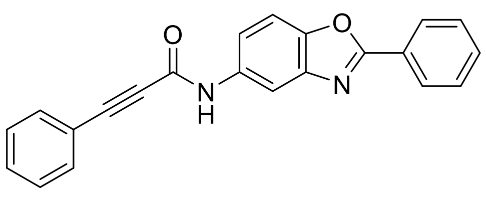

3-phenyl-*N*-(2-phenylbenzo[*d*]oxazol-5-yl)propiolamide (**245**)

Experimental procedure

245 was synthesized by using phenylpropiolic acid (44.9 mg, 0.3 mmol, 1 equiv) and 2-phenyl-benzooxazol-5-ylamine (63.6 mg, 0.3 mmol, 1 equiv) in General Procedure 1. The reaction was stirred at room temperature for 5 hours. The product was washed by water as 103.7 mg off-white solid. Quantitative yield. ^{1}H NMR (400 MHz, Chloroform-*d*) δ 8.18 (dd, J = 7.8, 2.0 Hz, 2H), 7.91 (d, J = 2.1 Hz, 1H), 7.67 (dd, J = 8.8, 2.1 Hz, 1H), 7.54 – 7.47 (m, 6H), 7.39 – 7.30 (m, 3H). ^{13}C NMR (101 MHz, Chloroform-*d*) δ 164.0, 151.8, 147.4, 141.7, 135.0, 132.4, 131.8, 130.1, 128.9, 128.4, 127.4, 126.4, 120.0, 118.3, 111.1, 110.5, 85.8, 83.3. ESI-MS *m/z* = 339.1 ([M+H]$^+$), $C_{22}H_{14}N_2O_2$ requires 339.1

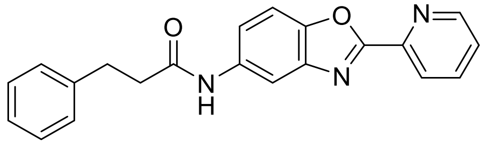

3-phenyl-*N*-(2-(pyridin-2-yl)benzo[*d*]oxazol-5-yl)propanamide (**326**)

Experimental procedure

326 was synthesized by hydrogenation of **243**. A hydrogenation balloon was attached to a sealed vial with alkyne (17.2 mg, 0.05 mmol, 1 equiv) and 10% palladium on carbon (10.6 mg, 0.01 mmol, 0.2 equiv) in a mixed solvent of 1 mL ethyl acetate and 1 mL of methanol. The mixture was stirred at room temperature for 6 hours. The catalyst was filtered off by celite and solvents were removed. The product was washed by hexane to obtain as 16.8 mg off-white solid. Yield 96.5%. ^{1}H NMR (400 MHz, Chloroform-*d*) δ 8.78 (d, J = 4.7 Hz, 1H), 8.31 (d, J = 7.9 Hz, 1H), 7.92 – 7.83 (m, 2H), 7.52 (d, J = 8.7 Hz, 1H), 7.46 – 7.41 (m, 2H), 7.31 – 7.18 (m, 5H), 3.06 (t, J = 7.5 Hz, 2H), 2.68 (t, J = 7.7 Hz, 2H). ^{13}C NMR (101 MHz, Chloroform-*d*) δ 171.7, 161.9, 150.0, 145.4, 140.7, 137.4, 135.8, 128.4, 128.2, 126.1, 125.5, 123.5, 119.4, 118.9, 111.6, 110.8, 110.5, 38.8, 31.6. ESI-MS *m/z* = 344.2 ([M+H$^+$]), $C_{21}H_{17}N_3O_2$ requires 344.1

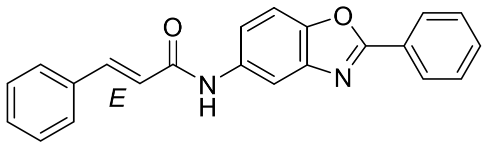

N-(2-phenylbenzo[*d*]oxazol-5-yl)cinnamamide (**135**)

Experimental procedure

135 was synthesized by using *trans*-cinnamic acid (8.3 mg, 0.05 mmol, 1 equiv) and 2-phenyl-benzooxazol-5-ylamine (9.9 mg, 0.05 mmol, 1 equiv) in General Procedure 1. The reaction was stirred at room temperature overnight. The product was washed by dichloromethane and hexane as 13.6 mg off-white solid. Yield 84.8%. ^{1}H NMR (400 MHz, Chloroform-*d*) δ 8.14 (dd, J = 7.7, 2.1 Hz, 2H), 7.87 (d, J = 2.2 Hz, 1H), 7.78 (dd, J = 8.7, 2.2 Hz, 1H), 7.67 (d, J = 15.6 Hz, 1H), 7.51 – 7.41 (m, 6H), 7.33 – 7.26 (m, 3H), 6.62 (d, J = 15.6 Hz, 1H). ^{13}C NMR (151 MHz, Chloroform-*d*) δ 164.8, 163.9, 147.3, 141.8, 141.7, 135.8, 134.8, 131.8, 129.7, 128.9, 128.7, 127.8, 127.5, 126.6, 120.9, 118.4, 110.7, 110.5. ESI-MS *m/z* = 341.1 ([M+H$^+$]), $C_{22}H_{16}N_2O_2$ requires 341.1

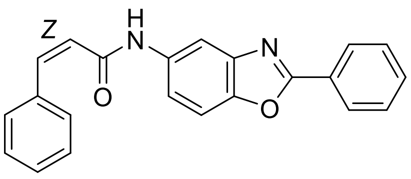

(*Z*)-3-phenyl-*N*-(2-phenylbenzo[*d*]oxazol-5-yl)acrylamide (**324**)

Experimental procedure

324 was synthesized by hydrogenation of **245**. A hydrogenation balloon was attached to a sealed vial with alkyne (15.7 mg, 0.05 mmol, 1 equiv) and Lindlar catalyst (2.1 mg, 0.001 mmol, 0.02 equiv) in 0.25 mL of methanol. The mixture was stirred at room temperature for 20 hours until the complete disappearance of starting material. The catalyst was filtered off by celite and solvents were removed. The product was washed by hexane to obtain 14.6 mg off-white solid. Yield 92.4%. ^{1}H NMR (400 MHz, Chloroform-*d*) δ 8.13 (dd, J = 7.8, 2.0 Hz, 2H), 7.69 (d, J = 2.1 Hz, 1H), 7.53 – 7.41 (m, 8H), 7.33 – 7.22 (m, 2H), 6.83 (d, J = 12.5 Hz, 1H), 6.09 (d, J = 12.5 Hz, 1H). ^{13}C NMR (101 MHz, Chloroform-*d*) δ 165.6, 163.9, 149.9, 147.4, 141.8, 137.9, 134.8, 131.8, 129.1, 128.9, 128.8, 1284, 127.5, 126.6, 124.1, 118.5, 111.1, 110.4. ESI-MS *m/z* = 341.1 ([M+H^{+}]), $C_{22}H_{16}N_2O_2$ requires 341.1

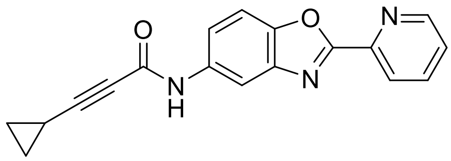

3-cyclopropyl-*N*-(2-(pyridin-2-yl)benzo[*d*]oxazol-5-yl)propiolamide (**732**)

Experimental procedure

732 was synthesized by using 3-cyclopropylprop-2-ynoic acid (7.5 mg, 0.05 mmol, 1 equiv) and 2-pyridin-2-yl-benzooxazol-5-ylamine (10.6 mg, 0.05 mmol, 1 equiv) in General Procedure 1. The reaction was stirred at room temperature overnight. The product was washed by water, followed by trituration in dichloromethane and hexane to afford 15.0 mg light-brown solid. Yield 90.8%. ^{1}H NMR (600 MHz, Chloroform-*d*) δ 8.74 (d, J = 4.1 Hz, 1H), 8.28 (d, J = 7.9 Hz, 1H), 7.93 (d, J = 2.2 Hz, 1H), 7.87 (td, J = 7.8, 1.8 Hz, 1H), 7.62 (dd, J = 8.8, 2.2 Hz, 1H), 7.53 (d, J = 8.9 Hz, 1H), 7.44 (ddd, J = 7.6, 4.8, 1.2 Hz, 1H), 1.35 (tt, J = 8.2, 5.1 Hz, 1H), 0.93 – 0.83 (m, 4H). ^{13}C NMR (151 MHz, Chloroform-*d*) δ 162.3, 151.9, 150.2, 147.8, 145.6, 141.7, 137.5, 135.4, 126.0, 123.6, 119.3, 111.7, 111.2, 92.4, 71.1, 9.0, -0.6. ESI-MS *m/z* = 304.1 ([M+H^{+}]), $C_{18}H_{13}N_3O_2$ requires 304.1

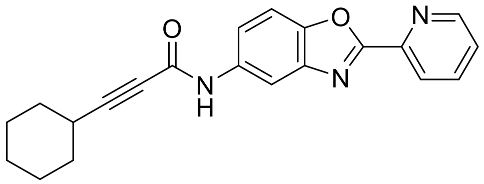

3-cyclohexyl-*N*-(2-(pyridin-2-yl)benzo[*d*]oxazol-5-yl)propiolamide (**733**)

Experimental procedure

733 was synthesized by using 3-cyclohexylpropiolic acid (9.1 mg, 0.05 mmol, 1 equiv) and 2-pyridin-2-yl-benzooxazol-5-ylamine (11.1 mg, 0.05 mmol, 1 equiv) in General Procedure 1. The reaction was stirred at room temperature overnight. The product was washed by water, followed by trituration in dichloromethane and hexane to afford 12.6 mg off-white solid. Yield 69.4%. ^{1}H NMR (600 MHz, Chloroform-*d*) δ 8.72 (d, J = 4.7 Hz, 1H), 8.26 (d, J = 7.8 Hz, 1H), 7.94 (t, J = 2.8 Hz, 1H), 7.87 (ddd, J = 7.7, 5.2, 2.5 Hz, 1H), 7.62 (dt, J = 8.9, 2.3 Hz, 1H), 7.52 (dd, J = 9.1, 3.8 Hz, 1H), 7.43 (s, 1H), 2.47 (td, J = 9.5, 4.7 Hz, 1H), 1.81 (d, J = 12.9 Hz, 2H), 1.68 (p, J = 5.3, 4.5 Hz, 2H), 1.51 – 1.40 (m, 3H), 1.27 (s, br, 3H). ^{13}C NMR (151 MHz, Chloroform-*d*) δ 162.2, 152.1, 150.2, 147.8, 145.5, 141.7, 137.5, 135.4, 126.0, 123.6, 119.4, 111.8, 111.2, 92.5, 75.8, 31.7, 29.0, 25.6, 24.8. ESI-MS *m/z* = 346.2 ([M+H^{+}]), $C_{21}H_{19}N_3O_2$ requires 346.2

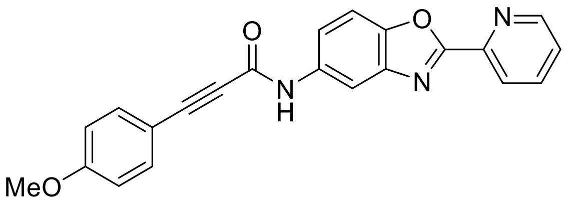

3-(4-methoxyphenyl)-*N*-(2-(pyridin-2-yl)benzo[*d*]oxazol-5-yl)propiolamide (**734**)

Experimental procedure
734 was synthesized by using 3-(4-methoxyphenyl)propiolic acid (9.7 mg, 0.05 mmol, 1 equiv) and 2-pyridin-2-yl-benzooxazol-5-ylamine (10.2 mg, 0.05 mmol, 1 equiv) in General Procedure 1. The reaction was stirred at room temperature overnight. The product was eluted by 2.5% methanol in dichloromethane from silica gel column to afford 11.1 mg light-brown solid. Yield 62.2%. ^{1}H NMR (600 MHz, Chloroform-*d*) δ 8.81 (dt, J = 4.7, 1.3 Hz, 1H), 8.35 (d, J = 7.9 Hz, 1H), 8.10 (d, J = 2.0 Hz, 1H), 8.01 (s, 1H), 7.90 (td, J = 7.8, 1.8 Hz, 1H), 7.62 (dd, J = 8.8, 2.0 Hz, 1H), 7.59 (d, J = 8.7 Hz, 1H), 7.49 (d, J = 8.8 Hz, 2H), 7.46 (ddd, J = 7.6, 4.8, 1.2 Hz, 1H), 6.87 (d, J = 8.8 Hz, 2H), 3.82 (s, 3H). ^{13}C NMR (151 MHz, Chloroform-*d*) δ 162.4, 161.2, 151.5, 150.3, 148.1, 145.7, 142.2, 137.2, 134.8, 134.4, 125.7, 123.6, 119.1, 114.3, 112.1, 111.6, 111.3, 86.8, 82.6, 55.4. ESI-MS *m/z* = 370.2 ([M+H^{+}]), $C_{22}H_{15}N_{3}O_{3}$ requires 370.1

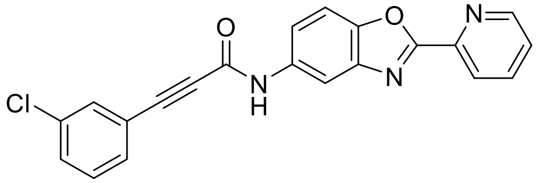

3-(3-chlorophenyl)-*N*-(2-(pyridin-2-yl)benzo[*d*]oxazol-5-yl)propiolamide (**735**)

Experimental procedure
735 was synthesized by using 3-(3-chlorophenyl)propiolic acid (10.4 mg, 0.05 mmol, 1 equiv) and 2-pyridin-2-yl-benzooxazol-5-ylamine (10.0 mg, 0.05 mmol, 1 equiv) in General Procedure 1. The reaction was stirred at room temperature overnight. The product was eluted by 5% methanol in dichloromethane from silica gel column, followed by trituration in dichloromethane and hexane to afford 4.2 mg off-white solid. Yield 23.7%. ^{1}H NMR (600 MHz, Chloroform-*d*) δ 8.82 (d, J = 4.8 Hz, 1H), 8.36 (d, J = 7.9 Hz, 1H), 8.09 (d, J = 1.9 Hz, 1H), 7.91 (td, J = 7.8, 1.7 Hz, 1H), 7.63 (d, J = 8.7 Hz, 1H), 7.61 (dd, J = 8.8, 1.9 Hz, 1H), 7.55 (t, J = 1.8 Hz, 1H), 7.48 – 7.45 (m, 3H), 7.33 (t, J = 7.9 Hz, 1H). ^{13}C NMR (151 MHz, Chloroform-*d*) δ 162.5, 161.5, 152.3, 150.3, 148.3, 145.8, 142.3, 137.2, 134.4, 132.3, 130.7, 129.8, 125.8, 123.6, 119.1, 112.2, 112.1, 111.4, 84.3, 84.0. ESI-MS *m/z* = 374.1 ([M+H^{+}]), $C_{21}H_{12}ClN_{3}O_{2}$ requires 374.1

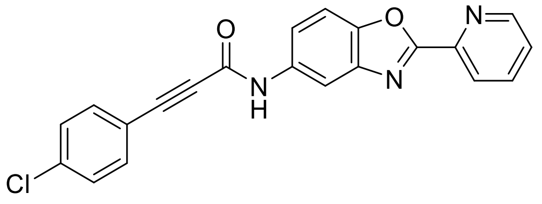

3-(4-chlorophenyl)-*N*-(2-(pyridin-2-yl)benzo[*d*]oxazol-5-yl)propiolamide (**736**)

Experimental procedure
736 was synthesized by using 3-(4-chlorophenyl)propiolic acid (11.2 mg, 0.05 mmol, 1 equiv) and 2-pyridin-2-yl-benzooxazol-5-ylamine (10.6 mg, 0.05 mmol, 1 equiv) in General Procedure 1. The reaction was stirred at room temperature overnight. The product was eluted by 5% methanol in dichloromethane from silica gel column, followed by trituration in dichloromethane and hexane to afford 10.8 mg off-white solid. Yield 57.6%. ^{1}H NMR

(600 MHz, Chloroform-*d*) δ 8.80 (d, J = 5.1 Hz, 1H), 8.34 (d, J = 7.9 Hz, 1H), 8.08 (d, J = 2.1 Hz, 1H), 7.90 (t, J = 7.7 Hz, 1H), 7.62 (dd, J = 8.9, 1.9 Hz, 1H), 7.59 (d, J = 8.7 Hz, 1H), 7.45 – 7.42 (m, 3H), 7.33 – 7.30 (m, 2H). ^{13}C NMR (151 MHz, Chloroform-*d*) δ 162.4, 161.7, 152.1, 150.3, 148.2, 145.7, 142.1, 137.2, 134.5, 133.7, 129.0, 125.8, 123.6, 119.1, 112.2, 111.8, 111.3, 84.8, 84.0. ESI-MS *m/z* = 374.1 ([M+H$^+$]), $C_{21}H_{12}ClN_3O_2$ requires 374.1

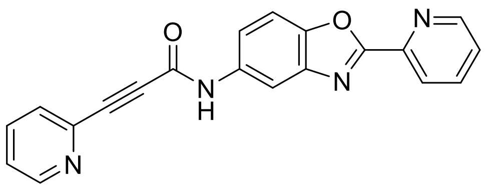

3-(pyridin-2-yl)-*N*-(2-(pyridin-2-yl)benzo[*d*]oxazol-5-yl)propiolamide (**737**)

Experimental procedure

737 was synthesized by using 3-(pyridin-2-yl)propiolic acid (3.8 mg, 0.025 mmol, 1 equiv) and 2-pyridin-2-yl-benzooxazol-5-ylamine (4.5 mg, 0.025 mmol, 1 equiv) in General Procedure 2. The reaction was stirred at room temperature for 20 hours. The product was eluted by 5% methanol in dichloromethane from silica gel column to afford 3.5 mg light yellow solid. Yield 48.3%. ^{1}H NMR (600 MHz, Chloroform-*d*) δ 8.82 (dt, J = 4.7, 1.3 Hz, 1H), 8.66 (dt, J = 4.9, 1.3 Hz, 1H), 8.37 (d, J = 7.9 Hz, 1H), 8.24 (d, J = 5.1 Hz, 1H), 8.13 (d, J = 2.1 Hz, 1H), 7.91 (td, J = 7.7, 1.8 Hz, 1H), 7.77 (td, J = 7.7, 1.8 Hz, 1H), 7.65 (dt, J = 7.8, 1.1 Hz, 1H), 7.63 (d, J = 8.7 Hz, 1H), 7.59 (dd, J = 8.8, 2.1 Hz, 1H), 7.47 (ddd, J = 7.6, 4.7, 1.2 Hz, 1H), 7.39 (ddd, J = 7.7, 4.9, 1.2 Hz, 1H). ^{13}C NMR (151 MHz, Chloroform-*d*) δ 162.5, 150.4, 150.3, 150.3, 148.3, 145.8, 142.2, 140.6, 137.2, 136.7, 134.5, 128.7, 125.7, 124.7, 123.6, 118.9, 112.1, 111.4, 83.6, 82.1. ESI-MS *m/z* = 340.1 ([M+H$^+$]), $C_{20}H_{12}N_4O_2$ requires 340.1

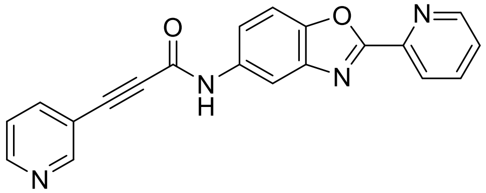

N-(2-(pyridin-2-yl)benzo[*d*]oxazol-5-yl)-3-(pyridin-3-yl)propiolamide (**738**)

Experimental procedure

738 was synthesized by using 3-(pyridin-3-yl)propiolic acid (15.4 mg, 0.1 mmol, 1 equiv) and 2-pyridin-2-yl-benzooxazol-5-ylamine (24.0 mg, 0.1 mmol, 1 equiv) in General Procedure 2. The reaction was stirred at room temperature for 26 hours. The product was washed by water, dichloromethane and hexane to afford 24.4 mg off-white solid. Yield 68.5%. ^{1}H NMR (600 MHz, Methanol-*d$_4$*) δ 8.81 (d, J = 2.0 Hz, 1H), 8.77 (dt, J = 4.8, 1.3 Hz, 1H), 8.64 (dd, J = 5.0, 1.6 Hz, 1H), 8.39 (d, J = 7.9 Hz, 1H), 8.27 (d, J = 2.0 Hz, 1H), 8.08 (dt, J = 7.9, 1.9 Hz, 1H), 8.06 (dd, J = 7.8, 1.7 Hz, 1H), 7.71 (d, J = 8.8 Hz, 1H), 7.66 (dd, J = 8.8, 2.1 Hz, 1H), 7.62 (ddd, J = 7.6, 4.8, 1.2 Hz, 1H), 7.52 (dd, J = 7.9, 5.0 Hz, 1H). ^{13}C NMR (151 MHz, Methanol-*d$_4$*) δ 163.5, 153.3, 152.5, 151.1, 150.9, 149.1, 146.3, 142.9, 141.4, 139.1, 136.8, 127.4, 125.0, 124.8, 120.3, 119.2, 112.9, 112.2, 87.2, 82.6. ESI-MS *m/z* = 340.1 ([M+H$^+$]), $C_{20}H_{12}N_4O_2$ requires 340.1

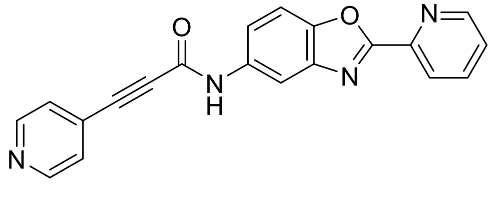

N-(2-(pyridin-2-yl)benzo[*d*]oxazol-5-yl)-3-(pyridin-4-yl)propiolamide (**739**)

Experimental procedure

739 was synthesized by using 3-(4-pyridyl)propiolic acid (17.7 mg, 0.1 mmol, 1 equiv) and 2-pyridin-2-yl-benzooxazol-5-ylamine (23.5 mg, 0.1 mmol, 1 equiv) in General Procedure 2. The reaction was stirred at room temperature for 26 hours. The product was eluted by 5% methanol in dichloromethane from silica gel column to afford 25.5 mg light yellow solid. Yield 67.3%. ^{1}H NMR (400 MHz, Chloroform-*d*) δ 8.81 (d, J = 4.9 Hz, 1H), 8.65 (d, J = 5.2 Hz, 2H), 8.35 (d, J = 7.9 Hz, 1H), 8.07 (d, J = 2.1 Hz, 1H), 7.91 (td, J = 7.8, 1.7 Hz, 1H), 7.71 – 7.64 (m, 1H), 7.62 (d, J = 8.8 Hz, 1H), 7.47 (ddd, J = 7.5, 4.8, 1.1 Hz, 1H), 7.44 (d, J = 6.0 Hz, 2H). ^{13}C NMR (151 MHz, Chloroform-*d*) δ 162.2, 150.5, 150.1, 149.5, 147.9, 145.3, 141.6, 137.4, 135.1, 129.0, 126.1, 125.9, 123.5, 119.2, 111.7, 111.2, 87.0, 81.5. ESI-MS *m/z* = 340.1 ([M+H$^+$]), $C_{20}H_{12}N_4O_2$ requires 340.1

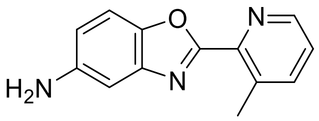

2-(3-methylpyridin-2-yl)benzo[*d*]oxazol-5-amine (**804-i**)

Experimental procedure

804-i was synthesized by using 3-methylpicolinic acid (68.8 mg, 0.5 mmol, 1 equiv) in General Procedure 3. The product was eluted by 5% methanol in dichloromethane from silica gel column to afford 51.4 mg brown solid. Yield 45.5%. ^{1}H NMR (400 MHz, Chloroform-*d*) δ 8.66 (dd, J = 5.1, 1.5 Hz, 1H), 7.69 (ddd, J = 7.8, 1.7, 0.8 Hz, 1H), 7.45 (d, J = 8.6 Hz, 1H), 7.33 (dd, J = 7.8, 4.6 Hz, 1H), 7.11 (d, J = 2.3 Hz, 1H), 6.77 (dd, J = 8.6, 2.3 Hz, 1H), 2.85 (s, 3H). ^{13}C NMR (151 MHz, Chloroform-*d*) δ 161.7, 147.3, 144.3, 144.3, 143.7, 142.8, 139.8, 135.0, 124.6, 114.8, 111.1, 105.3, 21.0. ESI-MS *m/z* = 226.1 ([M+H$^+$]), $C_{13}H_{11}N_3O$ requires 226.1

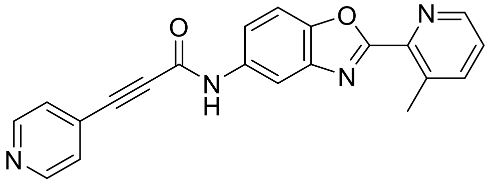

N-(2-(3-methylpyridin-2-yl)benzo[*d*]oxazol-5-yl)-3-(pyridin-4-yl)propiolamide (**804**)

Experimental procedure

804 was synthesized by using 3-(4-pyridyl)propiolic acid (8.1 mg, 0.05 mmol, 1 equiv) and 2-(3-methylpyridin-2-yl)benzo[*d*]oxazol-5-amine (**804-i**, 12.2 mg, 0.05 mmol, 1 equiv) in General Procedure 2. The reaction was stirred at 50°C for 26 hours. The product was eluted by 5% methanol in dichloromethane from silica gel column to afford 11.0 mg light yellow solid. Yield 57.3%. ^{1}H NMR (600 MHz, Methanol-*d$_4$*) δ 8.63 – 8.61 (m, 2H), 8.58 (dd, J = 4.7, 1.6 Hz, 1H), 8.25 (d, J = 2.0 Hz, 1H), 7.84 (dt, J = 7.8, 1.2 Hz, 1H), 7.66 (d, J = 8.8 Hz, 1H), 7.62 (dd, J = 8.8, 2.1 Hz, 1H), 7.60 – 7.57 (m, 2H), 7.47 (dd, J = 7.8, 4.7 Hz, 1H), 2.83 (s, 3H). ^{13}C NMR (151 MHz, Methanol-*d$_4$*) δ 163.1, 151.9, 150.3, 148.3, 147.9, 144.5, 142.8, 141.5, 136.8, 136.2, 130.5, 127.4, 126.5, 120.1, 113.1, 111.8, 87.9, 82.4, 21.1. ESI-MS *m/z* = 355.2 ([M+H$^+$]), $C_{21}H_{14}N_4O_2$ requires 355.1

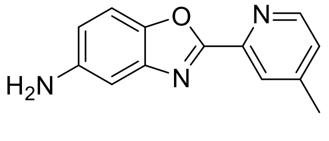

2-(4-methylpyridin-2-yl)benzo[*d*]oxazol-5-amine (**805-i**)

Experimental procedure

805-i was synthesized by using 4-methylpicolinic acid (140.6 mg, 1.0 mmol, 1 equiv) in General Procedure 3. The product was eluted by 5% methanol in dichloromethane from silica gel column to afford 181.6 mg yellow solid. Yield 78.6%. ^{1}H NMR (400 MHz, Chloroform-*d*) δ 8.61 (d, J = 5.0 Hz, 1H), 8.12 (s, 1H), 7.39 (d, J = 8.7 Hz, 1H), 7.20 (d, J = 5.0 Hz, 1H), 7.06 (d, J = 2.3 Hz, 1H), 6.74 (dd, J = 8.7, 2.3 Hz, 1H), 2.42 (s, 3H). ^{13}C NMR

(151 MHz, Chloroform-*d*) δ 161.5, 149.4, 148.0, 145.4, 144.4, 144.0, 142.3, 125.8, 123.6, 114.4, 110.7, 104.5, 20.6. ESI-MS *m/z* = 226.1 ([M+H$^+$]), $C_{13}H_{11}N_3O$ requires 226.1

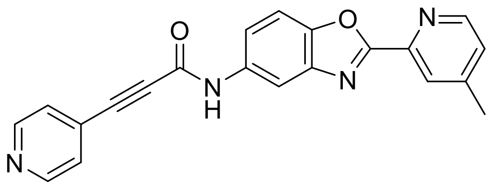

N-(2-(4-methylpyridin-2-yl)benzo[*d*]oxazol-5-yl)-3-(pyridin-4-yl)propiolamide (**805**)

Experimental procedure

805 was synthesized by using 3-(4-pyridyl)propiolic acid (7.5 mg, 0.05 mmol, 1 equiv) and 2-(4-methylpyridin-2-yl)benzo[*d*]oxazol-5-amine (**805-i**, 10.9 mg, 0.05 mmol, 1 equiv) in General Procedure 2. The reaction was stirred at 50°C for 22 hours. The product was eluted by 5% methanol in dichloromethane from silica gel column to afford 5.5 mg off-white solid. Yield 32.1%. ^{1}H NMR (600 MHz, Methanol-d_4) δ 8.65 – 8.63 (m, 2H), 8.59 (d, J = 5.0 Hz, 1H), 8.25 (d, J = 2.1 Hz, 1H), 8.22 (d, J = 1.6 Hz, 1H), 7.69 (d, J = 8.8 Hz, 1H), 7.64 (dd, J = 8.8, 2.1 Hz, 1H), 7.62 – 7.59 (m, 2H), 7.43 (dd, J = 5.2, 1.9 Hz, 1H), 2.52 (s, 3H). ^{13}C NMR (151 MHz, Methanol-d_4) δ 163.5, 152.0, 150.9, 150.6, 150.4, 149.1, 146.0, 142.8, 136.6, 130.5, 128.2, 127.5, 125.5, 120.2, 112.8, 112.1, 87.9, 82.5, 21.2. ESI-MS *m/z* = 355.2 ([M+H$^+$]), $C_{21}H_{14}N_4O_2$ requires 355.1

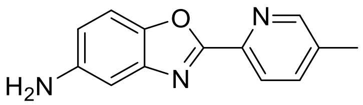

2-(5-methylpyridin-2-yl)benzo[*d*]oxazol-5-amine (**806-i**)

Experimental procedure

806-i was synthesized by using 5-methylpicolinic acid (137.5 mg, 1.0 mmol, 1 equiv) in General Procedure 3. The product was eluted by 5% methanol in dichloromethane from silica gel column to afford 124.4 mg yellow solid. Yield 55.1%. ^{1}H NMR (400 MHz, Chloroform-*d*) δ 8.51 (s, 1H), 8.10 (d, J = 8.1 Hz, 1H), 7.63 (d, J = 7.7 Hz, 1H), 7.36 (dd, J = 8.7, 1.5 Hz, 1H), 7.04 (s, 1H), 6.76 (dt, J = 8.6, 2.2 Hz, 1H), 2.36 (s, 3H). ^{13}C NMR (151 MHz, Chloroform-*d*) δ 161.8, 150.4, 144.7, 144.0, 143.0, 142.3, 137.5, 135.8, 122.7, 114.7, 111.0, 104.9, 18.4. ESI-MS *m/z* = 226.1 ([M+H$^+$]), $C_{13}H_{11}N_3O$ requires 226.1

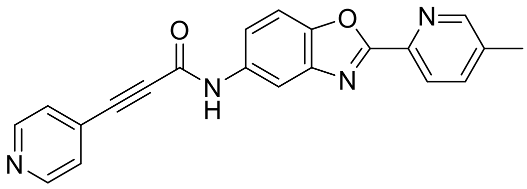

N-(2-(5-methylpyridin-2-yl)benzo[*d*]oxazol-5-yl)-3-(pyridin-4-yl)propiolamide (**806**)

Experimental procedure

806 was synthesized by using 3-(4-pyridyl)propiolic acid (8.0 mg, 0.05 mmol, 1 equiv) and 2-(5-methylpyridin-2-yl)benzo[*d*]oxazol-5-amine (**806-i**, 10.4 mg, 0.05 mmol, 1 equiv) in General Procedure 2. The reaction was stirred at 50°C for 22 hours. The product was eluted by 5% methanol in dichloromethane from silica gel column to afford 6.8 mg light yellow solid. Yield 41.6%. ^{1}H NMR (600 MHz, Methanol-d_4) δ 8.64 – 8.61 (m, 2H), 8.58 (d, J = 2.3 Hz, 1H), 8.24 (d, J = 8.0 Hz, 1H), 8.21 (d, J = 2.0 Hz, 1H), 7.83 (dd, J = 8.0, 3.0 Hz, 1H), 7.66 (d, J = 8.8 Hz, 1H), 7.62 (dd, J = 8.8, 2.1 Hz, 1H), 7.60 – 7.58 (m, 2H), 2.46 (s, 3H). ^{13}C NMR (151 MHz, Methanol-d_4) δ 163.5, 151.9, 151.3, 150.4, 148.9, 143.5, 142.7, 139.2, 138.1, 136.5, 130.5, 127.4, 124.3, 120.0, 112.7, 112.0, 87.9, 82.4, 18.7. ESI-MS *m/z* = 355.2 ([M+H$^+$]), $C_{21}H_{14}N_4O_2$ requires 355.1

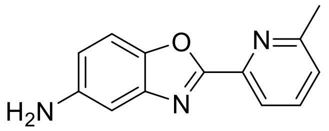

2-(6-methylpyridin-2-yl)benzo[*d*]oxazol-5-amine (**807-i**)

Experimental procedure
807-i was synthesized by using 6-methylpicolinic acid (138.8 mg, 1.0 mmol, 1 equiv) in General Procedure 3. The product was eluted by 5% methanol in dichloromethane from silica gel column to afford 76.5 mg orange solid. Yield 33.6%. ^{1}H NMR (400 MHz, Chloroform-*d*) δ 8.11 (d, J = 7.8 Hz, 1H), 7.74 (t, J = 7.8 Hz, 1H), 7.42 (d, J = 8.6 Hz, 1H), 7.28 (d, J = 7.7 Hz, 1H), 7.08 (d, J = 2.3 Hz, 1H), 6.75 (dd, J = 8.6, 2.3 Hz, 1H), 2.71 (s, 3H). ^{13}C NMR (151 MHz, Chloroform-*d*) δ 162.0, 159.3, 145.6, 145.0, 144.1, 142.9, 137.1, 125.1, 120.5, 114.7, 111.3, 105.2, 24.7. ESI-MS *m/z* = 226.1 ([M+H$^+$]), $C_{13}H_{11}N_3O$ requires 226.1

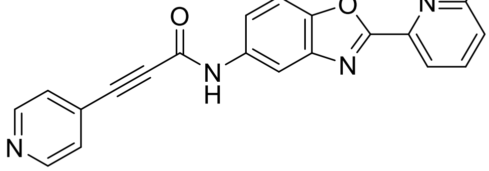

N-(2-(6-methylpyridin-2-yl)benzo[*d*]oxazol-5-yl)-3-(pyridin-4-yl)propiolamide (**807**)

Experimental procedure
807 was synthesized by using 3-(4-pyridyl)propiolic acid (7.1 mg, 0.05 mmol, 1 equiv) and 2-(6-methylpyridin-2-yl)benzo[*d*]oxazol-5-amine (**807-i**, 12.2 mg, 0.05 mmol, 1 equiv) in General Procedure 2. The reaction was stirred at 50°C for 26 hours. The product was eluted by 5% methanol in dichloromethane from silica gel column to afford 7.3 mg light yellow solid. Yield 42.7%. ^{1}H NMR (600 MHz, Methanol-*d*$_4$) δ 8.66 – 8.61 (m, 2H), 8.23 (d, J = 1.9 Hz, 1H), 8.16 (d, J = 7.8 Hz, 1H), 7.90 (t, J = 7.8 Hz, 1H), 7.69 (d, J = 8.8 Hz, 1H), 7.64 (dd, J = 8.9, 2.0 Hz, 1H), 7.62 – 7.58 (m, 2H), 7.45 (d, J = 7.8 Hz, 1H), 2.68 (s, 3H). ^{13}C NMR (151 MHz, Methanol-*d*$_4$) δ 163.5, 160.6, 151.8, 150.2, 148.9, 145.5, 142.6, 138.7, 136.3, 130.4, 127.3, 127.0, 121.8, 120.0, 112.7, 111.9, 87.8, 82.3, 24.3. ESI-MS *m/z* = 355.2 ([M+H$^+$]), $C_{21}H_{14}N_4O_2$ requires 355.1

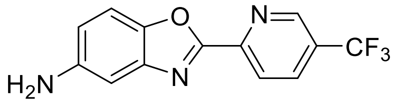

2-(5-(trifluoromethyl)pyridin-2-yl)benzo[*d*]oxazol-5-amine (**808-i**)

Experimental procedure
808-i was synthesized by using 5-(trifluoromethyl)-2-pyridinecarboxylic acid (98.9 mg, 0.5 mmol, 1 equiv) in General Procedure 3. The product was purified by trituration in dichloromethane and hexane to afford 68.2 mg yellow solid. Yield 47.2%. ^{1}H NMR (400 MHz, Chloroform-*d*) δ 9.04 (d, J = 1.2 Hz, 1H), 8.44 (d, J = 8.3 Hz, 1H), 8.12 (dd, J = 8.3, 2.3 Hz, 1H), 7.46 (d, J = 8.7 Hz, 1H), 7.10 (d, J = 2.3 Hz, 1H), 6.82 (dd, J = 8.6, 2.3 Hz, 1H). ^{13}C NMR (151 MHz, Chloroform-*d*) δ 160.5, 149.1, 147.1 (q, J = 4.1 Hz), 145.2, 144.6, 142.8, 134.4 (dd, J = 6.8, 3.3 Hz), 127.7 (d, J = 33.3 Hz), 125.7 (q, J = 493.8 Hz), 122.8, 115.8, 111.5, 105.2. ESI-MS *m/z* = 280.1 ([M+H$^+$]), $C_{13}H_8F_3N_3O$ requires 280.1

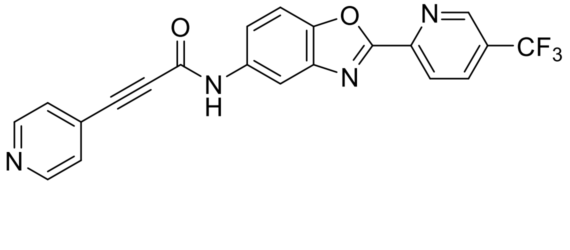

3-(pyridin-4-yl)-*N*-(2-(5-(trifluoromethyl)pyridin-2-yl)benzo[*d*]oxazol-5-yl)propiolamide (**808**)

Experimental procedure
808 was synthesized by using 3-(4-pyridyl)propiolic acid (9.6 mg, 0.05 mmol, 1 equiv) and 2-(5-(trifluoromethyl)pyridin-2-yl)benzo[*d*]oxazol-5-amine (**808-i**, 15.1 mg, 0.05 mmol, 1 equiv) in General Procedure 2. The reaction was stirred at 50°C for 22 hours. The product was eluted by 5% methanol in dichloromethane from silica gel column to afford 9.9 mg light yellow solid. Yield 44.8%. ^{1}H NMR (600 MHz, Methanol-d_4) δ 9.03 (d, J = 2.3 Hz, 1H), 8.65 – 8.61 (m, 2H), 8.51 (d, J = 8.2 Hz, 1H), 8.34 – 8.23 (m, 2H), 7.71 – 7.65 (m, 2H), 7.59 (q, J = 2.4 Hz, 2H). ^{13}C NMR (151 MHz, Methanol-d_4) δ 160.3, 150.9, 149.3, 148.4, 148.1, 146.8 (d, J = 3.6 Hz), 141.6, 135.7, 135.1 (d, J = 3.6 Hz), 129.4, 128.0 (dd, J = 65.5, 32.5 Hz), 126.4, 123.4, 123.2 (q, J = 272.5 Hz), 119.8, 116.7, 111.2, 86.8, 81.4. ESI-MS *m/z* = 409.2 ([M+H$^+$]), $C_{21}H_{11}F_3N_4O_2$ requires 409.1

Confirmation of benzoxazole propiolamide analog and intermediate compound synthesis were done using NMR spectroscopy and can be found in **Supporting Information - Benzoxazole propiolamide inhibitor analog chemical synthesis NMR**.

References

1. Li, Y. H. *et al.* Therapeutic target database update 2018: enriched resource for facilitating bench-to-clinic research of targeted therapeutics. *Nucleic Acids Res.* **46**, D1121–D1127 (2018).
2. Cannon, M. *et al.* DGIdb 5.0: rebuilding the drug–gene interaction database for precision medicine and drug discovery platforms. *Nucleic Acids Res.* **52**, D1227–D1235 (2024).
3. Freshour, S. L. *et al.* Integration of the Drug–Gene Interaction Database (DGIdb 4.0) with open crowdsource efforts. *Nucleic Acids Res.* **49**, D1144 (2020).
4. Wagner, A. H. *et al.* DGIdb 2.0: mining clinically relevant drug–gene interactions. *Nucleic Acids Res.* **44**, D1036–D1044 (2016).
5. Gaulton, A. *et al.* ChEMBL: a large-scale bioactivity database for drug discovery. *Nucleic Acids Res.* **40**, D1100 (2011).
6. Law, V. *et al.* DrugBank 4.0: shedding new light on drug metabolism. *Nucleic Acids Res.* **42**, D1091–D1097 (2014).
7. Finan, C. *et al.* The druggable genome and support for target identification and validation in drug development. *Sci. Transl. Med.* **9**, eaag1166 (2017).
8. Andrade, L. F. *et al.* Eukaryotic Protein Kinases (ePKs) of the Helminth Parasite Schistosoma mansoni. *BMC Genomics* **12**, 215 (2011).
9. Hirst, N. L., Nebel, J.-C., Lawton, S. P. & Walker, A. J. Deep phosphoproteome analysis of Schistosoma mansoni leads development of a kinomic array that highlights sex-biased differences in adult worm protein phosphorylation. *PLoS Negl. Trop. Dis.* **14**, e0008115 (2020).
10. Harrison, P. W. *et al.* Ensembl 2024. *Nucleic Acids Res.* **52**, D891–D899 (2024).
11. The UniProt Consortium. UniProt: the Universal Protein Knowledgebase in 2025. *Nucleic Acids Res.* gkae1010 (2024) doi:10.1093/nar/gkae1010.
12. Altschul, S. F., Gish, W., Miller, W., Myers, E. W. & Lipman, D. J. Basic local alignment search tool. *J. Mol. Biol.* **215**, 403–410 (1990).
13. Johnson, M. *et al.* NCBI BLAST: a better web interface. *Nucleic Acids Res.* **36**, W5-9 (2008).

14. McGinnis, S. & Madden, T. L. BLAST: at the core of a powerful and diverse set of sequence analysis tools. *Nucleic Acids Res.* **32**, W20-25 (2004).

15. Wang, J. *et al.* Large-scale RNAi screening uncovers therapeutic targets in the parasite Schistosoma mansoni. *Science* **369**, 1649–1653 (2020).

16. Ashburner, M. *et al.* Gene Ontology: tool for the unification of biology. *Nat. Genet.* **25**, 25–29 (2000).

17. The Gene Ontology Consortium *et al.* The Gene Ontology knowledgebase in 2023. *Genetics* **224**, iyad031 (2023).

18. Cioli, D., Pica-Mattoccia, L., Basso, A. & Guidi, A. Schistosomiasis control: praziquantel forever? *Mol. Biochem. Parasitol.* **195**, 23–29 (2014).

19. Howe, K. L., Bolt, B. J., Shafie, M., Kersey, P. & Berriman, M. WormBase ParaSite − a comprehensive resource for helminth genomics. *Mol. Biochem. Parasitol.* **215**, 2–10 (2017).

20. Chen, W.-H., Lu, G., Chen, X., Zhao, X.-M. & Bork, P. OGEE v2: an update of the online gene essentiality database with special focus on differentially essential genes in human cancer cell lines. *Nucleic Acids Res.* **45**, D940–D944 (2017).

21. Bickerton, G. R., Paolini, G. V., Besnard, J., Muresan, S. & Hopkins, A. L. Quantifying the chemical beauty of drugs. *Nat. Chem.* **4**, 90–98 (2012).

22. Johnson, T. W., Gallego, R. A. & Edwards, M. P. Lipophilic Efficiency as an Important Metric in Drug Design. *J. Med. Chem.* **61**, 6401–6420 (2018).

23. Leeson, P. D. & Springthorpe, B. The influence of drug-like concepts on decision-making in medicinal chemistry. *Nat. Rev. Drug Discov.* **6**, 881–890 (2007).

24. Lipinski, C. A. Drug-like properties and the causes of poor solubility and poor permeability. *J. Pharmacol. Toxicol. Methods* **44**, 235–249 (2000).

25. Lipinski, C. A., Lombardo, F., Dominy, B. W. & Feeney, P. J. Experimental and computational approaches to estimate solubility and permeability in drug discovery and development settings1. *Adv. Drug Deliv. Rev.* **46**, 3–26 (2001).

26. Jumper, J. *et al.* Highly accurate protein structure prediction with AlphaFold. *Nature* **596**, 583–589 (2021).

27. Sievers, F. *et al.* Fast, scalable generation of high-quality protein multiple sequence alignments using Clustal Omega. *Mol. Syst. Biol.* **7**, 539 (2011).

28. Sievers, F. & Higgins, D. G. Clustal Omega for making accurate alignments of many protein sequences. *Protein Sci.* **27**, 135–145 (2018).

29. Berman, H., Henrick, K. & Nakamura, H. Announcing the worldwide Protein Data Bank. *Nat. Struct. Mol. Biol.* **10**, 980–980 (2003).

30. Berman, H. M. *et al.* The Protein Data Bank. *Nucleic Acids Res.* **28**, 235–242 (2000).

31. Meng, E. C. *et al.* UCSF ChimeraX: Tools for structure building and analysis. *Protein Sci.* **32**, e4792 (2023).

32. The PyMOL Molecular Graphics System, Version 3.0 Schrödinger, LLC.

33. Laskowski, R. A. & Swindells, M. B. LigPlot+: Multiple Ligand–Protein Interaction Diagrams for Drug Discovery. *J. Chem. Inf. Model.* **51**, 2778–2786 (2011).

34. Wallace, A. C., Laskowski, R. A. & Thornton, J. M. LIGPLOT: a program to generate schematic diagrams of protein-ligand interactions. *Protein Eng.* **8**, 127–134 (1995).

35. Stelzer, G. *et al.* The GeneCards Suite: From Gene Data Mining to Disease Genome Sequence Analyses. *Curr. Protoc. Bioinforma.* **54**, 1.30.1-1.30.33 (2016).

36. Mastronarde, D. N. Automated electron microscope tomography using robust prediction of specimen movements. *J. Struct. Biol.* **152**, 36–51 (2005).

37. Punjani, A., Rubinstein, J. L., Fleet, D. J. & Brubaker, M. A. cryoSPARC: algorithms for rapid unsupervised cryo-EM structure determination. *Nat. Methods* **14**, 290–296 (2017).

38. Sanchez-Garcia, R. *et al.* DeepEMhancer: a deep learning solution for cryo-EM volume post-processing. *Commun. Biol.* **4**, 1–8 (2021).

39. Jamali, K. *et al.* Automated model building and protein identification in cryo-EM maps. *Nature* **628**, 450–457 (2024).

40. New tools for ligand refinement and validation in Coot and CCP4. *CoLab* https://colab.ws/articles/10.1107%2Fs0108767318096101.

41. Adams, P. D. *et al.* PHENIX: a comprehensive Python-based system for macromolecular structure solution. *Acta Crystallogr. D Biol. Crystallogr.* **66**, 213–221 (2010).

42. Burnley, T., Palmer, C. M. & Winn, M. Recent developments in the CCP-EM software suite. *Acta Crystallogr. Sect. Struct. Biol.* **73**, 469–477 (2017).

43. Croll, T. I. ISOLDE: a physically realistic environment for model building into low-resolution electron-density maps. *Acta Crystallogr. Sect. Struct. Biol.* **74**, 519–530 (2018).

44. Lebedev, A. A. *et al.* JLigand: a graphical tool for the CCP4 template-restraint library. *Acta Crystallogr. D Biol. Crystallogr.* **68**, 431–440 (2012).

Supplementary Tables

Supplementary Table 1. ***In silico*** **target priority score categories**

**RNAi Severity Score (RSS)**	
Modest phenotype, not seen until D25	0
Intermediate Onset (D16-D25), modest phenotype	1
Intermediate Onset (D16-D25), more than one phenotype	2
Rapid Onset (D7-D15), more than one phenotype	3
**Essentiality Score (ES)**	
Mouse Essentiality Score (Mouse ES)	
Essential in mice	0
No information available for mice	1
Essential, not embryonic lethal	2
Non-essential in mice	3
Human Essentiality Score (Human ES)	
Essential in human cells	0
No information available	1
Essential in some human cell lines	2
Non-essential in human cell lines	3
**Assayability Score (AS)**	
No clear path to recombinant purification or assay development	0
Purification and assay possible, will require extensive optimization	1
Purification and assay possible, will require some optimization	2
Estabilished purification schemes and assay available using commercial reagents	3
**Druggability Score (DS)**	
Weakly related to DrugBank/TTD target or distinct predicted biochemical activity	0
Similar to DrugBank/TTD target, no inhibitors described	1
Similar to DrugBank/TTD target, inhibitors described, but non-drug-like	2
Similar to DrugBank/TTD target, one or more drug-like compound	3
**Parasite Selectivity Score (PSS) *If applicable**	
Active site conserved based on 3D structure and alignment	0
Active site residues known, conserved based on sequence alignment	1
No active site information/structure available, < 70% identifty by sequence alignment	2
Active site residues known, unique based on structure and alignment	3
**Total Target Priority Score = RSS + ES + AS + DS + PSS**	

Supplementary Table 1. ***In silico*** **target priority score categories**

Scoring criteria rubric for the prioritization of essential schistosome genes identified by RNAi studies. There are five categories: RNAi Severity Score (RSS), Essentiality Score (ES) (Mouse and Human), Assayability Score (AS), Druggability Score (DS), and Parasite Selectivity Score (PSS). Targets were scored on a scale of ideal (3) to unideal (0) characteristics in each category. *Target genes were only assessed for parasite selectivity scoring through sequence alignments and 3D modelling if they had favorable scores in each of the other categories (≥10).

Supplementary Table 2. Cryo-EM data collection and model statistics.

	Apo Sm p97	Sm p97-ATPγS	Sm p97-CB-5083	Sm p97-739	Sm p97-804
**Data collection and processing**					
Magnification	105 kX	105 kX	105 kX	165 kX	165 kX
Voltage (kV)	300	300	300	300	300
Electron exposure (e^-/Å^2)	50	60	62	60	50
Defocus range (μm)	-0.9 to -2.2	-0.9 to -2.2	-1.0 to -2.4	-0.9 to -2.2	-0.9 to -2.2
Pixel size (Å)	0.84	0.83	0.83	0.738	0.7296
Symmetry imposed	C6	C6	C6	C6	C6
Initial particle images (no.)	1,949,076	1,681,589	968,168	2,812,039	2,272,397
Final particle images (no.)	478,635	665,490	174,830	101,602	823,143
Map resolution (Å)	2.72	2.2	2.85	3.07	2.76
FSC threshold	0.143	0.143	0.143	0.143	0.143
**Refinement**					
Initial model used	CB5083-structure	Apo structure	Model Angelo	Apo structure	Apo structure
Model composition					
Non-H atoms	24930	25626	25461	24665	24627
Protein residues	3183	3234	3230	3133	3127
Ligand	0	MG: 12<br>AGS: 12	JDP: 6	I73: 6	I80: 6
R.m.s. deviations					
Bond lengths (Å)	0.004	0.003	0.004	0.006	0.003
Bond angles (°)	0.516	0.465	0.928	0.606	0.494
CC (volume/mask)	0.82/0.82	0.83/0.82	0.78/0.78	0.82/0.83	0.79/0.79
CC for ligands	-	0.84	0.77	0.78	0.53
**Validation**					
MolProbity score	1.26	1.12	1.35	1.51	1.38
Clashscore	3.10	3.33	5.11	5.08	5.33
Poor rotamers (%)	0.04	0.00	0.00	0.00	0.00
Ramachandran plot					
Favored (%)	97.12	98.14	97.60	96.42	97.52
Allowed (%)	2.88	1.86	2.40	3.58	2.48
Disallowed (%)	0.00	0.00	0.00	0.00	0.00
Protein residues included in the model	A: 192-428, 434-546, 555-581, 593-708, 722-758<br>B: 193-427, 434-546, 555-581, 593-709, 722-758<br>C: 192-427, 434-546, 555-581, 593-709, 722-758<br>D: 193-428, 433-546, 555-581, 593-709, 722-758	A/B/C/D/E/F: 196-427, 434-547, 554-582, 593-709, 722-768	A: 193-427, 434-550, 554-582, 593-711, 720-758<br>B: 193-427, 434-550, 554-582, 593-710, 720-758<br>C: 193-427, 435-550, 554-582, 592-709, 720-758<br>D: 193-427, 434-550, 554-582, 592-709, 720-758	A: 194-426, 435-546, 555-580, 595-706, 722-758<br>B: 194-425, 435-550, 554-580, 595-707, 722-758<br>C: 194-427, 434-546, 554-581, 594-707, 722-758<br>D: 193-426, 436-546, 554-581, 594-707, 722-758	A: 192-424, 436-546, 555-581, 595-706, 722-758<br>B: 193-424, 436-546, 554-581, 595-706, 722-758<br>C: 192-424, 436-546, 554-582, 595-706, 722-758<br>D: 193-424, 436-546, 554-581, 595-706, 722-758

	E: 193-427, 434-546, 555-581, 594-709, 722-758<br>F: 191-425, 434-546, 555-581, 593-709, 722-758		E: 193-427, 434-550, 554-582, 592-708, 720-758<br>F: 193-427, 434-550, 554-582, 593-713, 720-758	E: 194-426, 436-546, 554-579, 595-707, 722-758<br>F: 193-425, 435-546, 554-580, 595-707, 722-758	E: 193-424, 435-546, 554-581, 594-706, 722-758<br>F: 191-424, 435-546, 554-581, 595-706, 722-758
PDB/EMDB code	9P00/EMD-71062	9P01/EMD-71063	9OX9/EMD-70961	9P02/EMD-71064	9P07/EMD-71066

Supplementary Table 3. p97 high-throughput screen pipeline

Category	Parameter	Description
Assay	Type of assay	*In vitro* ATP consumption (Kinase-Glo)
	Target	*S. mansoni* vcp/p97 (Smp_018240)
	Primary measurement	Luminescent signal compared to DMSO or known inhibitor (CB-5083) control to indicate inhibition of p97's ATPase activity
	Key reagents	Full-length (1-804aa) 6xHis *S. mansoni* p97, Promega Kinase-Glo, PerkinElmer 384-well plates
	Assay protocol	Described in Methods section
	Additional comments	None
Library	Library size	~350,000 compounds; 1 compound/well
	Library composition	Synthetic small molecules and partially purified natural product fractions
	Source	ChemDiv (150,000 compounds), ChemBridge (125,500), ComGenex (22,000), Prestwick Chemical (1,100), TimTec (500), UT Southwestern Chemistry in-house collection (2,500), Dr. John Macmillan (UC Santa Cruz) natural product collection (7,100 partially purified fractions)
	Additional Comments	
Screen	Format	384-well plates
	Concentration(s) tested	10 µM compound concentration; 0.2% DMSO
	Plate controls	Negative control (0.2% DMSO); Positive control (10 µM CB-5083); Maximal signal (10 µM ATP)
	Assay validation / Quality Control	Average plate Z'value = 0.673
	Correction factors	None
	Normalization	Normalized to internal inhibitor control (CB-5083)
	Additional comments	Screened at UTSW Medical Center HTS Facility
Post-HTS Analysis	Hit criteria	>25% enzyme activity inhibition
	Hit rate	0.2% (655 of 350,000) in initial screen. Each hit was re-assayed in quadruplicate using an 8-point dose-response curve (0.00614-15 µM)
	Additional assay(s)	Counter-screened each hit vs. purified recombinant full-length (1-806aa) *H. sapiens* p97 to determine preliminary selectivity using an 8-point dose-response curve (0.00614-15 µM) in quadruplicate
	Confirmation of hit purity and structure	
	Additional comments	Analysis performed at UTSW Medical Center by HTS Facility. Dr. Joseph Ready (UTSW Chemistry) assisted with removal of PAINS and promiscuous compounds.

Supplementary Figures

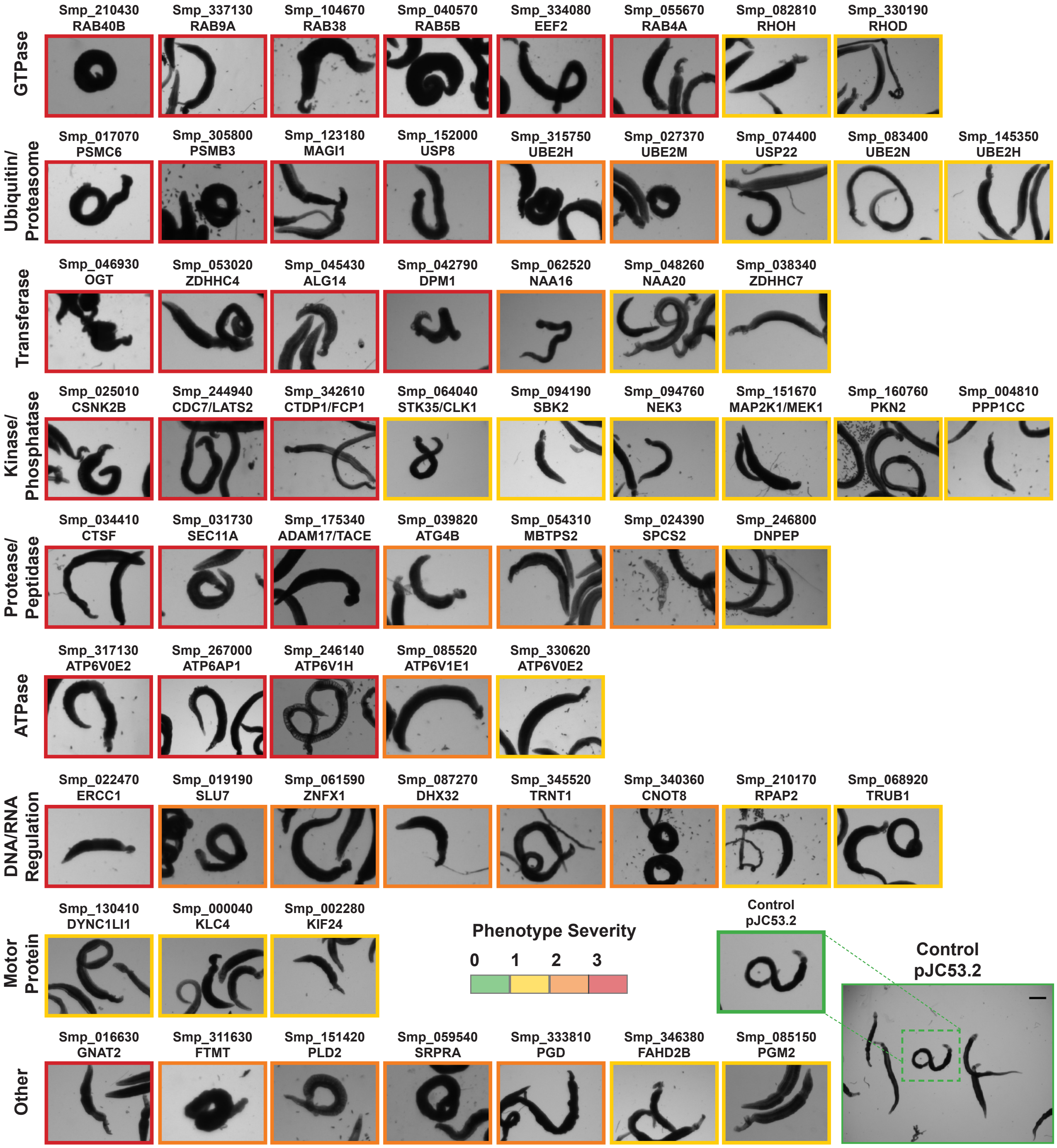

Supplemental Figure 1. Treatment of adult parasites with dsRNA targeting potential druggable genes

Light microscopy images of adult parasites treated with control dsRNA (pJC53.2) and dsRNAs targeting potential druggable genes within *S. mansoni* that bear homology to human drug targets. Targets were arranged according to order found in Figure 1c of enzymatic activity classification and phenotype severity. Scale bar, 1,000 µm.

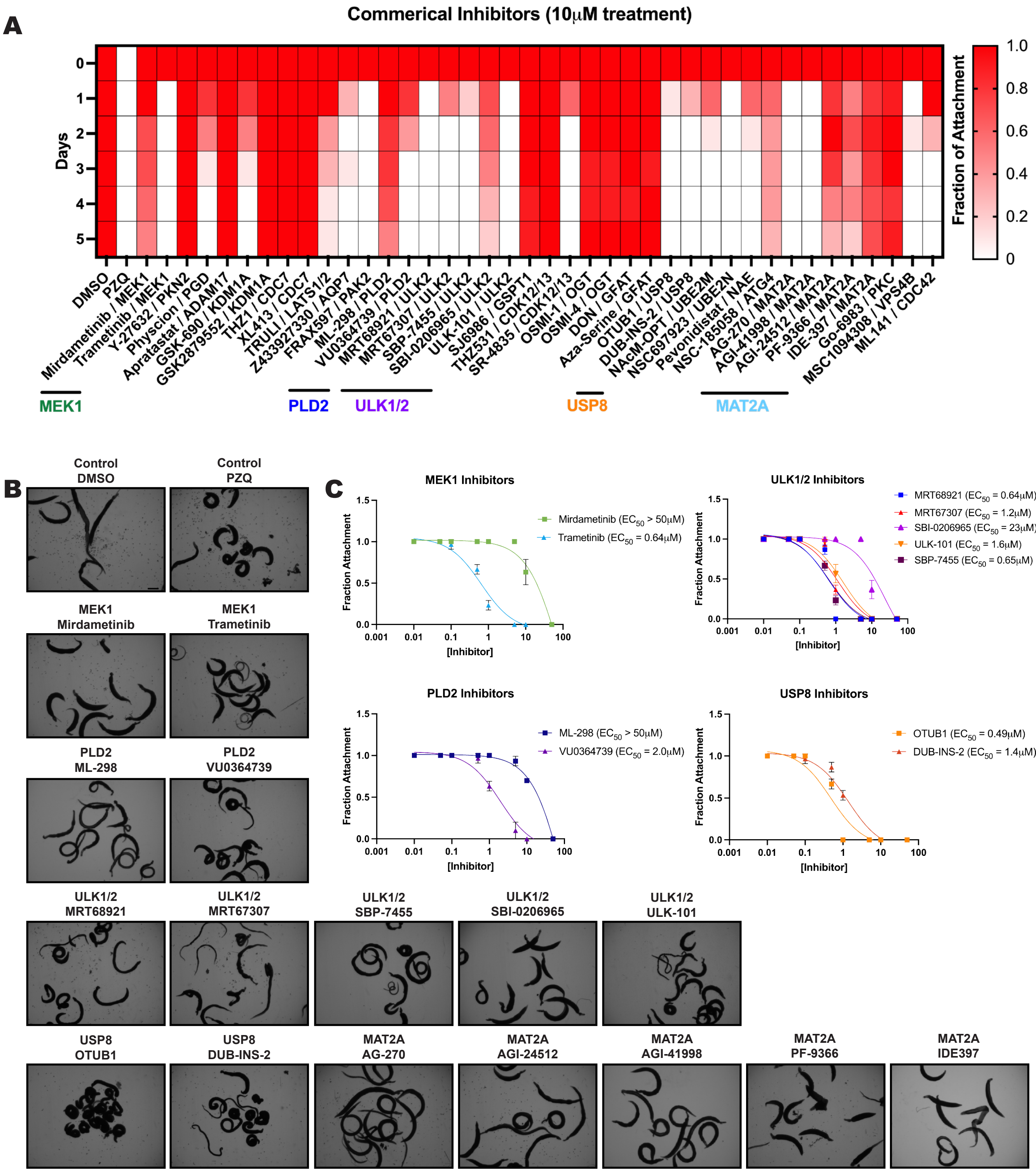

Supplemental Figure 2. Treatment of worms with human drug target inhibitors

(A) Heat map showing time course following treatment of adult worms at 10 μM with commercially available inhibitors targeting human orthologs of essential genes identified in RNAi experiments (DMSO; negative control, PZQ; positive control). The fraction of a population of 10 adult worms attached to the culture plate is quantified (dark red; 1 - complete attachment of entire population, white; 0 - no attachment of any worms in population).

Worms were treated with inhibitor for 72 hours, replacing drug and media every 24 hr, then monitored until the end of the experiment on day 5. **(B)** Light microscopy images of worms treated with DMSO control or reported inhibitors of human orthologs of essential schistosome genes; MEK1, PLD2, ULK1/2, USP8, and sMAT2A. **(C)** Dose-response curves of worms treated with inhibitors targeting human PLD2, MEK1, ULK1/2, and USP8. Compounds were tested from a range of 50 μM to 10 nM to determine EC_{50}. Values were determined by Prism. Scale bar **(B)**, 1,000 μm.

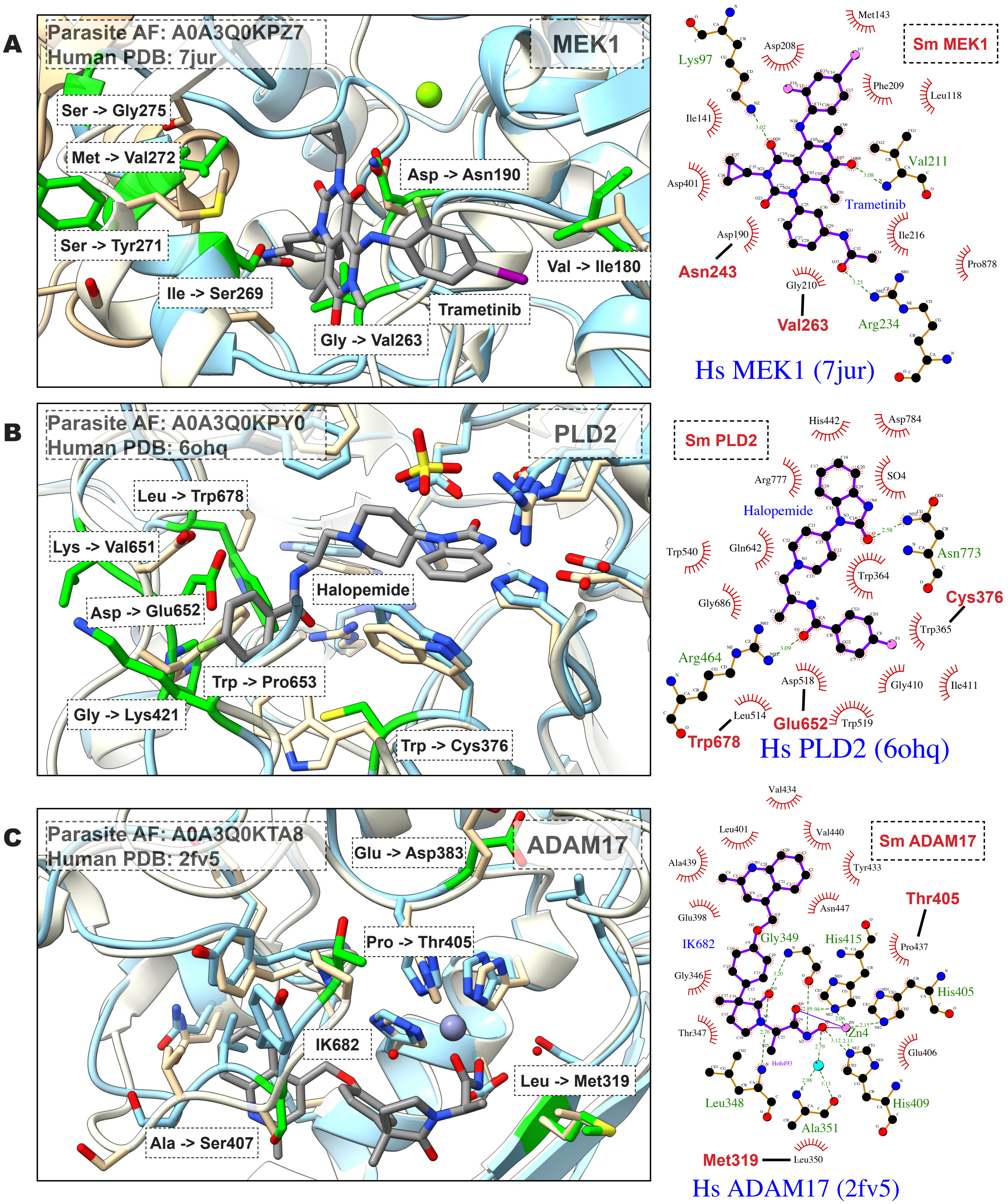

Supplemental Figure 3. 3D homology models of potential schistosome drug targets and their human orthologs

3D alignments of predicted AlphaFold structures of schistosome (blue) proteins overlayed with structures of their closest human (white) homologs. Structures for human proteins bound to their respective inhibitors were retrieved

from the Protein Data Bank for **(A)** MEK1 (PDB: 7JUR), **(B)** PLD2 (PDB: 6OHQ), and **(C)** ADAM17 (PDB: 2FV5). Unique schistosome residues are highlighted with green. Ligplots were generated using LigPlot+ v2.2 using the same PDB structures for human orthologs as listed above. Residues forming hydrophobic interactions are colored black, while other interactions are depicted in green. Unique schistosome residues are outlined in red.

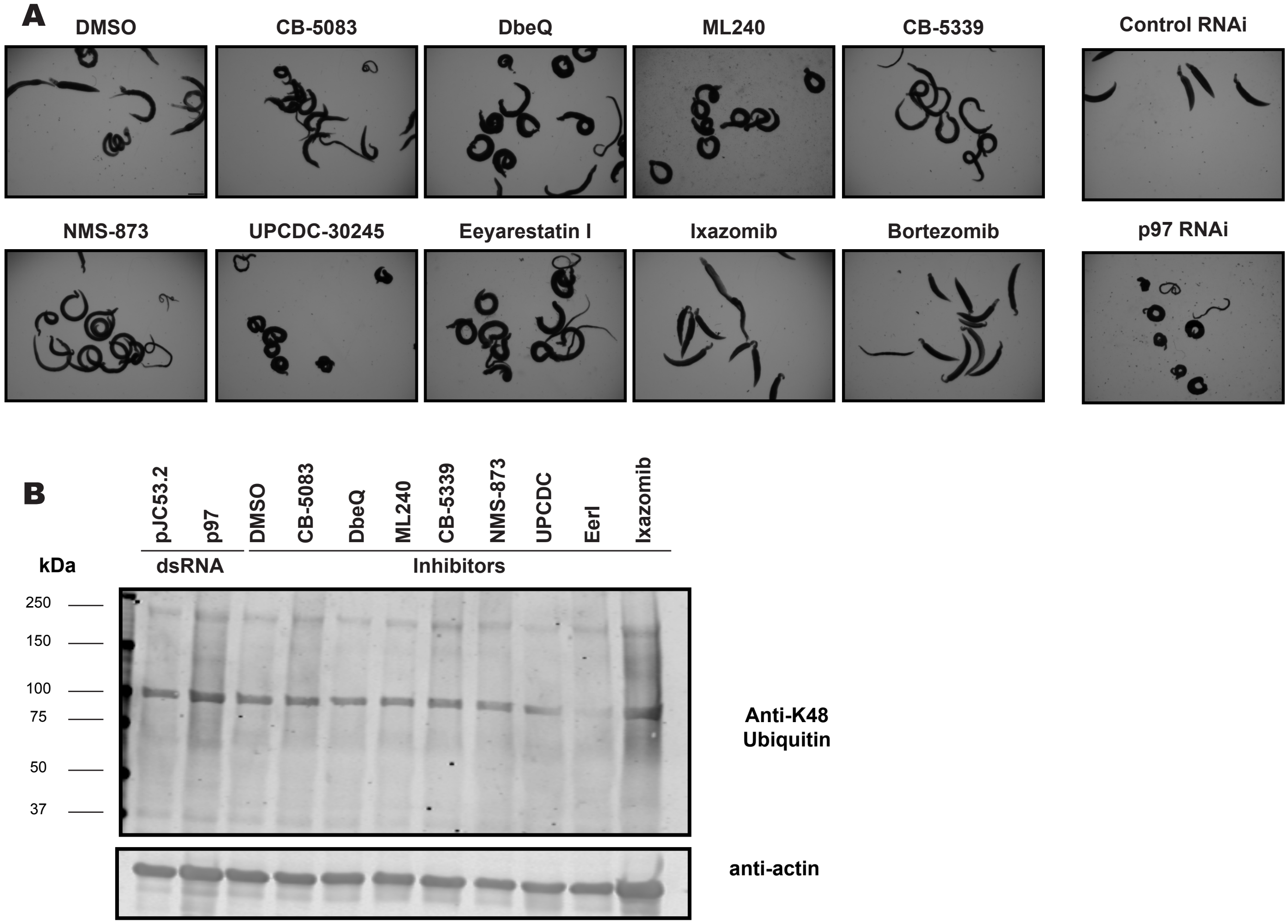

Supplemental Figure 4. Treatment of adult parasites with known human p97 inhibitors

(A) Light microscopy images of adult parasites treated with either dsRNA targeting p97 (pJC53.2 control) or human p97 inhibitors (DMSO control) at 10 µM. **(B)** Western blot depicting polyubiquitinated protein profile (K48 antibody) in worm lysate following treatment of adult worms with p97 dsRNA or inhibitors (actin loading control). Scale bar **(A)**, 1,000 µm.

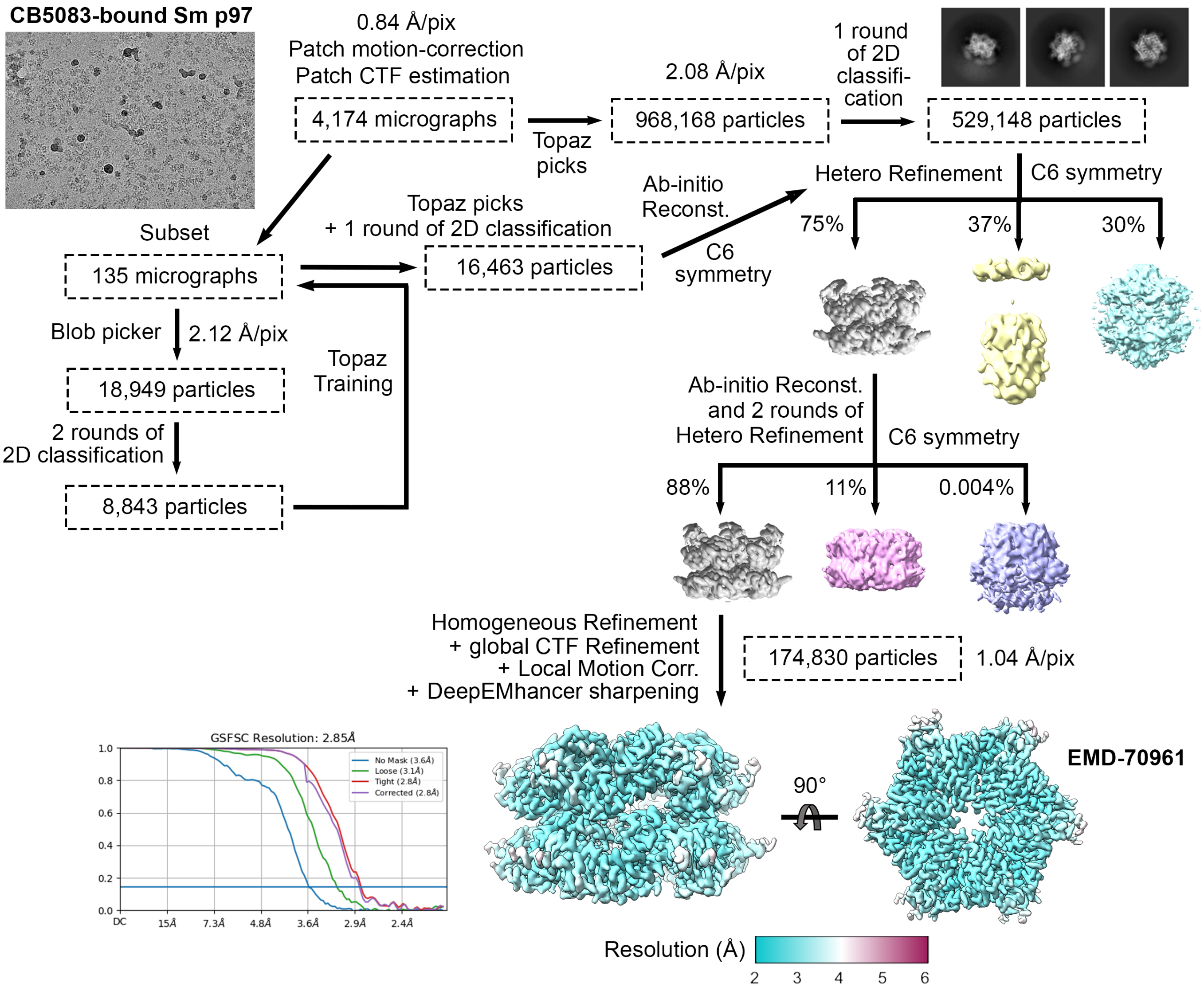

Supplemental Figure 5. Data processing of *S. mansoni* p97 bound to CB-5083

Data processing scheme for cryo-EM dataset involving *S. mansoni* p97 bound to active site inhibitor CB-5083.

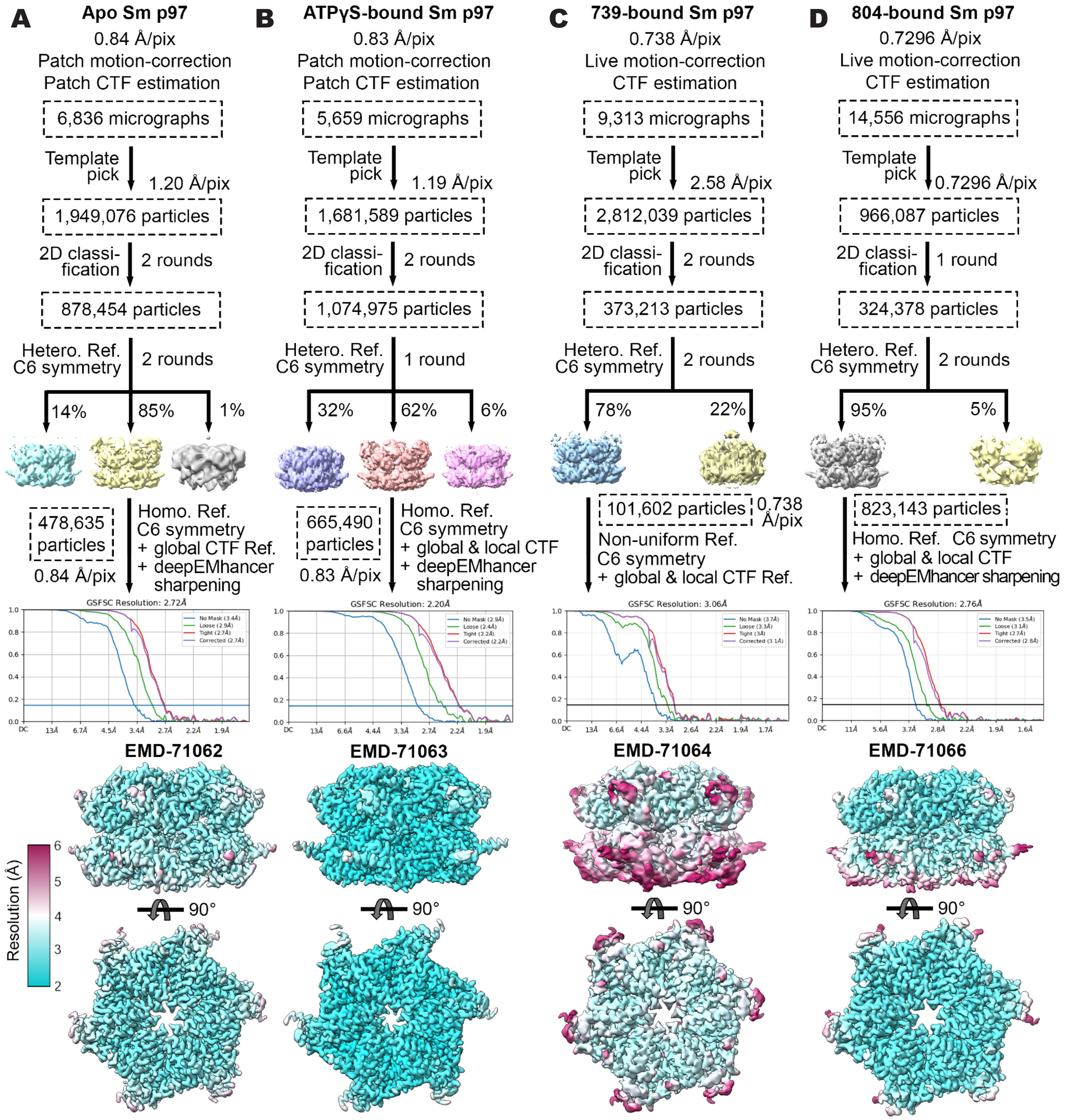

Supplemental Figure 6. Data processing of *S. mansoni* p97 apo enzyme, and bound to ATPγS and covalent inhibitor analogs 739 and 804

(A-D) Data processing scheme for cryo-EM datasets involving *S. mansoni* p97 **(A)** apo enzyme and bound to **(B)** ATPγS and covalent inhibitors, **(C)** 739 and **(D)** 804.

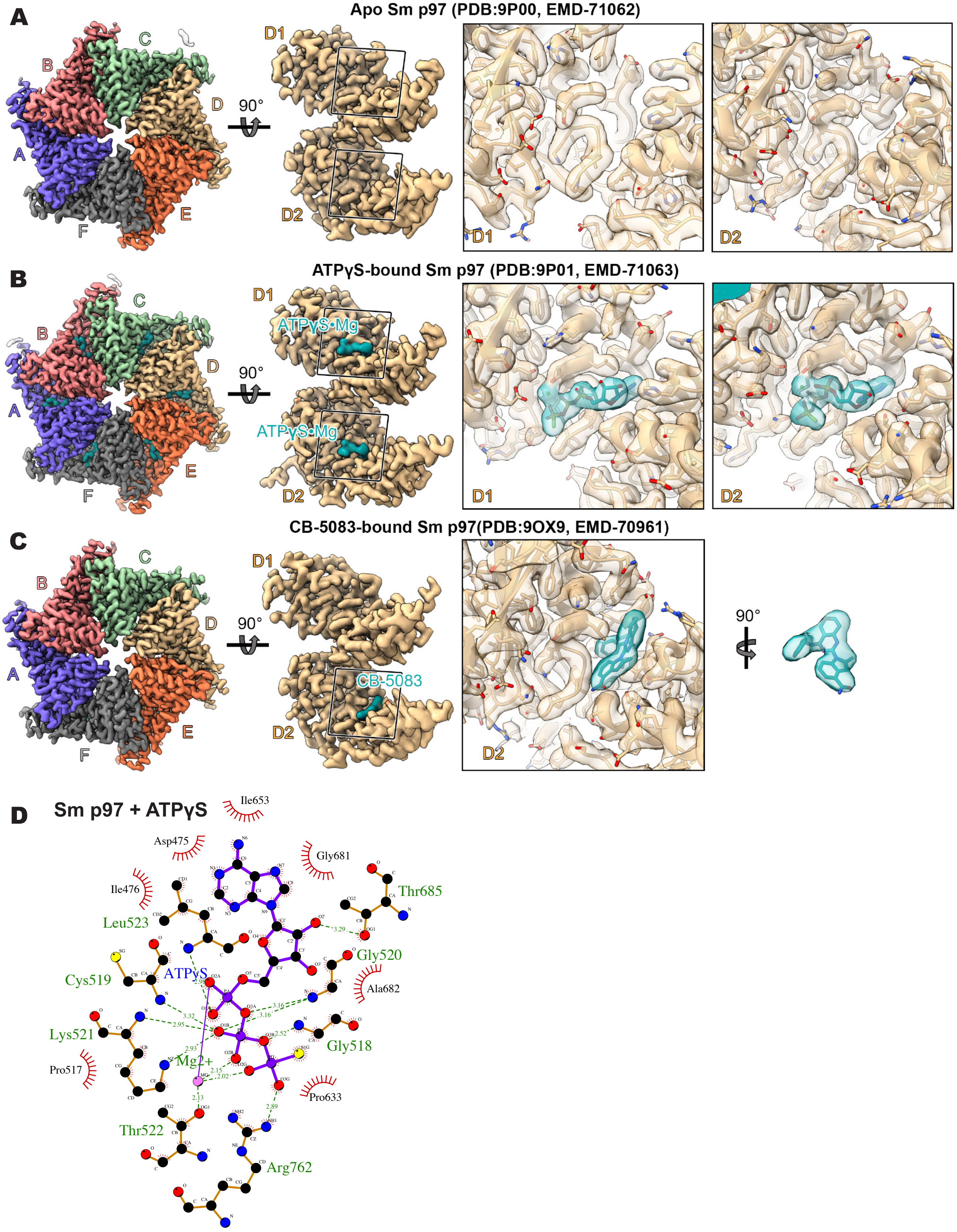

Supplemental Figure 7. Cryo-EM map and structure of *S. mansoni* p97 apo enzyme and bound to known ligands, ATPγS and CB-5083

(A-C) Cryo-EM map of hexamer of schistosome p97 **(A)** apo enzyme or bound to **(B)** ATPγS or **(C)** CB-5083, colored by the final structure. Zoom of ATP binding pocket in the D1 and D2 domain of apo *S. mansoni* p97 (chain D). Boxes on the right show the map quality of the two nucleotide binding pockets. Density for CB-5083 is shown in two views. **(D)** Ligplot of residues involved in *S. mansoni* p97 binding to ATPγS in the D2 domain. Residues forming hydrophobic interactions are colored black, while other interactions are depicted in green.

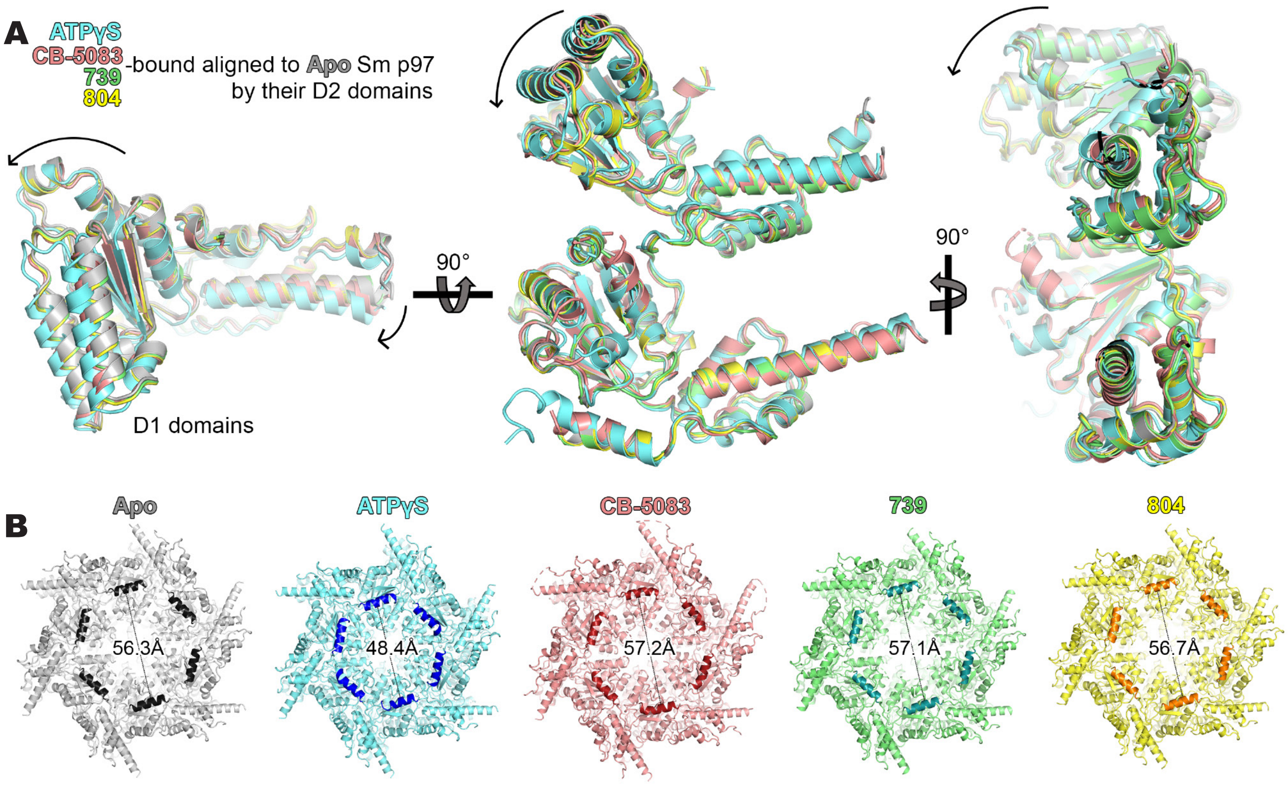

Supplemental Figure 8. Conformational changes induced in *S. mansoni* p97 following ligand binding

Depiction of schistosome p97 conformational changes following binding to nucleotide substrate (ATPγS) and small molecule inhibitors (CB-5083, 739, 804) **(A)** Overlay of *S. mansoni* D1 domains following alignment to apo-enzyme D2 domain. **(B)** Bottom-up view of the *S. mansoni* p97 hexamer. Measurement of the diameter of the central pore of the schistosome p97 between residue K750 of two opposite chains in helix 750-757 (bold) for apo enzyme (grey) compared to ligand-bound states (blue; ATPγS, pink; CB-5083, green; 739, yellow; 804).

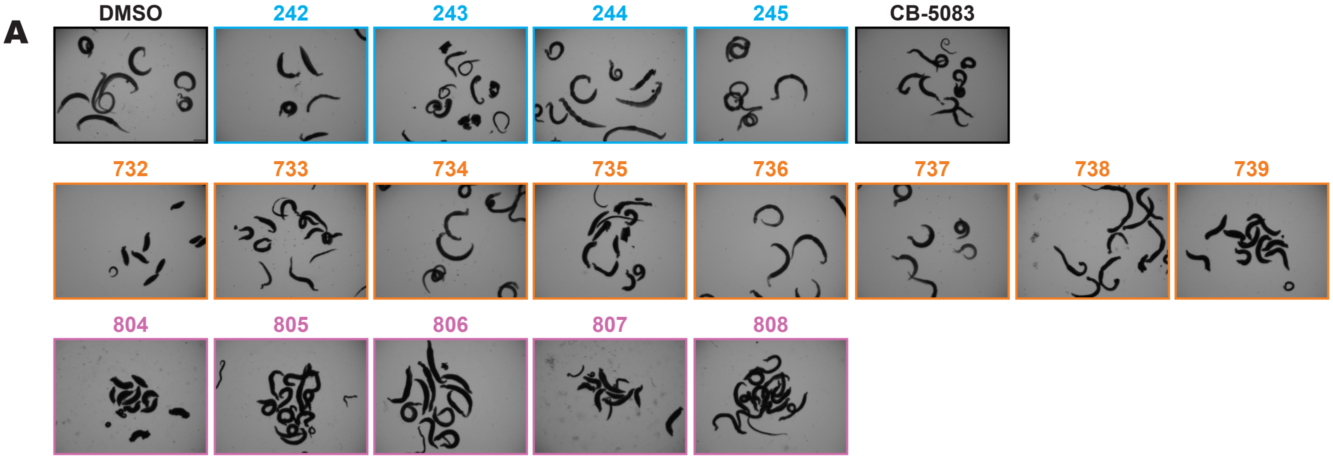

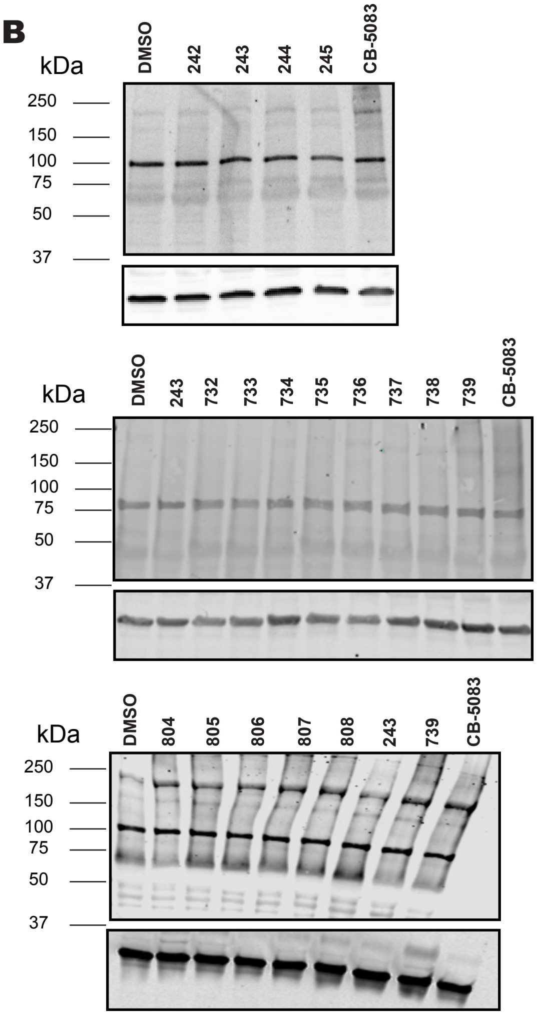

C

Compound	R1	R2	*S. mansoni* IC_{50} (µM)	*H. sapiens* IC_{50} (µM)	Fold Selectivity	S. mansoni EC_{50} (µM)
243	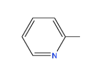	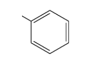	2.1 ± 0.75	20 ± 0.54	9.5	3.5
732	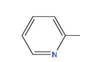	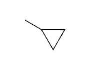	>100	NA	NA	1.3
733	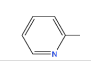	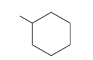	34 ± 1.6	43 ± 4.4	1.3	3.2
734	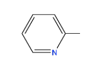	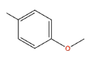	48 ± 2.6	83 ± 6.5	1.7	7.0
735	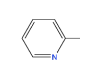	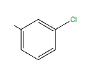	11 ± 0.47	7.3 ± 0.66	0.70	4.2
736	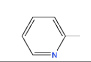	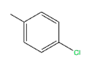	5.9 ± 1.3	6.7 ± 0.055	1.1	12
737	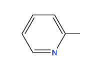	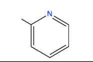	3.0 ± 0.41	13 ± 2.2	4.3	22
738	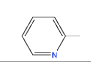	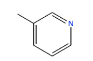	0.42 ± 0.057	0.78 ± 0.14	1.9	3.5
739	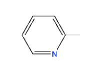	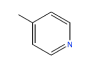	0.15 ± 0.049	0.32 ± 0.026	2.1	1.3
804	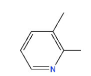	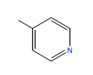	0.19 ± 0.063	0.14 ± 0.062	0.70	0.76
805	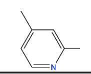	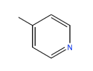	0.17 ± 0.092	0.14 ± 0.078	0.80	2.0
806	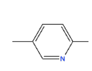	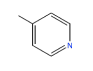	0.47 ± 0.17	0.51 ± 0.16	1.1	3.2
807	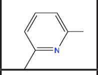	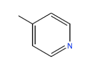	0.67 ± 0.17	0.49 ± 0.057	0.70	1.8
808	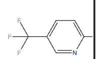	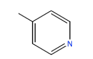	0.18 ± 0.026	0.41 ± 0.084	2.3	2.4

Supplemental Figure 9. Treatment of adult parasites with analogs of covalent p97 scaffold identified in high-throughput screen and known human p97 inhibitors

(A) Light microscopy images of adult parasites treated with controls (negative; DMSO, positive; CB-5083) and analogs of the covalent p97 inhibitor scaffold identified in high-throughput screen. **(B)** Western blot depicting polyubiquitinated protein profile (K48 antibody) in worm lysate following treatment by DMSO control or p97

covalent inhibitor analogs (actin loading control). **(C)** Full structural activity relationship modifications of lead compound series outlining **R1** (800 series) and **R2** (700 series) modifications made to the depicted scaffold. Comparative IC_{50} values display the potency of each compound on the recombinant parasite (*Sm* p97) and human (*Hs* p97) enzyme. Compounds were tested from 100 μM – 1 nM. Values were calculated using Prism. EC_{50} values for benzoxazole propiolamide scaffold analogs on adult parasites determined by fraction of worms attached to tissue culture plate following 72 hours of drug treatment, refreshing media and drug every 24 hr, then allowing worms to remain in culture until D5 (50 μM - 10 nM) (negative; DMSO, positive; CB-5083). Values were calculated using Prism. Scale bar (A), 1,000 μm.

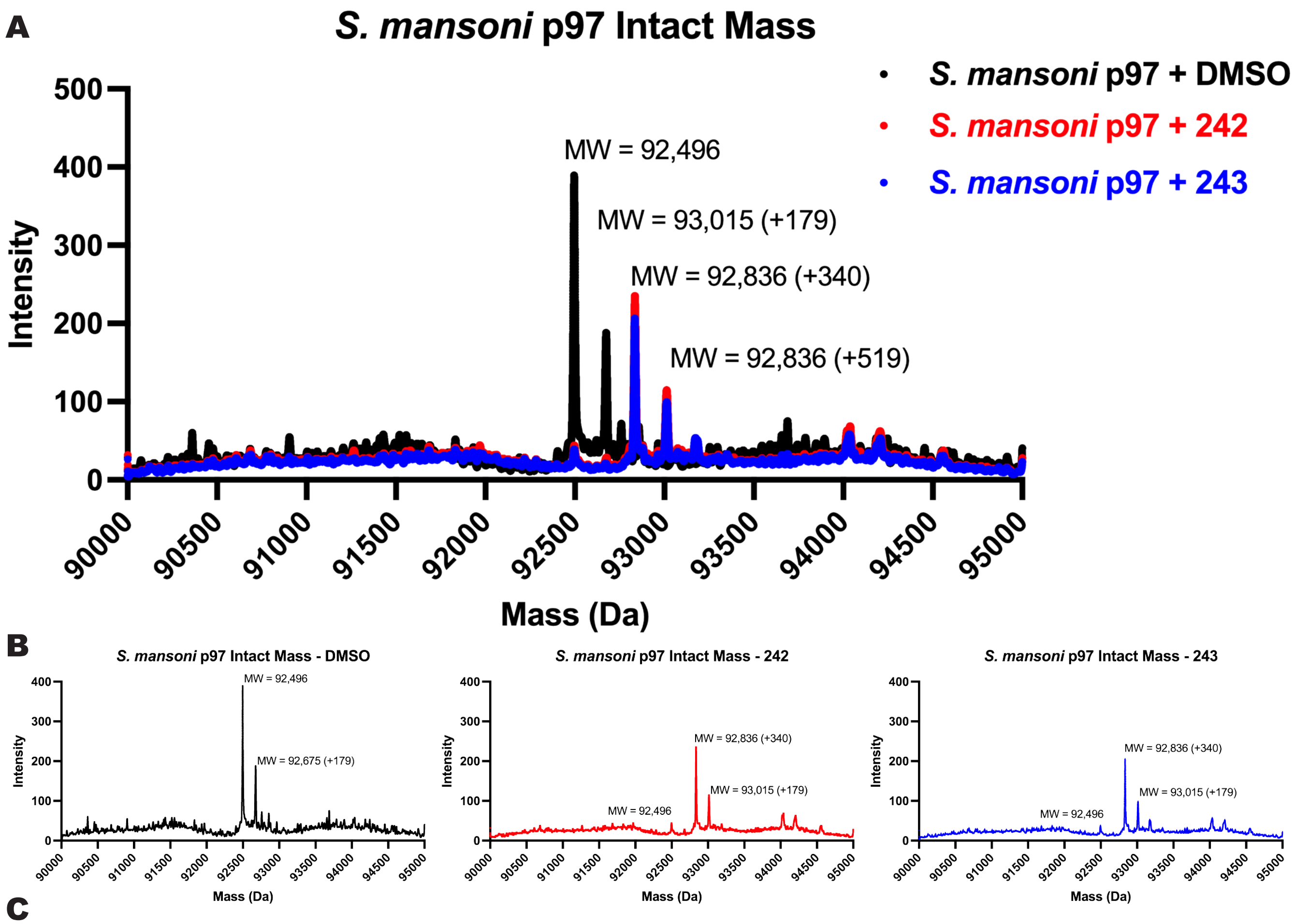

C

Confidence	Annotated Sequence	Modifications	# PSMs	Master Protein Accessions	Positions in Master Proteins	Master Protein Descriptions	Abundance DMSO	Abundance 242	Abundance 243
High	[K].ETVCVAIVDESCPDDKIR.[L]	1xCarbamidomethyl [C]; 1x242 [C]	5	Smp_018240.1	Smp_018240.1 [63-80]	transcript=Smp_018240.1 gene=Smp_018240		1.51E+05	2.02E+06
High	[K].VIETDPSPYCIVSPDTTIHTEGDPVK.[R]	1x242 [C10]	2	Smp_018240.1	Smp_018240.1 [162-187]	transcript=Smp_018240.1 gene=Smp_018240			5.08E+06
High	[K].VIETDPSPYCIVSPDTTIHTEGDPVKR.[E]	1x242 [C10]	3	Smp_018240.1	Smp_018240.1 [162-188]	transcript=Smp_018240.1 gene=Smp_018240			1.29E+07
High	[K].VIETDPSPYCIVSPDTTIHTEGDPVKREDEEEK.[L]	1x242 [C10]	1	Smp_018240.1	Smp_018240.1 [162-194]	transcript=Smp_018240.1 gene=Smp_018240	3.08E+05		5.90E+05
High	[K].REDEEEKLNEIGYDDIGGCR.[K]	1x242 [C19]	2	Smp_018240.1	Smp_018240.1 [188-207]	transcript=Smp_018240.1 gene=Smp_018240			3.26E+06
High	[K].REDEEEKLNEIGYDDIGGCRK.[Q]	1x242 [C19]	1	Smp_018240.1	Smp_018240.1 [188-208]	transcript=Smp_018240.1 gene=Smp_018240			2.02E+06
High	[R].EDEEEKLNEIGYDDIGGCR.[K]	1x242 [C18]	2	Smp_018240.1	Smp_018240.1 [189-207]	transcript=Smp_018240.1 gene=Smp_018240		1.10E+06	1.48E+07
High	[K].LNEIGYDDIGGCR.[K]	1x242 [C12]	1	Smp_018240.1	Smp_018240.1 [195-207]	transcript=Smp_018240.1 gene=Smp_018240			8.32E+06
High	[K].LNEIGYDDIGGCRK.[Q]	1x242 [C12]	1	Smp_018240.1	Smp_018240.1 [195-208]	transcript=Smp_018240.1 gene=Smp_018240			1.47E+07
High	[K].IANEAHGHVGADLASLCSEAALQQIR.[N]	1x242 [C17]	4	Smp_018240.1	Smp_018240.1 [396-421]	transcript=Smp_018240.1 gene=Smp_018240		1.78E+06	5.64E+07
High	[K].FGMTPSKGVLFYGPPGCGK.[T]	1x242 [C17]	1	Smp_018240.1	Smp_018240.1 [503-521]	transcript=Smp_018240.1 gene=Smp_018240		1.01E+05	1.53E+06
**High**	**[K].GVLFYGPPGCGK.[T]**	**1x242 [C10]**	**24**	**Smp_018240.1**	**Smp_018240.1 [510-521]**	**transcript=Smp_018240.1 gene=Smp_018240**		**8.13E+08**	**3.54E+09**
High	[R].QAAPCVLFFDELDSIAK.[A]	1x242 [C5]	2	Smp_018240.1	Smp_018240.1 [565-581]	transcript=Smp_018240.1 gene=Smp_018240		4.97E+06	5.59E+06
High	[K].ATQGFSGADLTEICQR.[A]	1x242 [C14]	1	Smp_018240.1	Smp_018240.1 [675-690]	transcript=Smp_018240.1 gene=Smp_018240			5.89E+06

Supplemental Figure 10. Mass spectrometry of covalent compounds in complex with schistosome p97

(A) Intact mass spectrum of recombinant *S. mansoni* p97 in solution with DMSO control (black) or covalent scaffold compounds (242 - red and 243 - blue). Major peaks consist of a single p97 monomer (~92.5 kDa) and an additional isoform (+179 Da). Mass shifts seen following incubation with either covalent inhibitor (339.35 Da) or DMSO. **(B)** Isolated mass spectrum of recombinant *S. mansoni* p97 in solution with DMSO control (black) or covalent scaffold compounds (242 - red and 243 - blue). **(C)** Table outlining detected peptides following incubation of schistosome p97 with covalent compound 242 or 243. Resulting reaction mixture was run on an SDS-PAGE, then the corresponding band was isolated and submitted for trypsin digest and peptide identification by LC-MS.

A

Compound	243	739	804	805	806	807	808	CB-5083
GI_{50} (μM)	5.6	0.85	0.93	1.2	1.5	1.1	2.6	0.31
IC_{50} (μM)	9.6	1.2	1.2	1.6	1.8	1.4	3.4	0.41

B

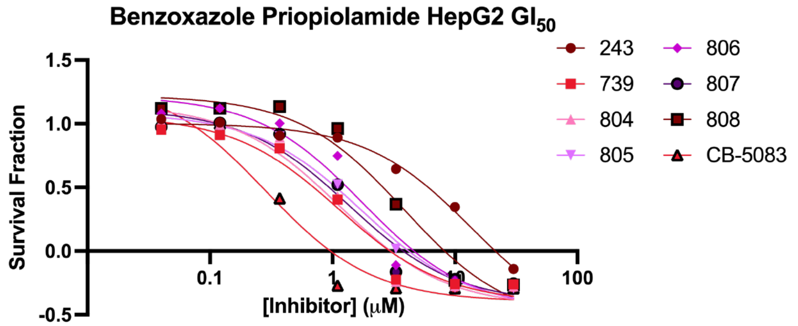

C

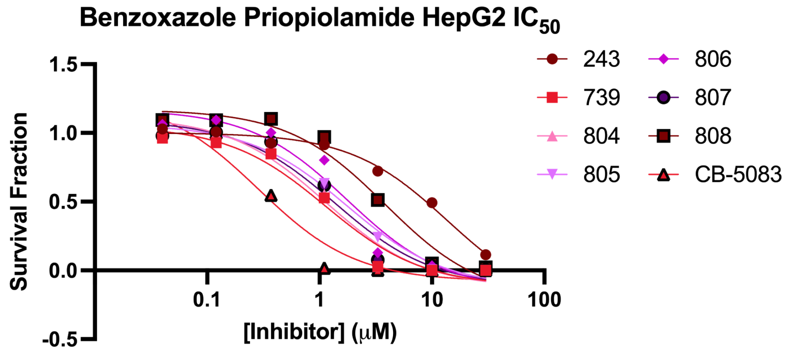

D

Compound	*S. mansoni* IC_{50} (μM)	*H. sapiens* IC_{50} (μM)	Fold Selectivity
242	1.4 ± 0.11	12 ± 0.31	8.6
243	2.1 ± 0.75	20 ± 0.54	9.5
244	>100	>100	NA
245	3.8 ± 0.19	>100	>30
732	>100	NA	NA
733	34 ± 1.6	43 ± 4.4	1.3
734	48 ± 2.6	83 ± 6.5	1.7
735	11 ± 0.47	7.3 ± 0.66	0.70
736	5.9 ± 1.3	6.7 ± 0.055	1.1
737	3.0 ± 0.41	13 ± 2.2	4.3
738	0.42 ± 0.057	0.78 ± 0.14	1.9
739	0.15 ± 0.049	0.32 ± 0.026	2.1
804	0.19 ± 0.063	0.14 ± 0.062	0.70
805	0.17 ± 0.092	0.14 ± 0.078	0.80
806	0.47 ± 0.17	0.51 ± 0.16	1.1
807	0.67 ± 0.17	0.49 ± 0.057	0.70
808	0.18 ± 0.026	0.41 ± 0.084	2.3
CB-5083	0.011 ± 2.1	0.022 ± 0.71	2.0

E

Compound	*S. mansoni* EC_{50} (μM)	HepG2 GI_{50} (μM)	HepG2 IC_{50} (μM)
242	9.9	NA	NA
243	3.5	5.6	9.6
244	25	NA	NA
245	12	NA	NA
732	1.3	NA	NA
733	3.2	NA	NA
734	7.0	NA	NA
735	4.2	NA	NA
736	12	NA	NA
737	22	NA	NA
738	3.5	NA	NA
739	1.3	0.85	1.2
804	0.76	0.93	1.2
805	2.0	1.2	1.6
806	3.2	1.5	1.8
807	1.8	1.1	1.4
808	2.4	2.6	3.4
CB-5083	0.36	0.31	0.41

Supplemental Figure 11. Covalent p97 inhibitor series HepG2 toxicity studies

(A) Table of GI_{50} (50% growth inhibition normalized to time = 0) and IC_{50} (50% growth inhibition relative to control) values for HepG2 cells treated by covalent p97 inhibitor scaffold compared to CB-5083 control. Values were plotted in excel and the 50% point was determined by interpolation. **(B)** Graphs visualized in Prism depicting GI_{50} and **(C)** IC_{50} curves for HepG2 cytotoxicity experiment following treatment by covalent analogs. Table

comparing potencies on **(D)** p97 enzyme (schistosome and human) and **(E)** worms and human cells. Values were calculated in Prism.

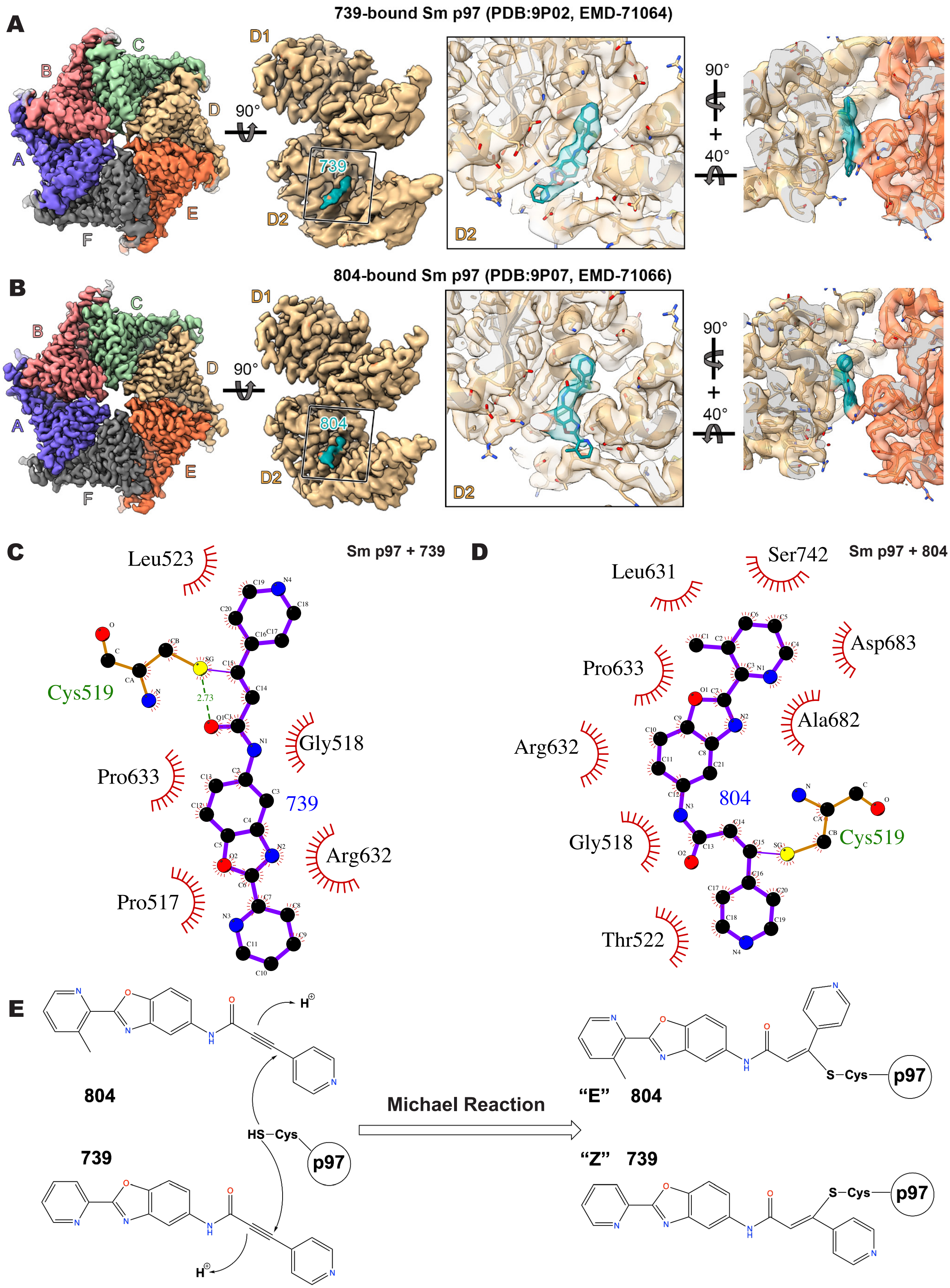

Supplemental Figure 12. Cryo-EM Structure of *S. mansoni* p97 - Compound 739 or 804 complex

(**A-B**) Cryo-EM map of the schistosome p97 bound to compound **(A)** 739 and **(B)** 804, colored by the final structures. Two zoom-in views of the D2 domain of *S. mansoni* p97 is shown on the right. (**C-D**) Ligplot of residues involved in binding to **(C)** 739 and **(D)** 804. **(E)** Schematic depicting Michael addition reaction conducted by the thiol side chain of Cys519 in the schistosome p97 resulting in “E-“ and “Z-“ olefin configuration liganded states for compounds 804 and 739, respectively.

Supporting Information - Benzoxazole propiolamide inhibitor analog chemical synthesis NMR

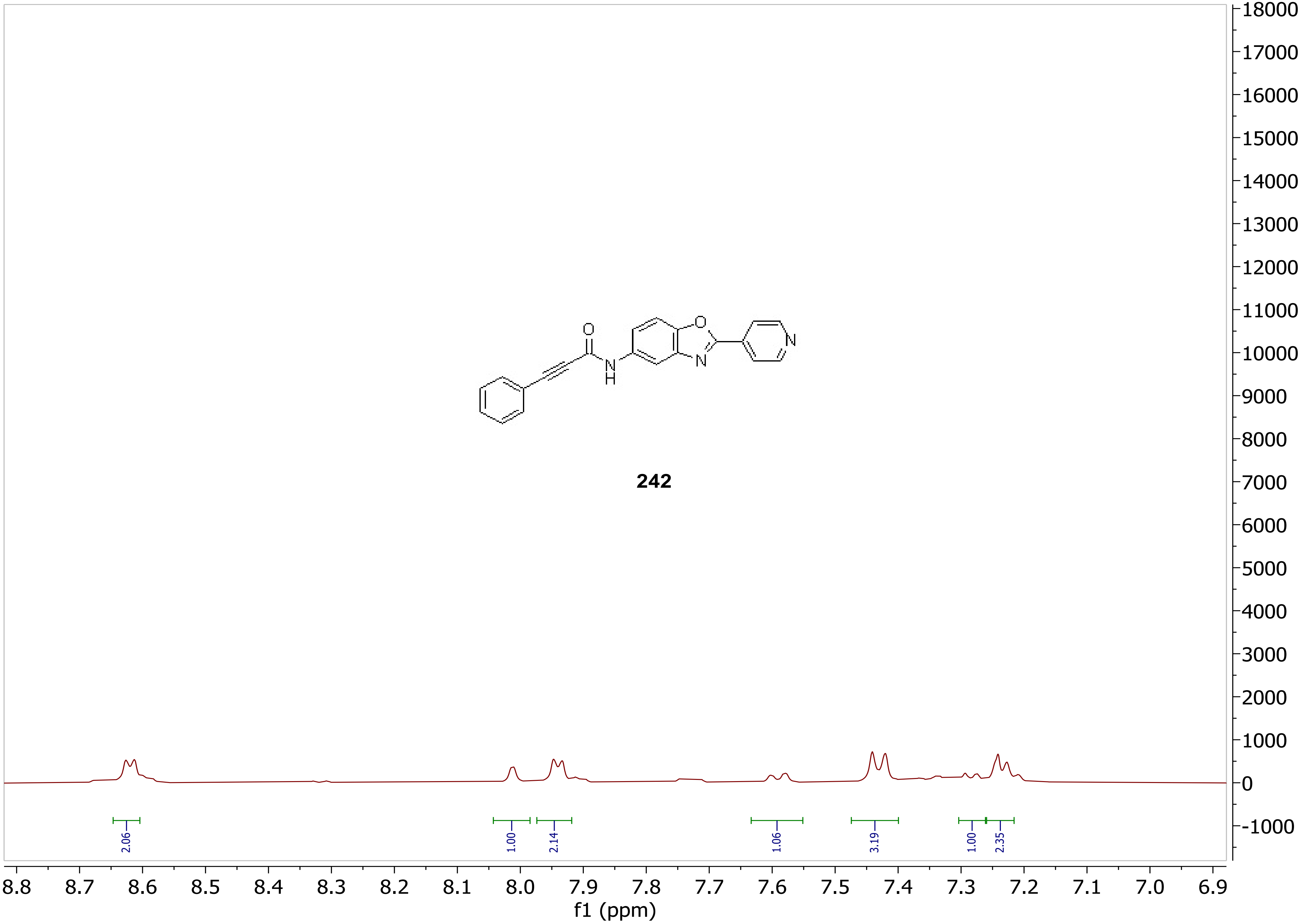

O
N
H
O
N
N
242
2.06
1.00
2.14
1.06
3.19
1.00
2.35
8.8 8.7 8.6 8.5 8.4 8.3 8.2 8.1 8.0 7.9 7.8 7.7 7.6 7.5 7.4 7.3 7.2 7.1 7.0 6.9
f1 (ppm)
18000 17000 16000 15000 14000 13000 12000 11000 10000 9000 8000 7000 6000 5000 4000 3000 2000 1000 0 -1000

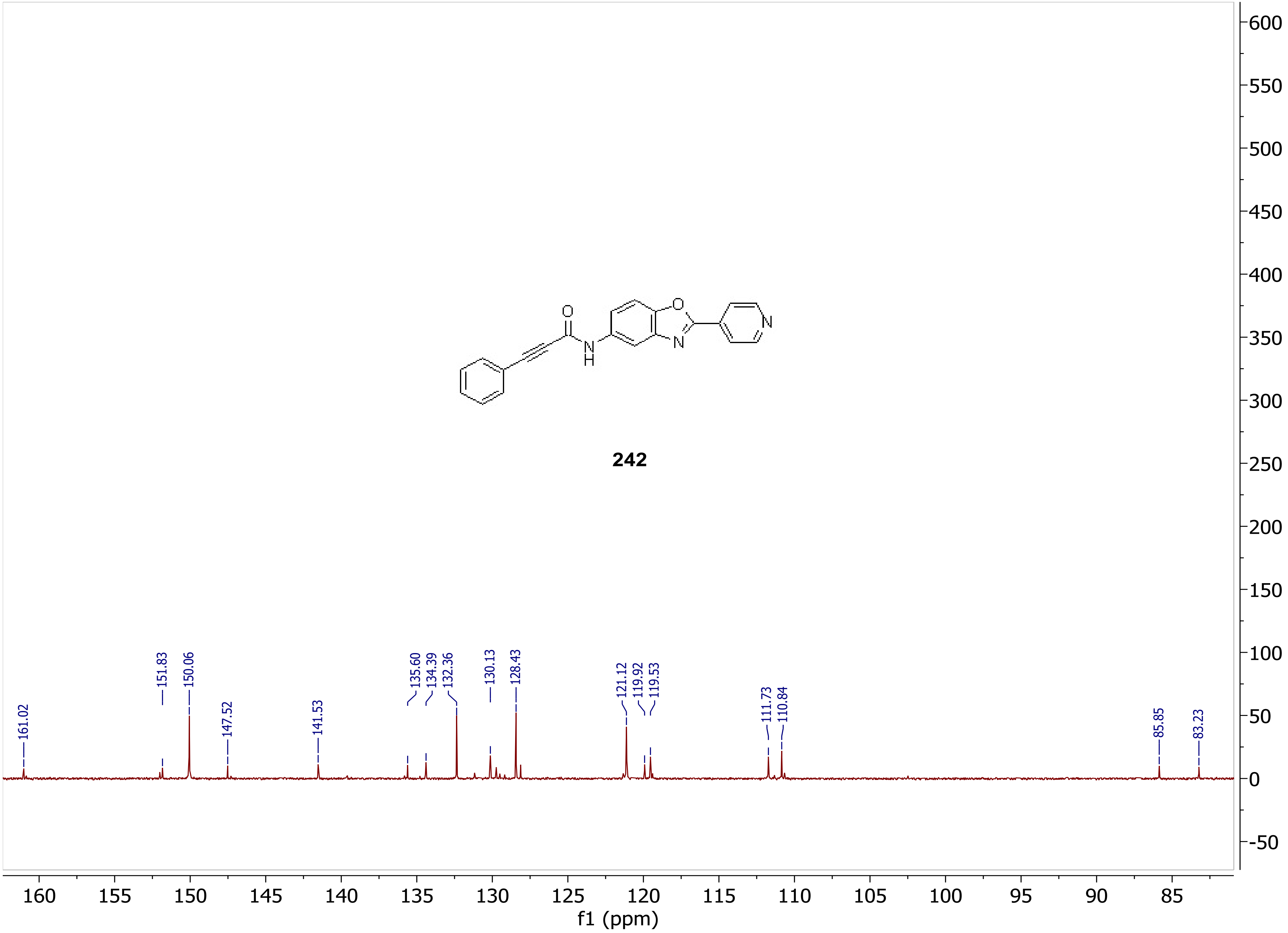
242
161.02
151.83
150.06
147.52
141.53
135.60
134.39
132.36
130.13
128.43
121.12
119.92
119.53
111.73
110.84
85.85
83.23
f1 (ppm)
160
155
150
145
140
135
130
125
120
115
110
105
100
95
90
85
600
550
500
450
400
350
300
250
200
150
100
50
0
-50

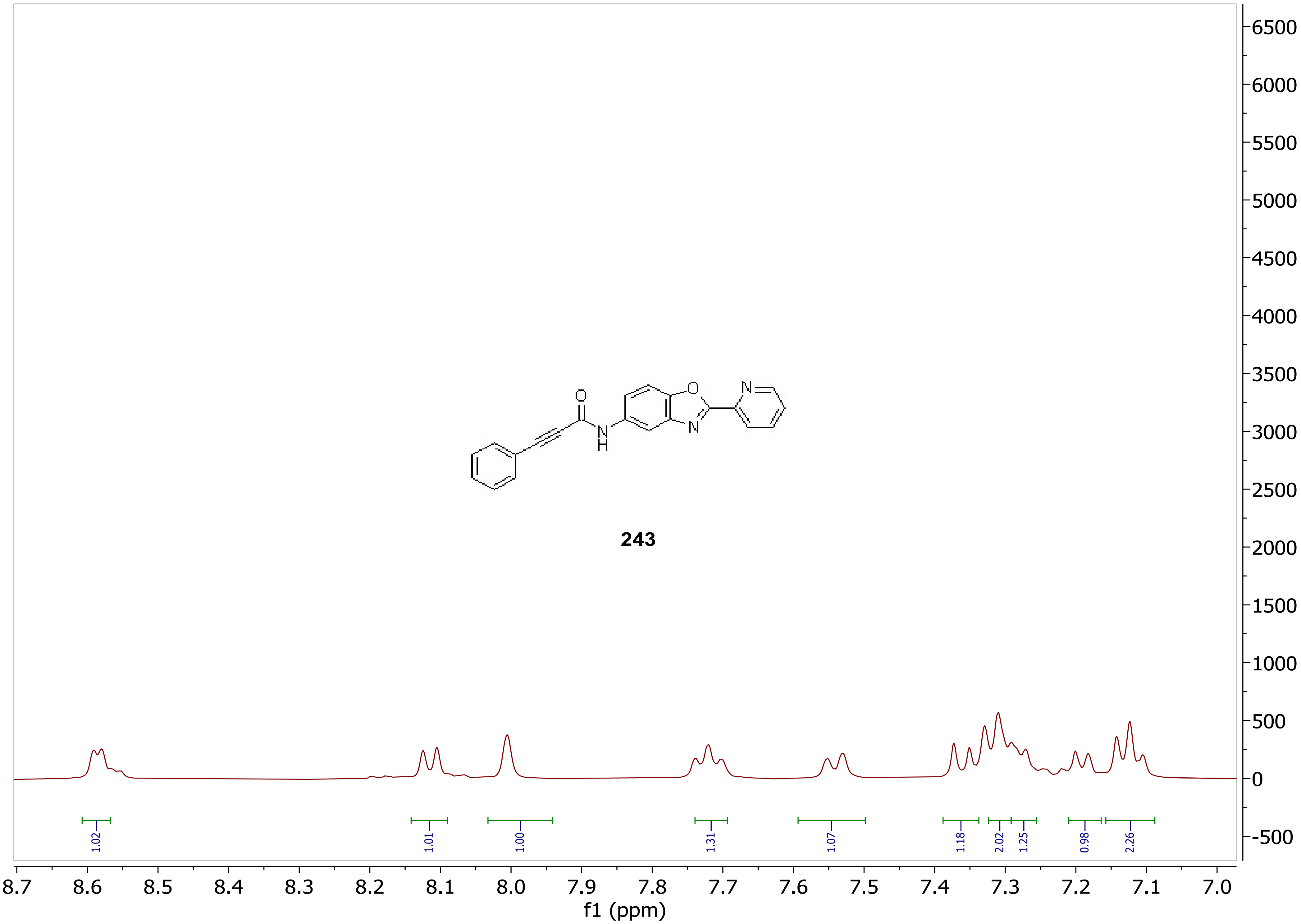
243
O
N
H
O
N
N
1.02
1.01
1.00
1.31
1.07
1.18
2.02
1.25
0.98
2.26
6500
6000
5500
5000
4500
4000
3500
3000
2500
2000
1500
1000
500
0
-500
8.7
8.6
8.5
8.4
8.3
8.2
8.1
8.0
7.9
7.8
7.7
7.6
7.5
7.4
7.3
7.2
7.1
7.0
f1 (ppm)

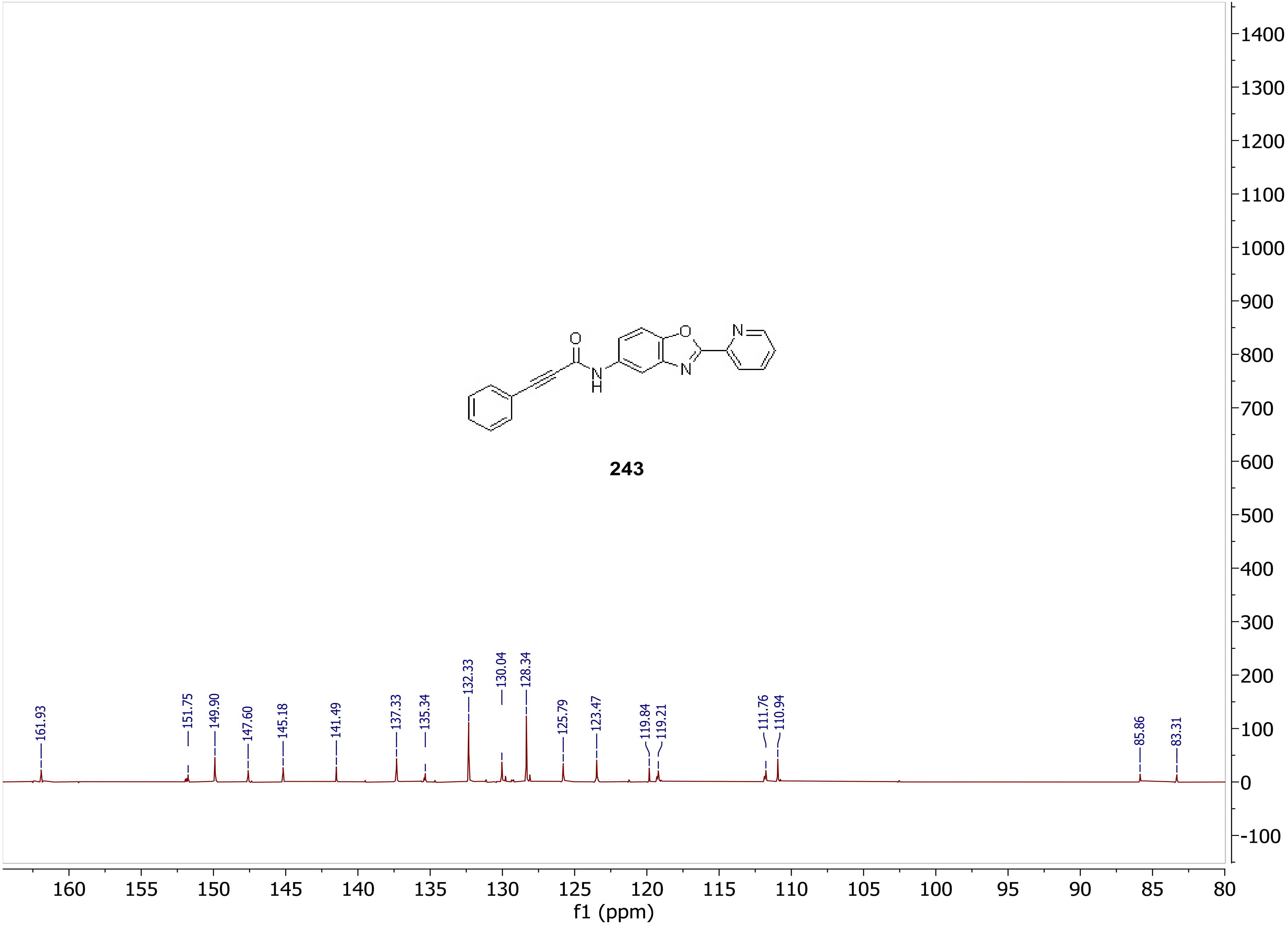

243
161.93
151.75
149.90
147.60
145.18
141.49
137.33
135.34
132.33
130.04
128.34
125.79
123.47
119.84
119.21
111.76
110.94
85.86
83.31
f1 (ppm)
160
155
150
145
140
135
130
125
120
115
110
105
100
95
90
85
80
1400
1300
1200
1100
1000
900
800
700
600
500
400
300
200
100
0
-100

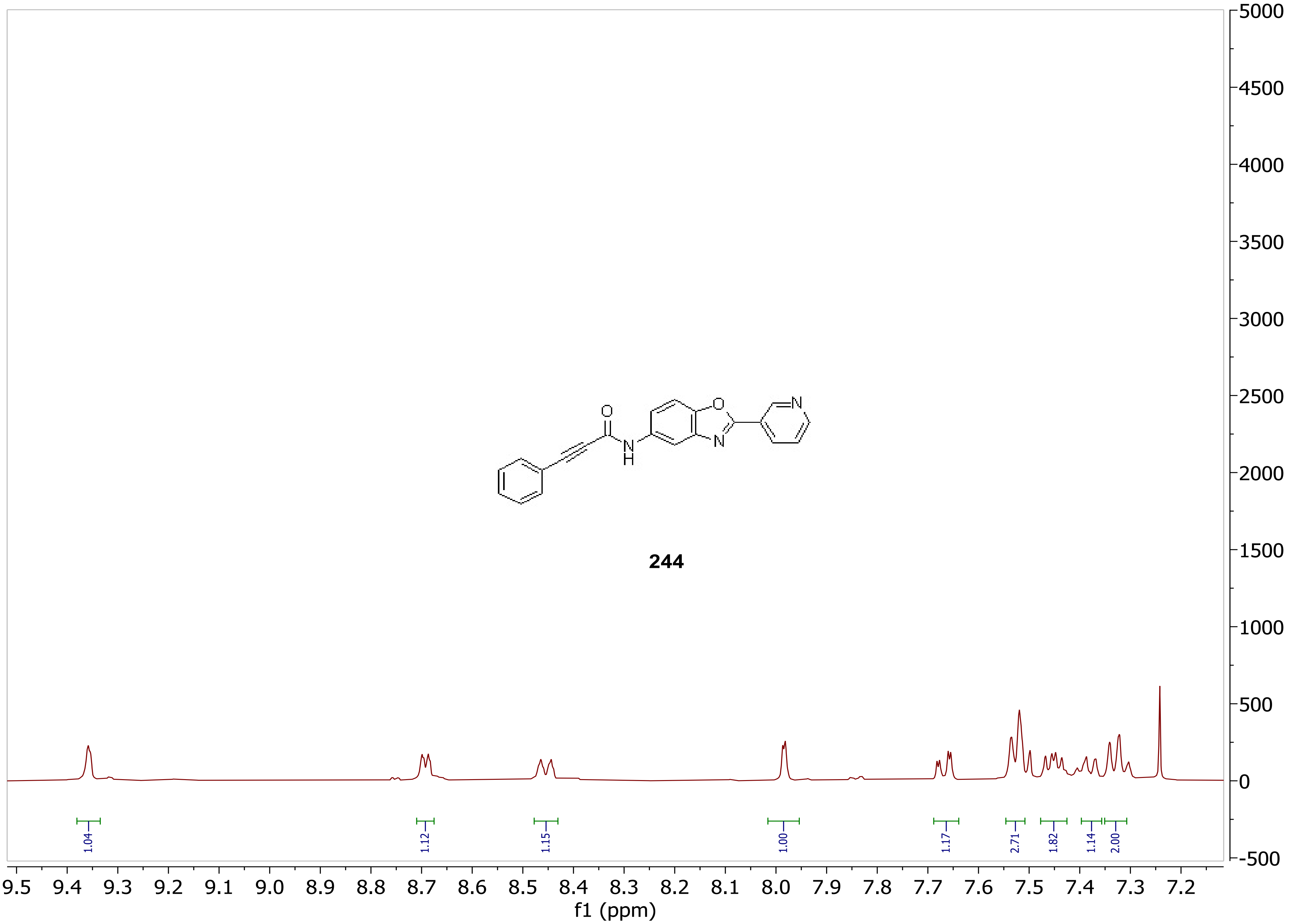

244
O
N
H
N
N
1.04
1.12
1.15
1.00
1.17
2.71
1.82
1.14
2.00
9.5 9.4 9.3 9.2 9.1 9.0 8.9 8.8 8.7 8.6 8.5 8.4 8.3 8.2 8.1 8.0 7.9 7.8 7.7 7.6 7.5 7.4 7.3 7.2
f1 (ppm)
5000 4500 4000 3500 3000 2500 2000 1500 1000 500 0 -500

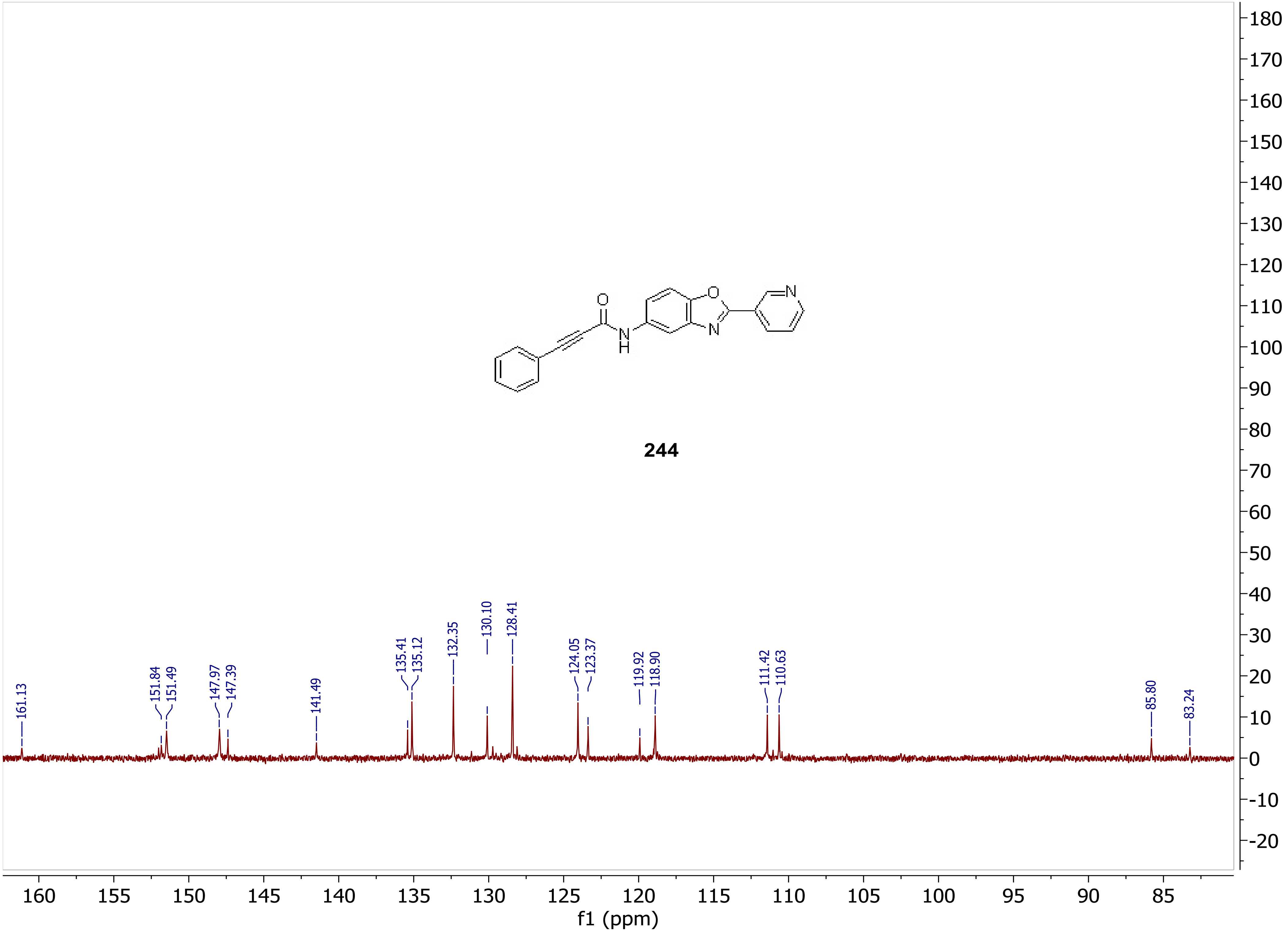

161.13
151.84
151.49
147.97
147.39
141.49
135.41
135.12
132.35
130.10
128.41
124.05
123.37
119.92
118.90
111.42
110.63
85.80
83.24
f1 (ppm)

244

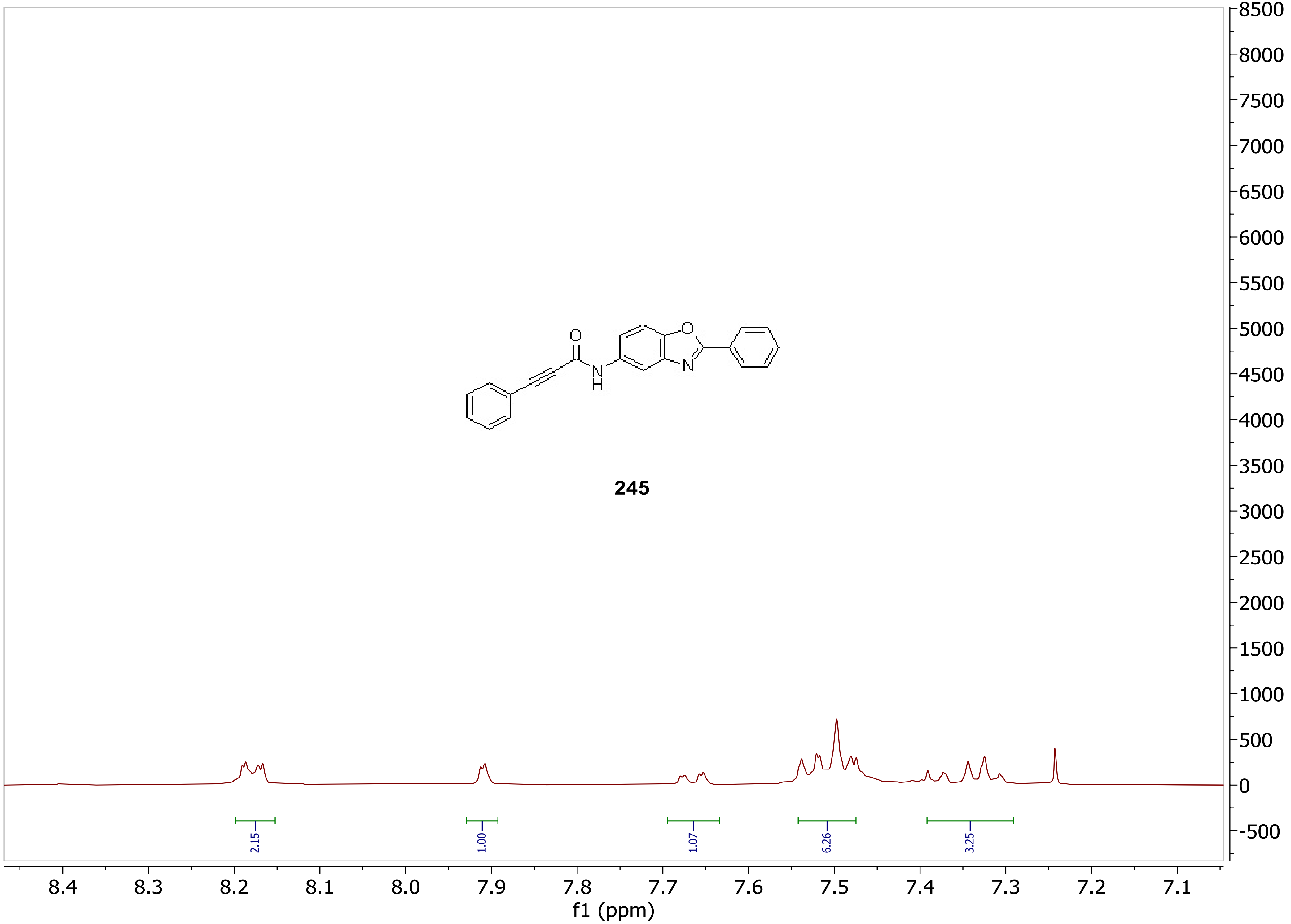
245
O
N
H
O
N
2.15
1.00
1.07
6.26
3.25
8.4
8.3
8.2
8.1
8.0
7.9
7.8
7.7
7.6
7.5
7.4
7.3
7.2
7.1
f1 (ppm)
8500
8000
7500
7000
6500
6000
5500
5000
4500
4000
3500
3000
2500
2000
1500
1000
500
0
-500

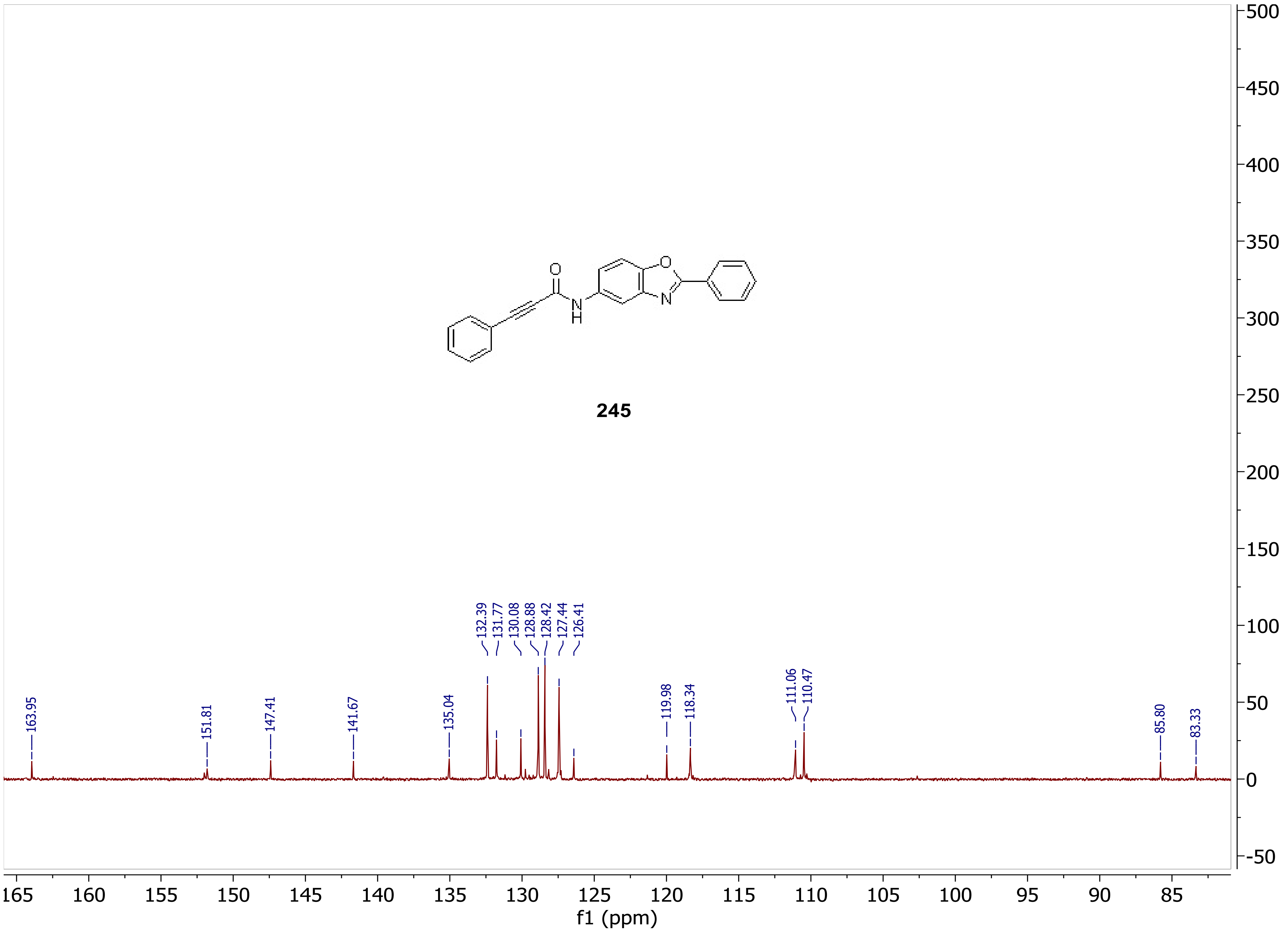
245
163.95
151.81
147.41
141.67
135.04
132.39
131.77
130.08
128.88
128.42
127.44
126.41
119.98
118.34
111.06
110.47
85.80
83.33
f1 (ppm)
165 160 155 150 145 140 135 130 125 120 115 110 105 100 95 90 85
500 450 400 350 300 250 200 150 100 50 0 -50

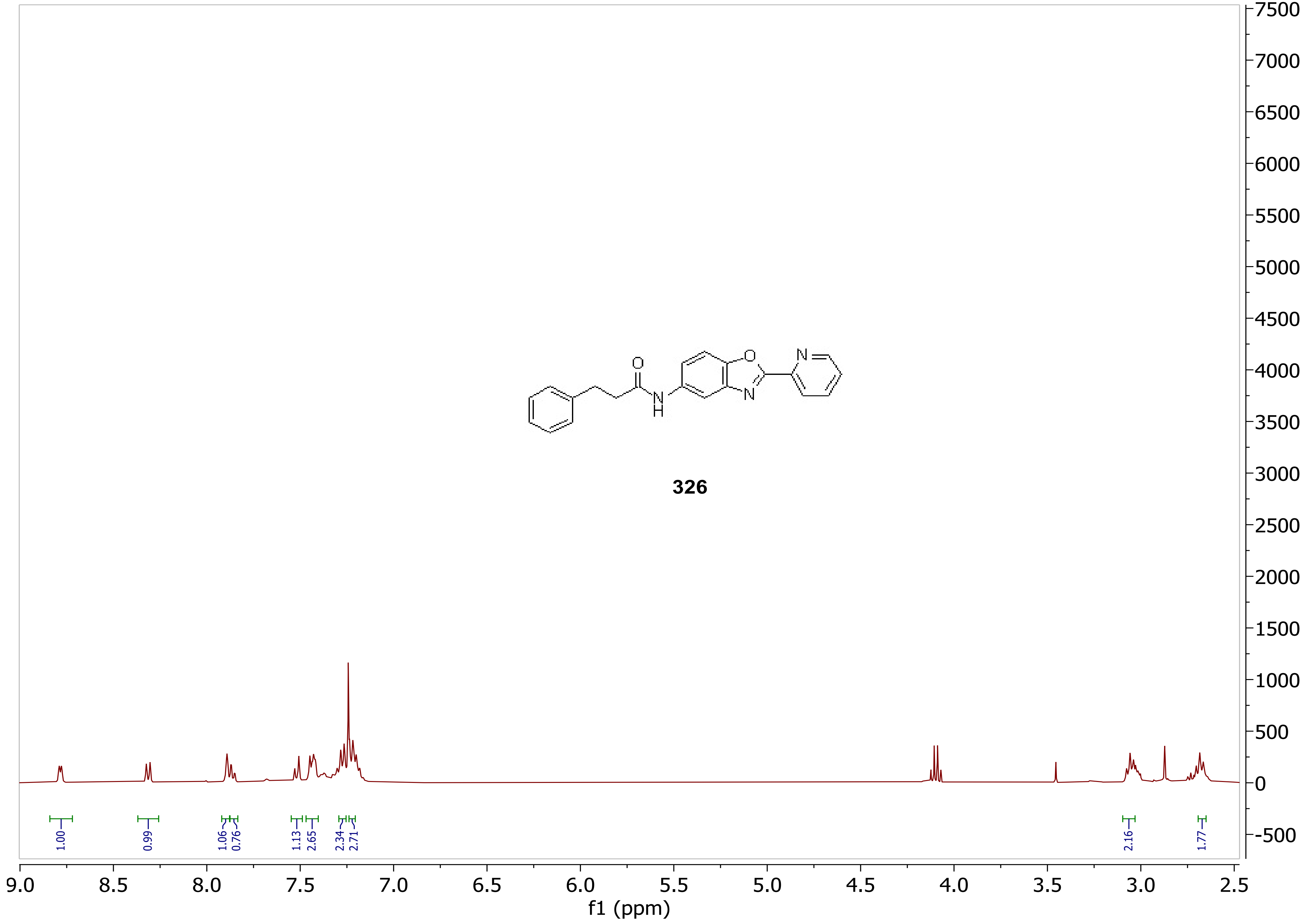
326
1.00
0.99
1.06
0.76
1.13
2.65
2.34
2.71
2.16
1.77
9.0 8.5 8.0 7.5 7.0 6.5 6.0 5.5 5.0 4.5 4.0 3.5 3.0 2.5
f1 (ppm)
7500 7000 6500 6000 5500 5000 4500 4000 3500 3000 2500 2000 1500 1000 500 0 -500

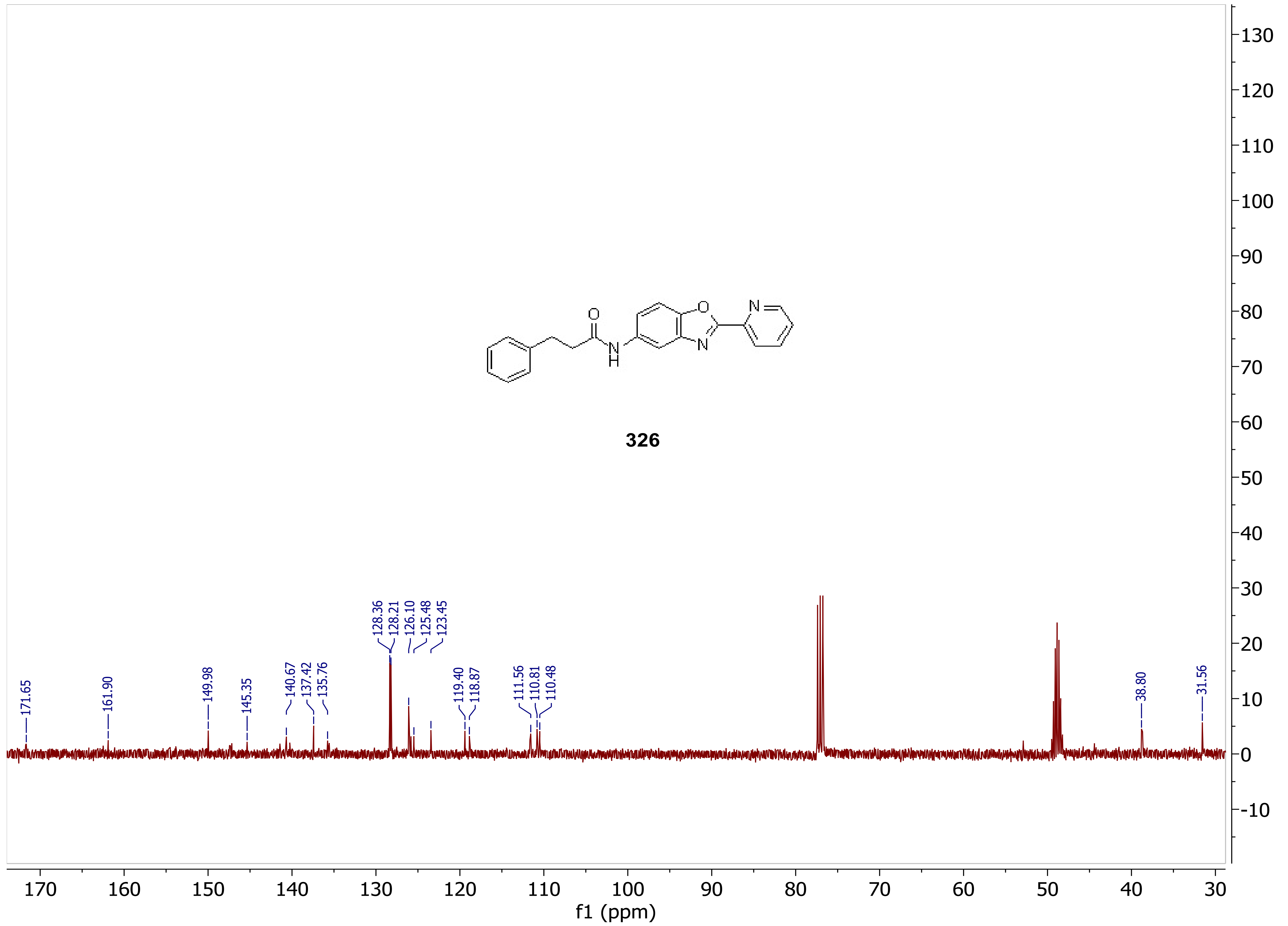
326
171.65
161.90
149.98
145.35
140.67
137.42
135.76
128.36
128.21
126.10
125.48
123.45
119.40
118.87
111.56
110.81
110.48
38.80
31.56
170 160 150 140 130 120 110 100 90 80 70 60 50 40 30
f1 (ppm)
130 120 110 100 90 80 70 60 50 40 30 20 10 0 -10

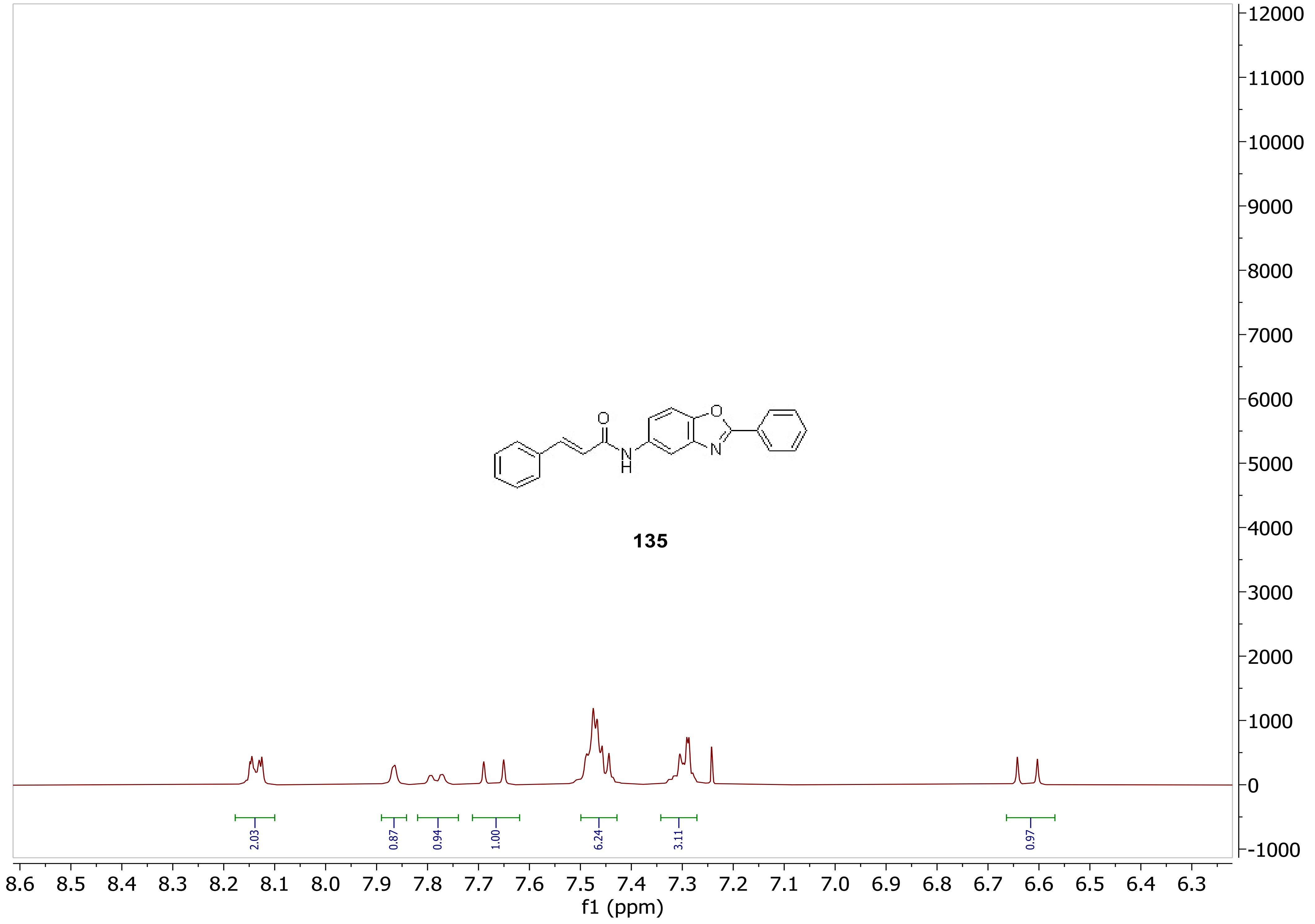

O
N
H
O
N
135
2.03
0.87
0.94
1.00
6.24
3.11
0.97
12000
11000
10000
9000
8000
7000
6000
5000
4000
3000
2000
1000
0
-1000
8.6 8.5 8.4 8.3 8.2 8.1 8.0 7.9 7.8 7.7 7.6 7.5 7.4 7.3 7.2 7.1 7.0 6.9 6.8 6.7 6.6 6.5 6.4 6.3
f1 (ppm)

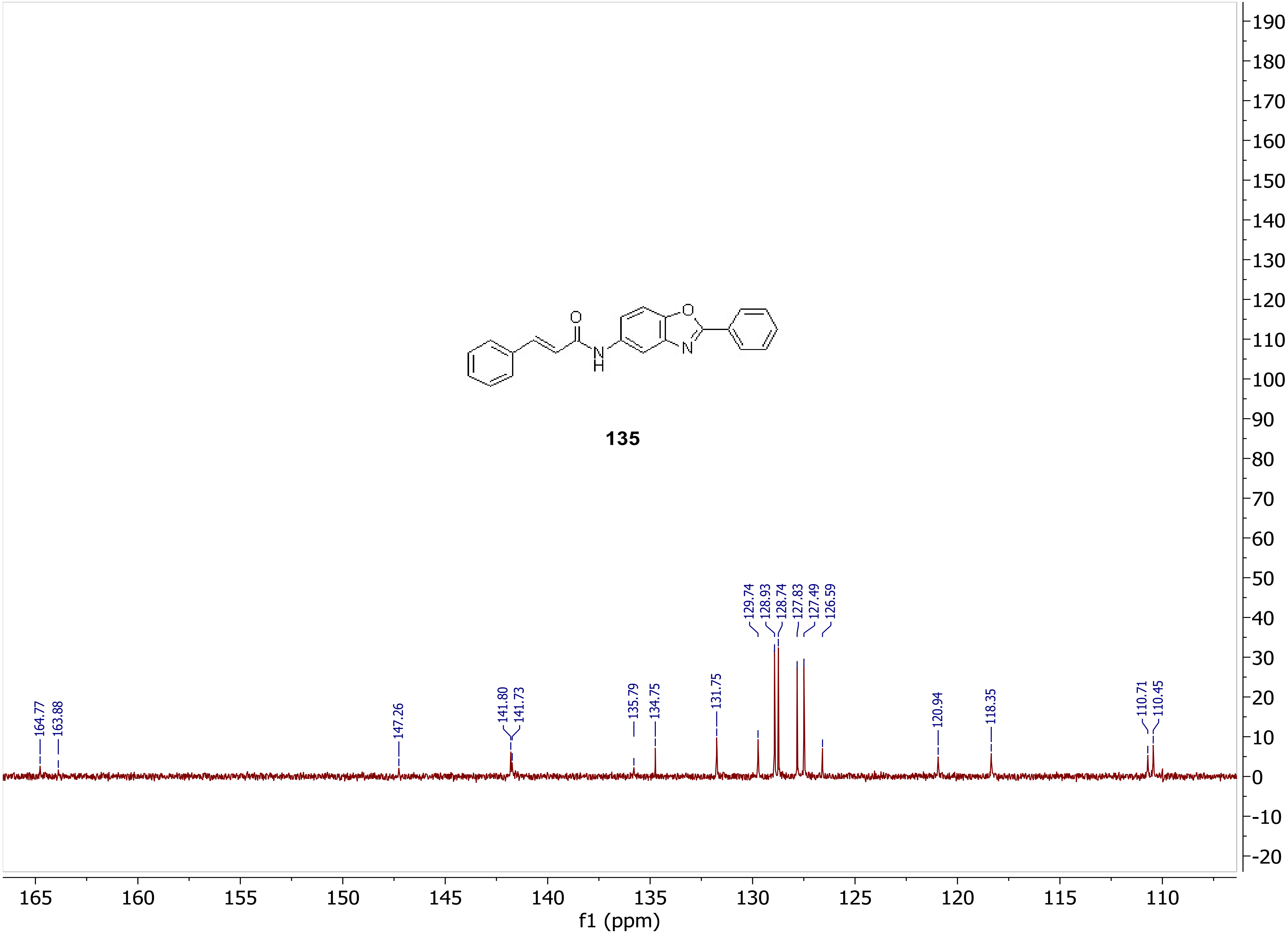

135
164.77
163.88
147.26
141.80
141.73
135.79
134.75
131.75
129.74
128.93
128.74
127.83
127.49
126.59
120.94
118.35
110.71
110.45
f1 (ppm)

JL05-208-084-4p-1H
JL05-208-084-4p: after filtration, 1H in CDCl3 w. 6D d4-MeOH on 400MHz

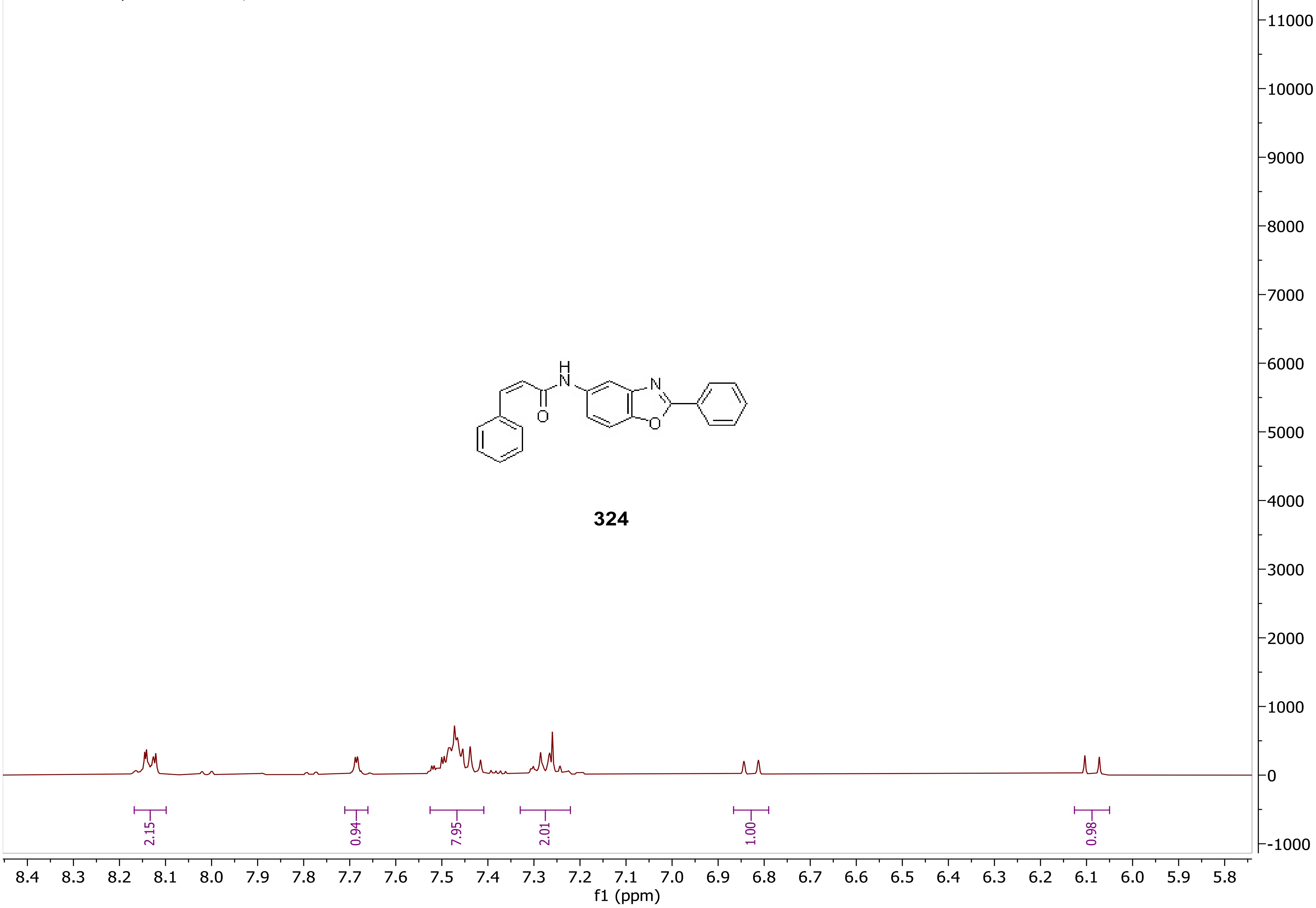

324

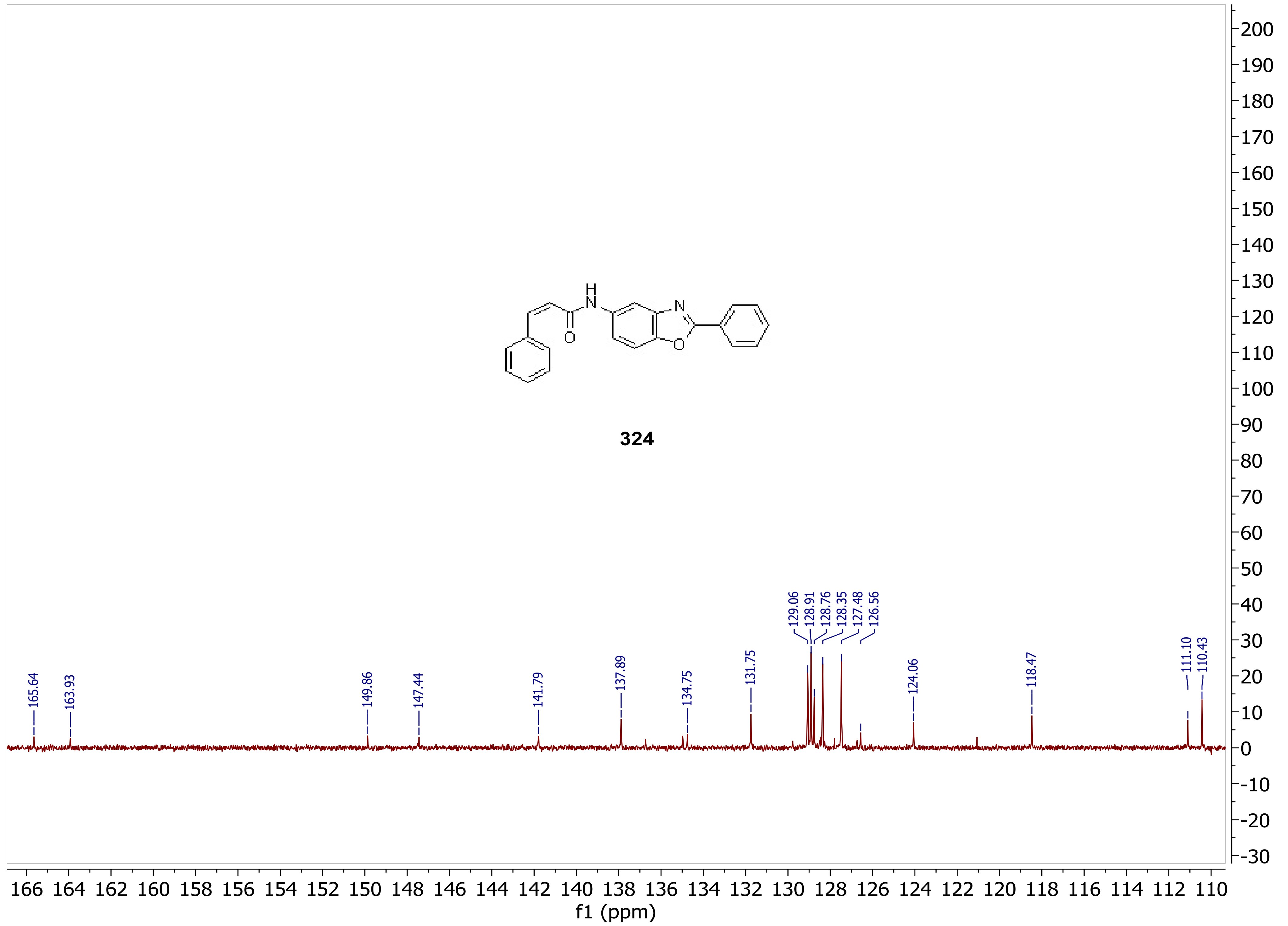

H
N
O
N
O
324
165.64
163.93
149.86
147.44
141.79
137.89
134.75
131.75
129.06
128.91
128.76
128.35
127.48
126.56
124.06
118.47
111.10
110.43
f1 (ppm)

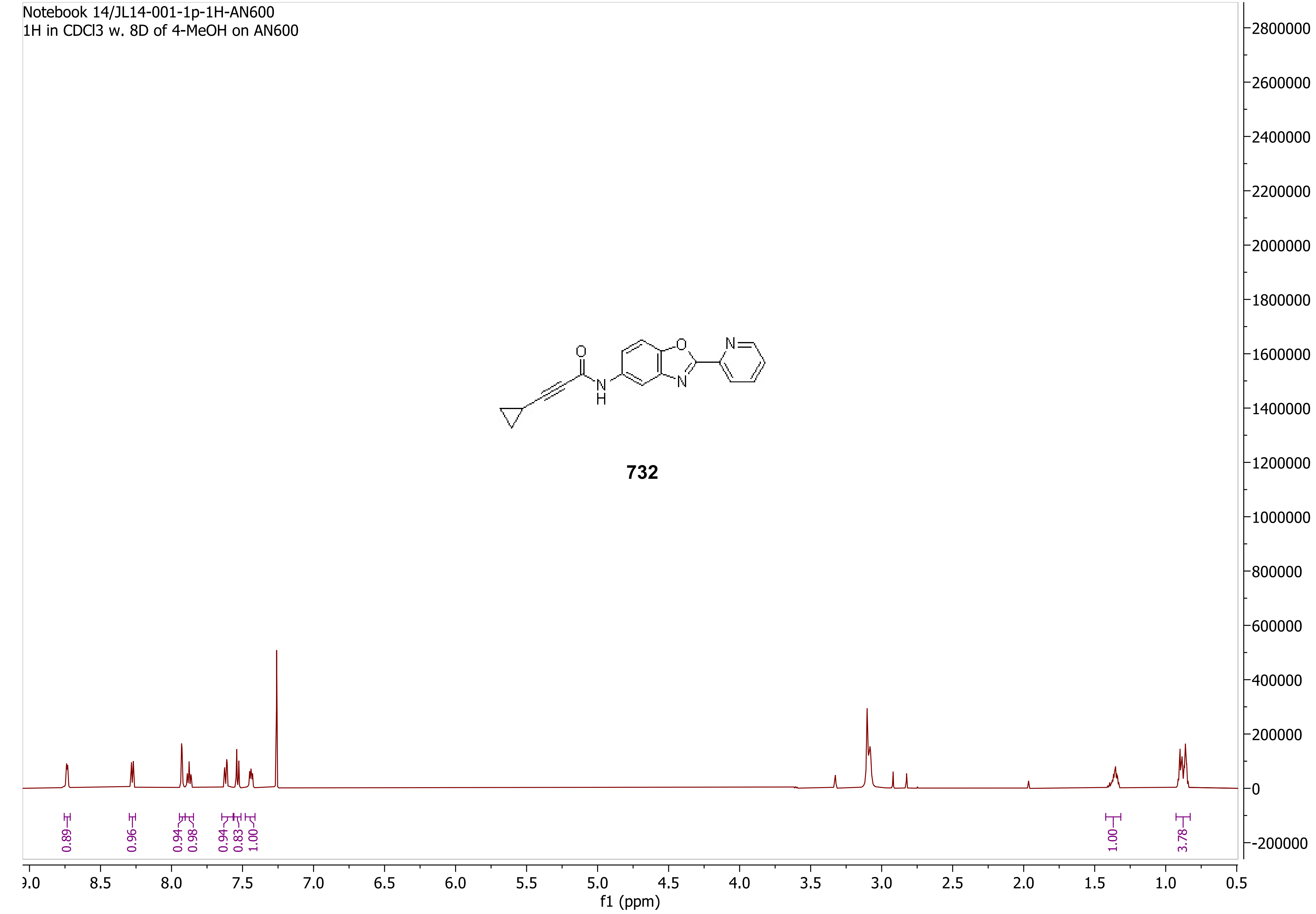

Notebook 14/JL14-001-1p-1H-AN600
1H in CDCl3 w. 8D of 4-MeOH on AN600
732
0.89
0.96
0.94
0.98
0.94
0.83
1.00
1.00
3.78
2800000
2600000
2400000
2200000
2000000
1800000
1600000
1400000
1200000
1000000
800000
600000
400000
200000
0
-200000
9.0
8.5
8.0
7.5
7.0
6.5
6.0
5.5
5.0
4.5
4.0
3.5
3.0
2.5
2.0
1.5
1.0
0.5
f1 (ppm)

Notebook 14/JL14-001-1p-13C-AN600
13C in CDCl3 w. 8D of 4-MeOH on AN600

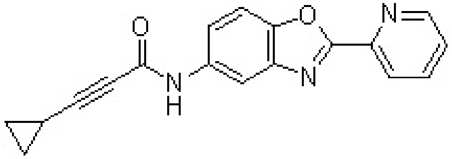

732

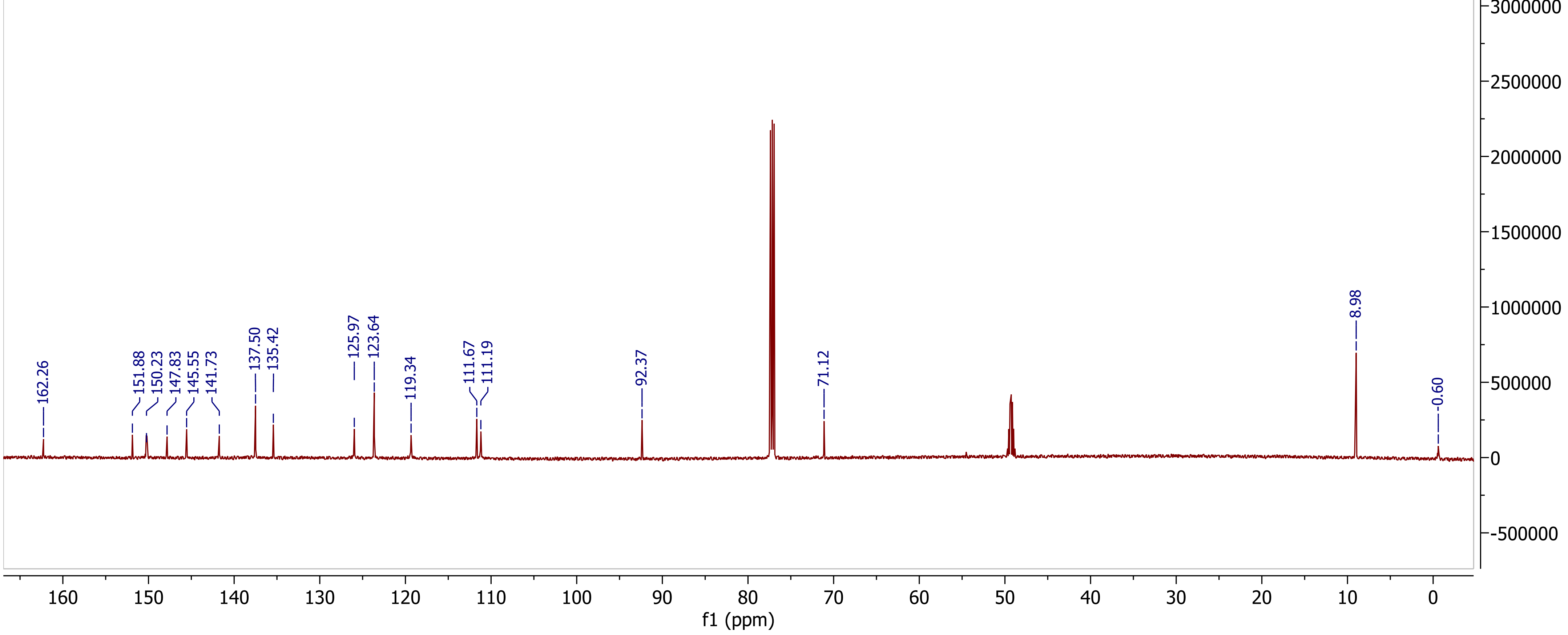

Notebook 14/JL14-001-2p-1H-AN600
off-white solid after water wash, 1H in CDCl3 w. 8D of d4-MeOH on AN600

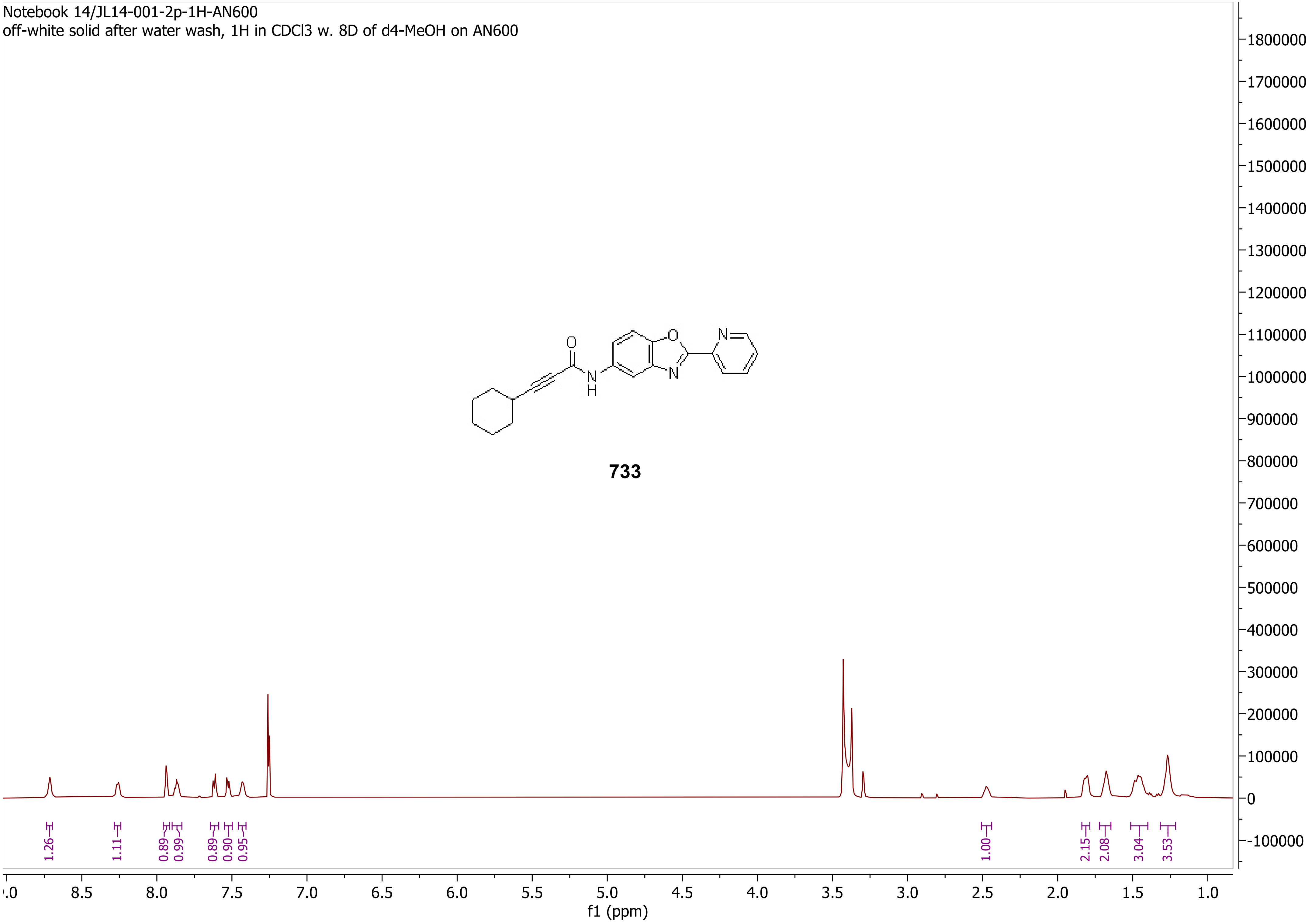

Notebook 14/JL14-001-2p-13C-AN600
off-white solid after water wash, 13C in CDCl3 w. 8D of d4-MeOH on AN600

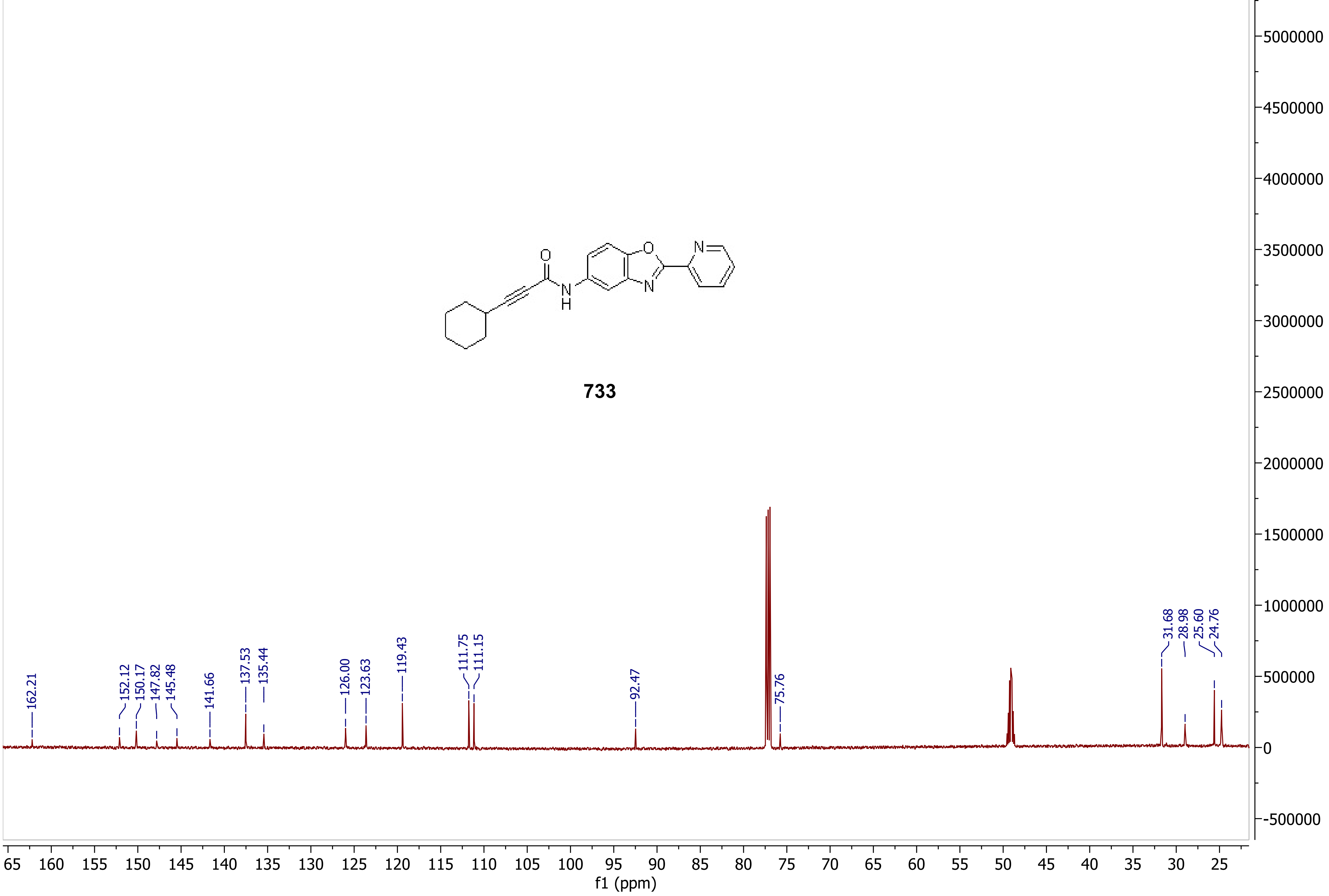

733

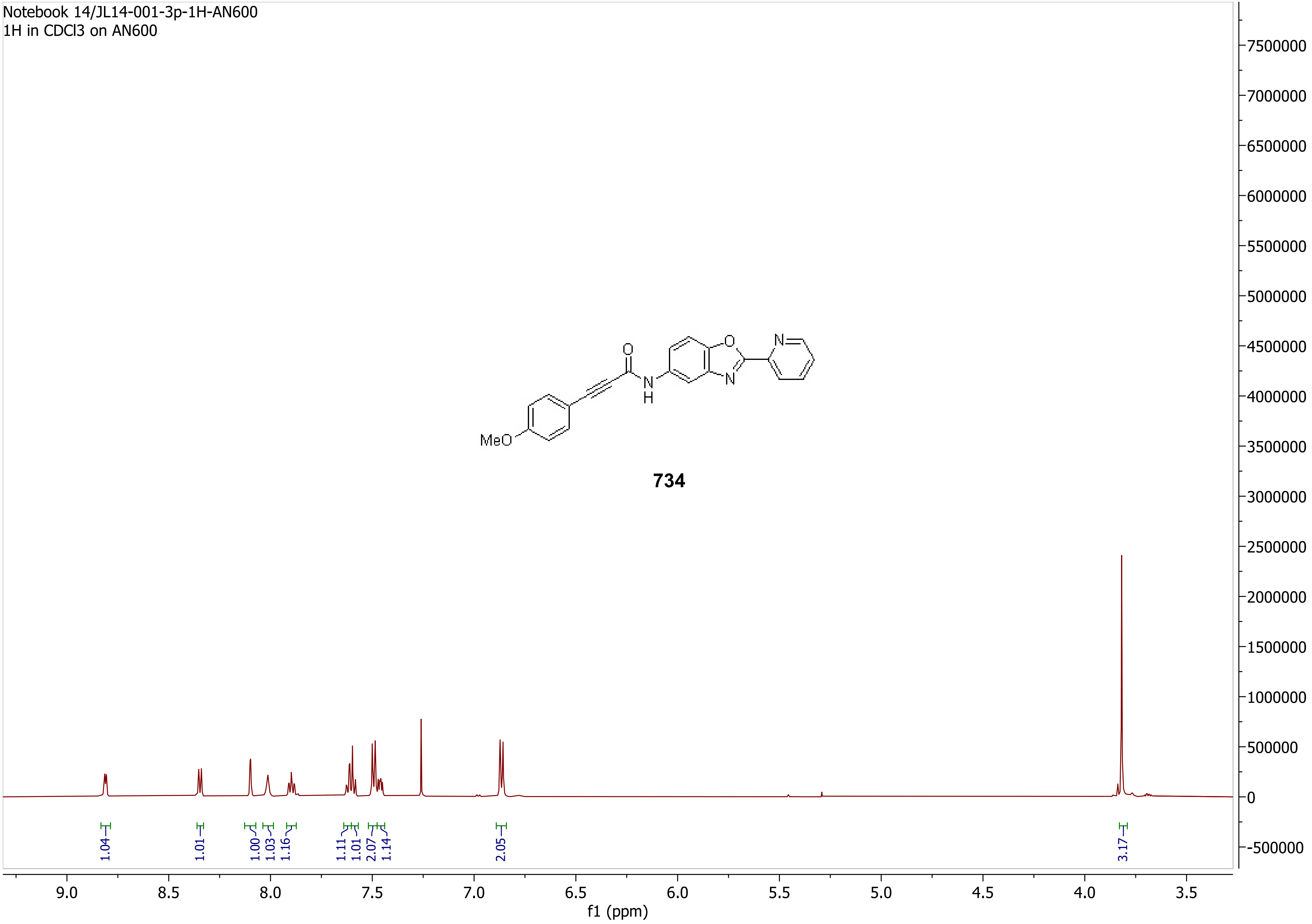
Notebook 14/JL14-001-3p-1H-AN600
1H in CDCl3 on AN600
MeO
O
N
H
O
N
N
734
1.04
1.01
1.00
1.03
1.16
1.11
1.01
2.07
1.14
2.05
3.17
9.0
8.5
8.0
7.5
7.0
6.5
6.0
5.5
5.0
4.5
4.0
3.5
f1 (ppm)
7500000
7000000
6500000
6000000
5500000
5000000
4500000
4000000
3500000
3000000
2500000
2000000
1500000
1000000
500000
0
-500000

Notebook 14/JL14-001-3p-13C-AN600
13C in CDCl3 on AN600

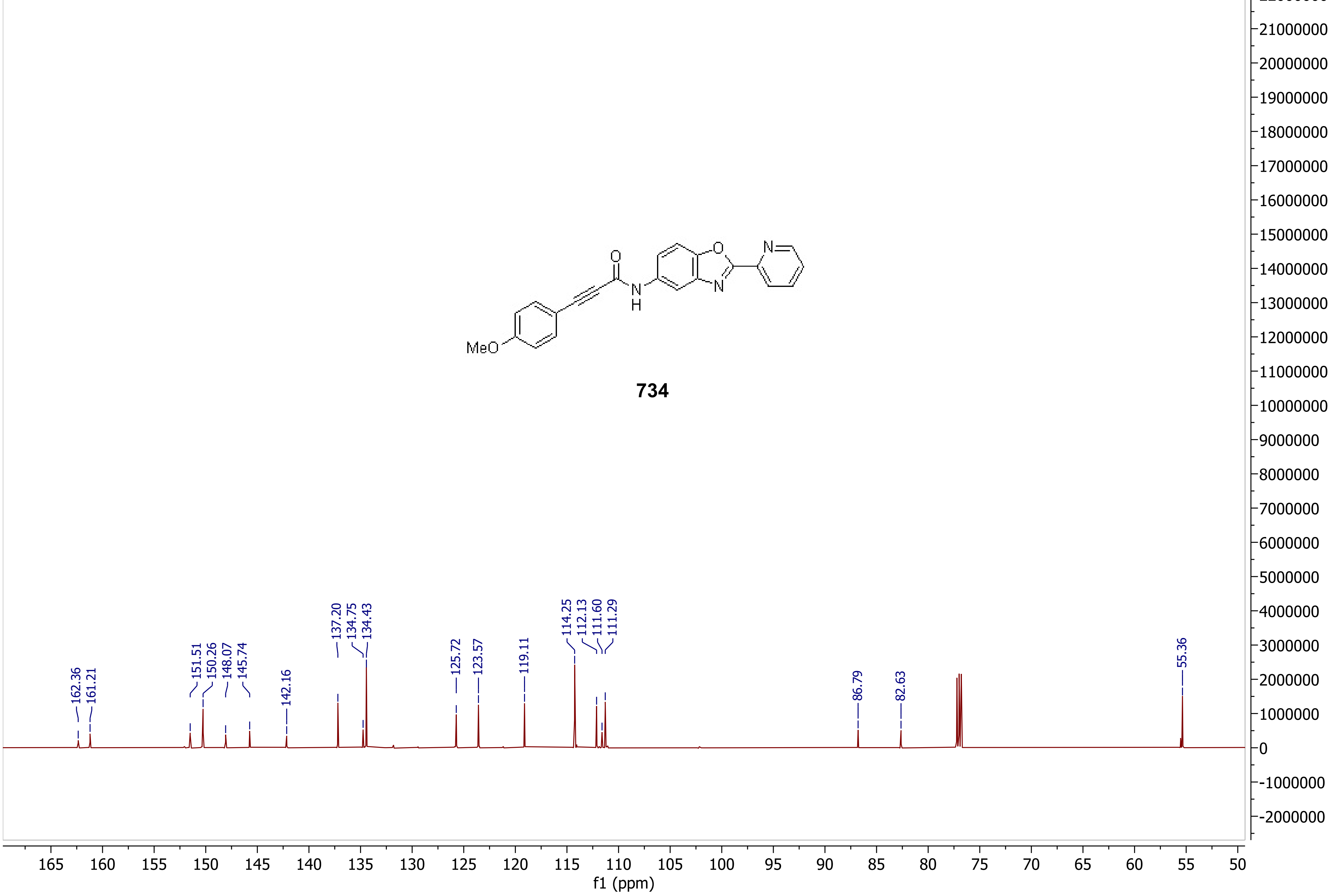

Notebook 14/JL14-002-1p-1H-AN600
off-white solid after 1) plug; 2) trituration, 1H in CDCl3 on AN600

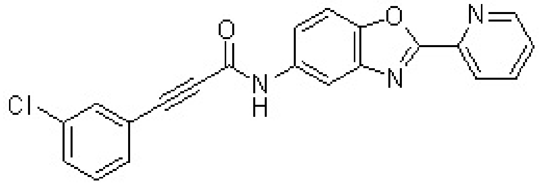

735

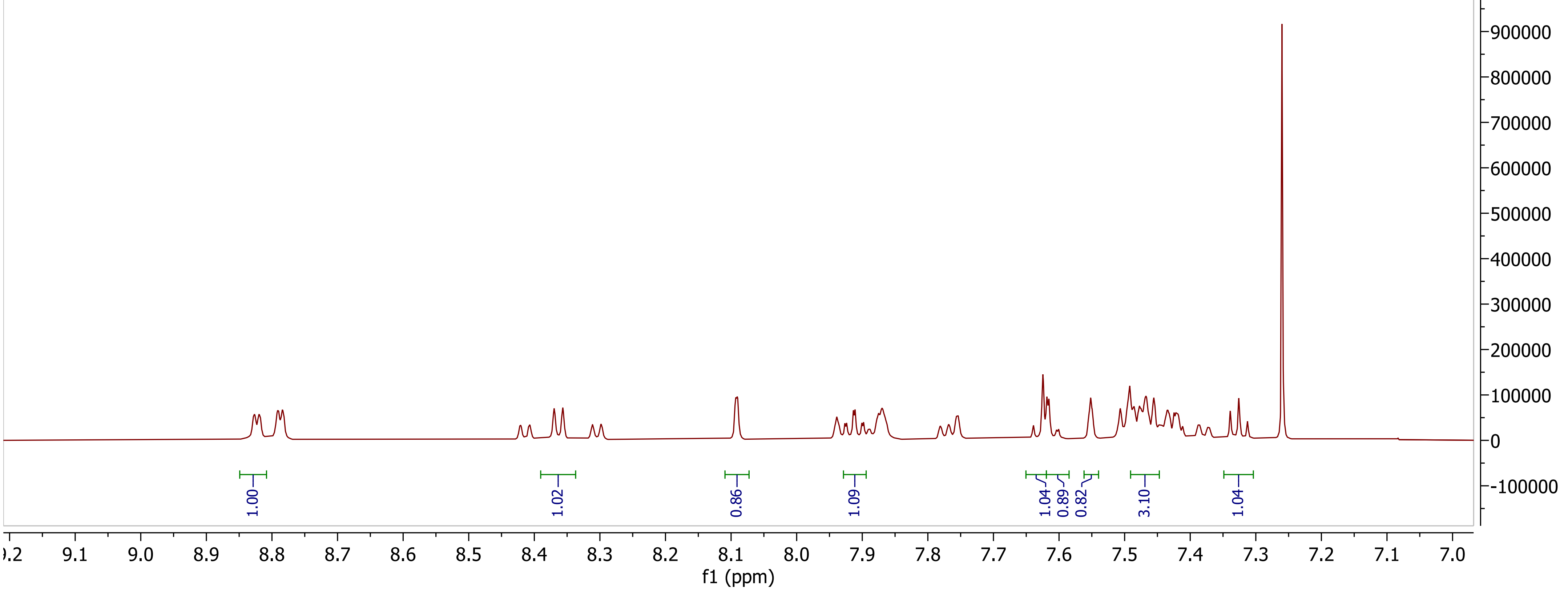

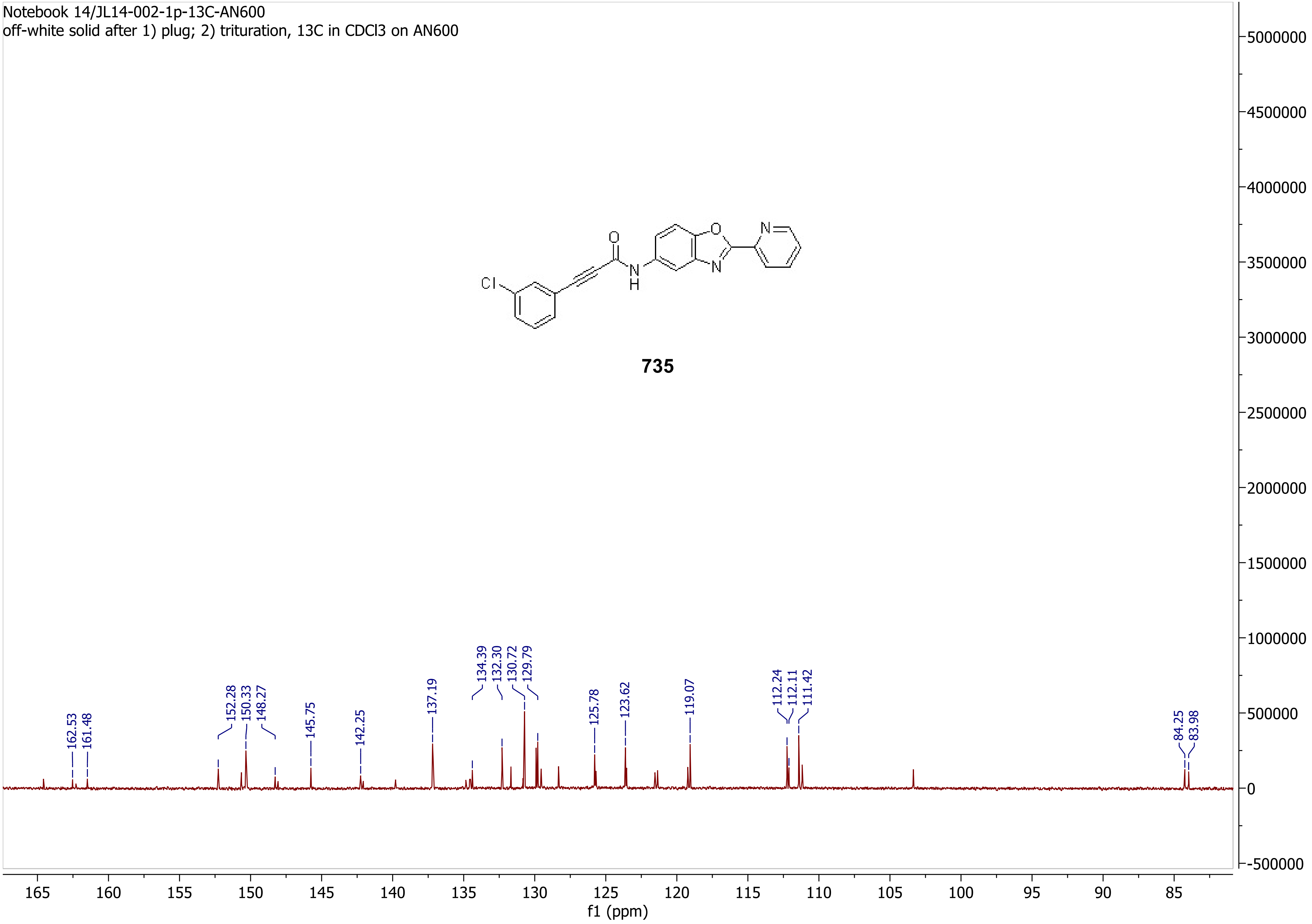
Notebook 14/JL14-002-1p-13C-AN600
off-white solid after 1) plug; 2) trituration, 13C in CDCl3 on AN600
735
162.53
161.48
152.28
150.33
148.27
145.75
142.25
137.19
134.39
132.30
130.72
129.79
125.78
123.62
119.07
112.24
112.11
111.42
84.25
83.98
f1 (ppm)

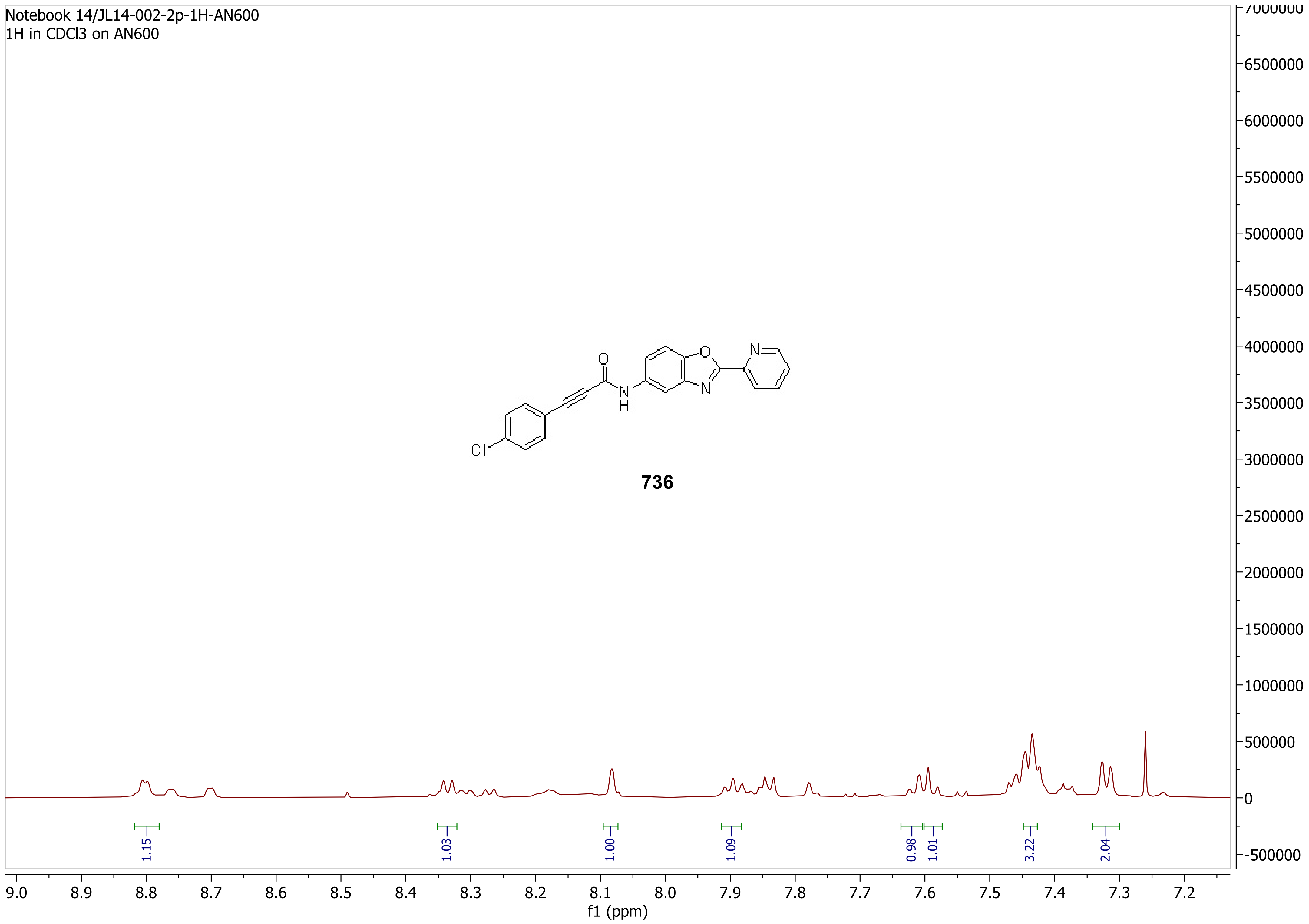
Notebook 14/JL14-002-2p-1H-AN600
1H in CDCl3 on AN600
O
N
H
N
O
N
Cl
736
1.15
1.03
1.00
1.09
0.98
1.01
3.22
2.04
7000000
6500000
6000000
5500000
5000000
4500000
4000000
3500000
3000000
2500000
2000000
1500000
1000000
500000
0
-500000
9.0
8.9
8.8
8.7
8.6
8.5
8.4
8.3
8.2
8.1
8.0
7.9
7.8
7.7
7.6
7.5
7.4
7.3
7.2
f1 (ppm)

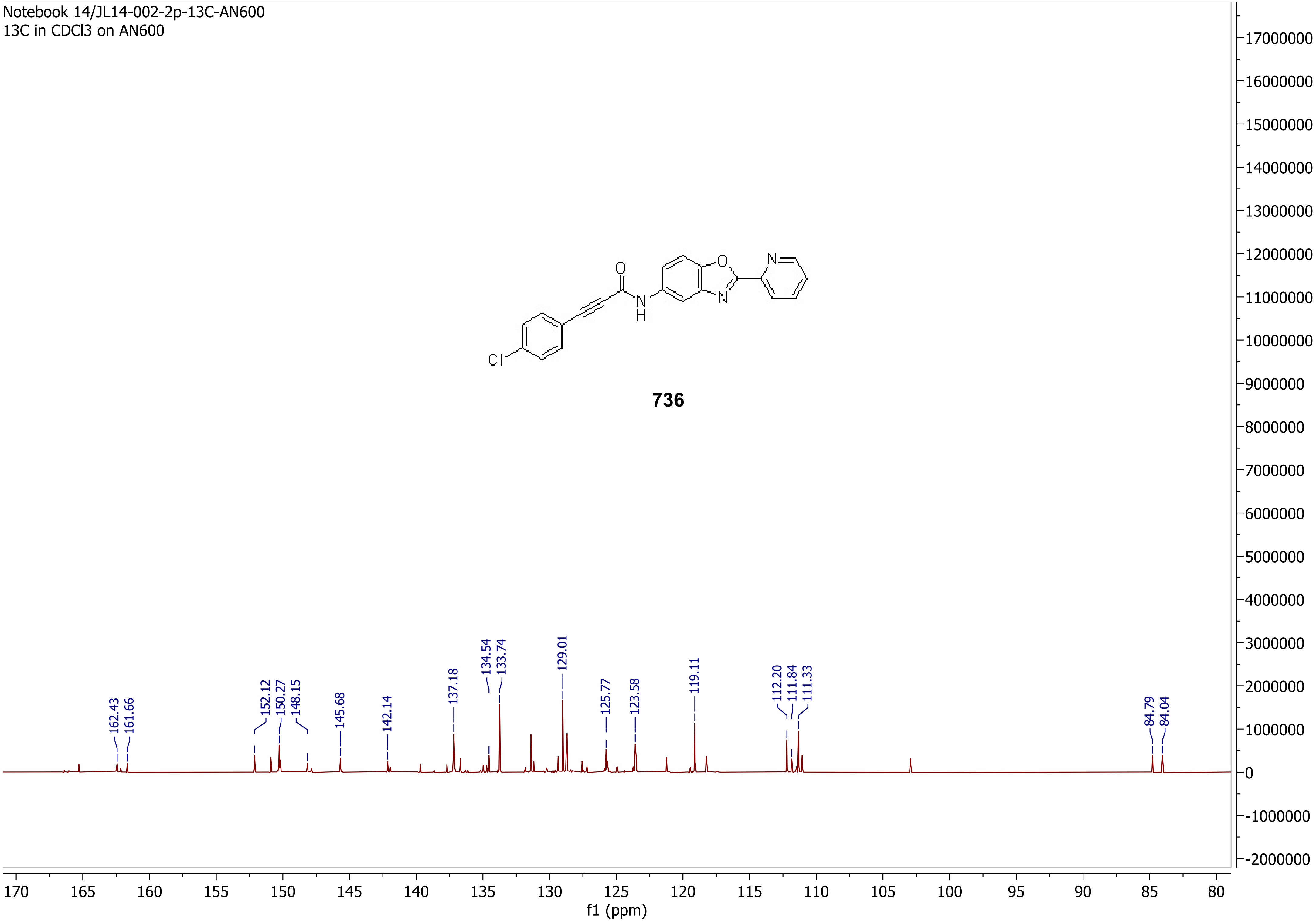
Notebook 14/JL14-002-2p-13C-AN600
13C in CDCl3 on AN600
O
N
H
O
N
N
Cl
736
162.43
161.66
152.12
150.27
148.15
145.68
142.14
137.18
134.54
133.74
129.01
125.77
123.58
119.11
112.20
111.84
111.33
84.79
84.04
f1 (ppm)
170 165 160 155 150 145 140 135 130 125 120 115 110 105 100 95 90 85 80
17000000
16000000
15000000
14000000
13000000
12000000
11000000
10000000
9000000
8000000
7000000
6000000
5000000
4000000
3000000
2000000
1000000
0
-1000000
-2000000

Notebook 14/JL14-004-1p-1H-AN600-1
1H in CDCl3 on AN600

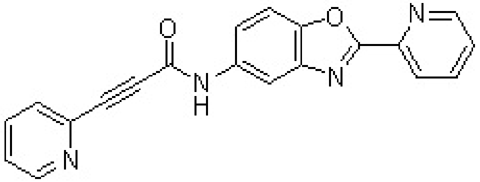

737

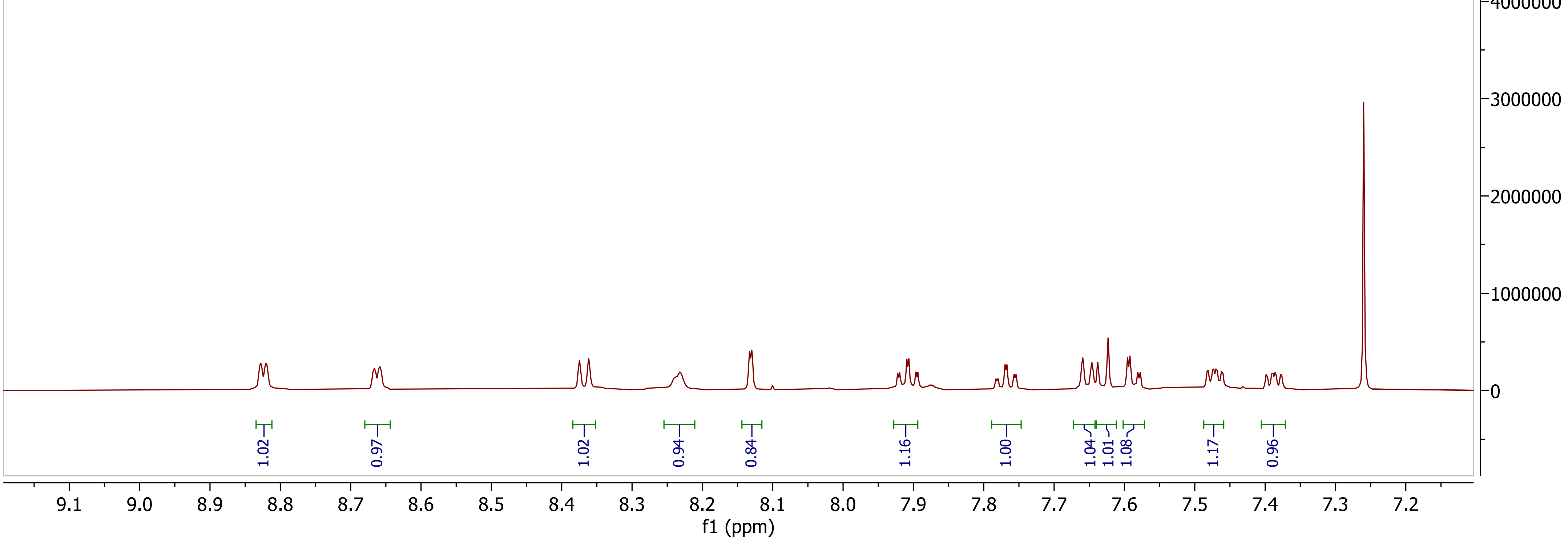

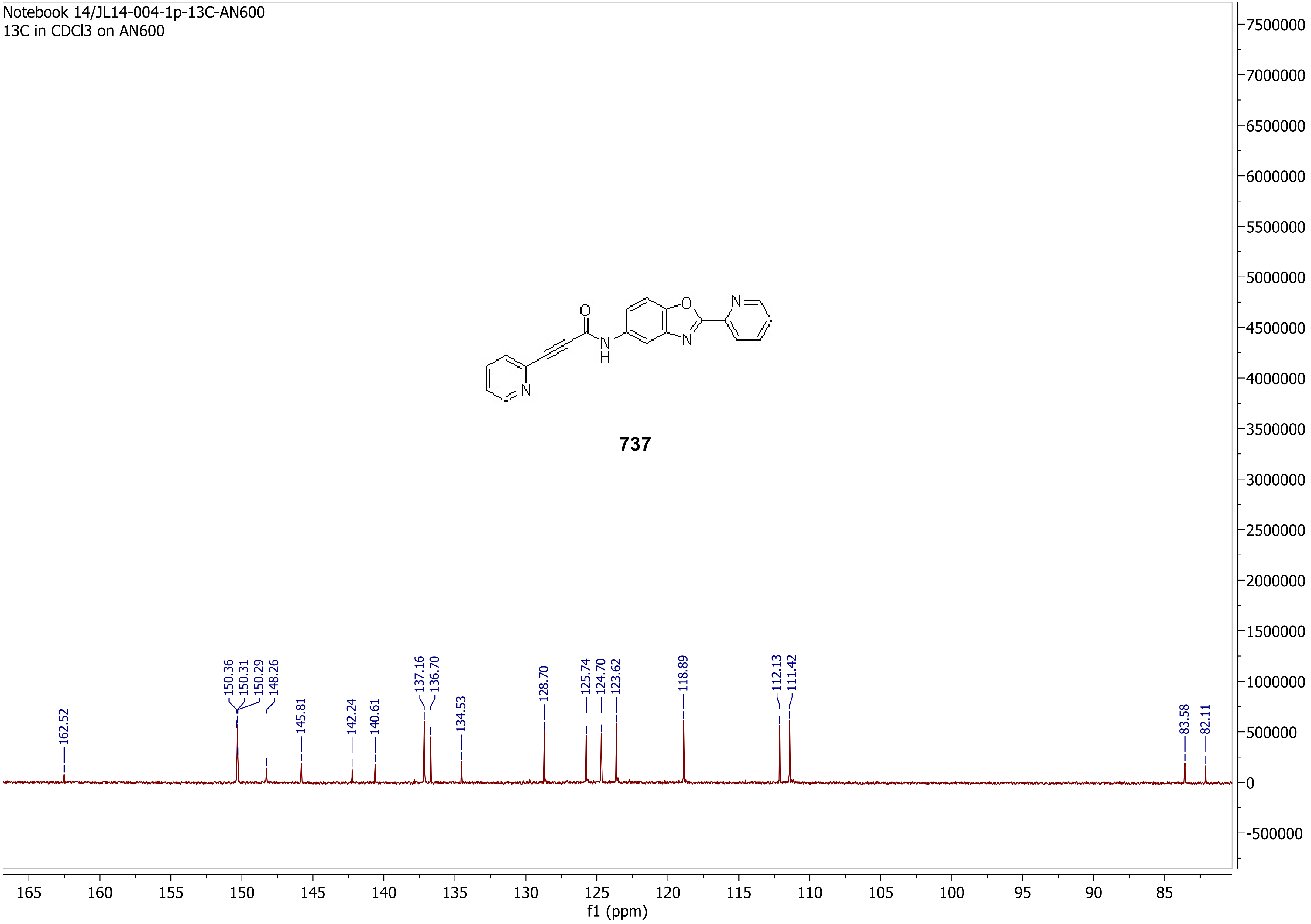

Notebook 14/JL14-004-1p-13C-AN600
13C in CDCl3 on AN600
737
162.52
150.36
150.31
150.29
148.26
145.81
142.24
140.61
137.16
136.70
134.53
128.70
125.74
124.70
123.62
118.89
112.13
111.42
83.58
82.11
f1 (ppm)

Notebook 14/JL14-004-2p-1H-AN600
white ppt, poor solubility, 1H in d4-MeOH w. 12D CDCl3 w. heat, on AN600

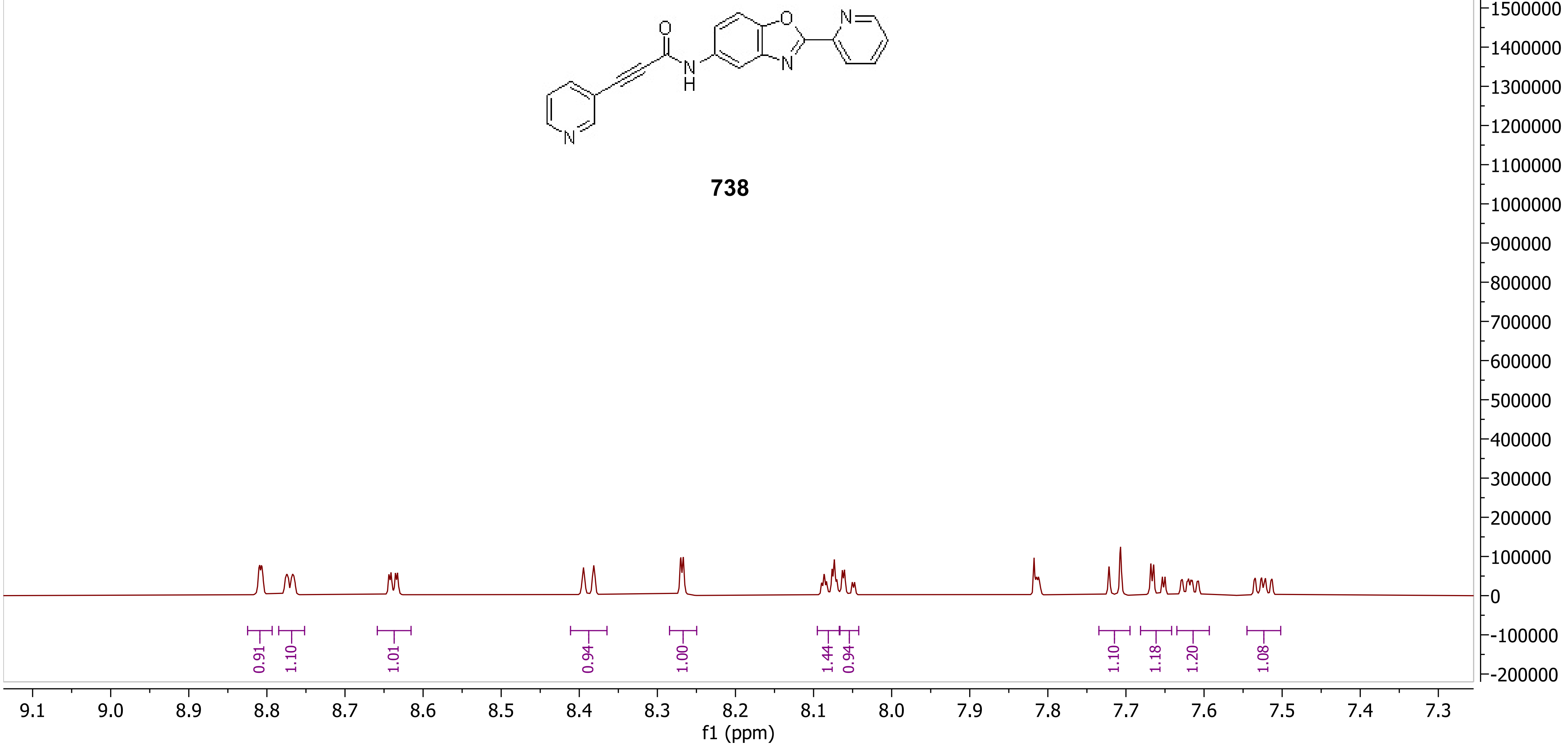

738

Notebook 14/JL14-004-2p-13C-AN600
white ppt, poor solubility, 13C in d4-MeOH w. 12D CDCl3 w. heat, on AN600

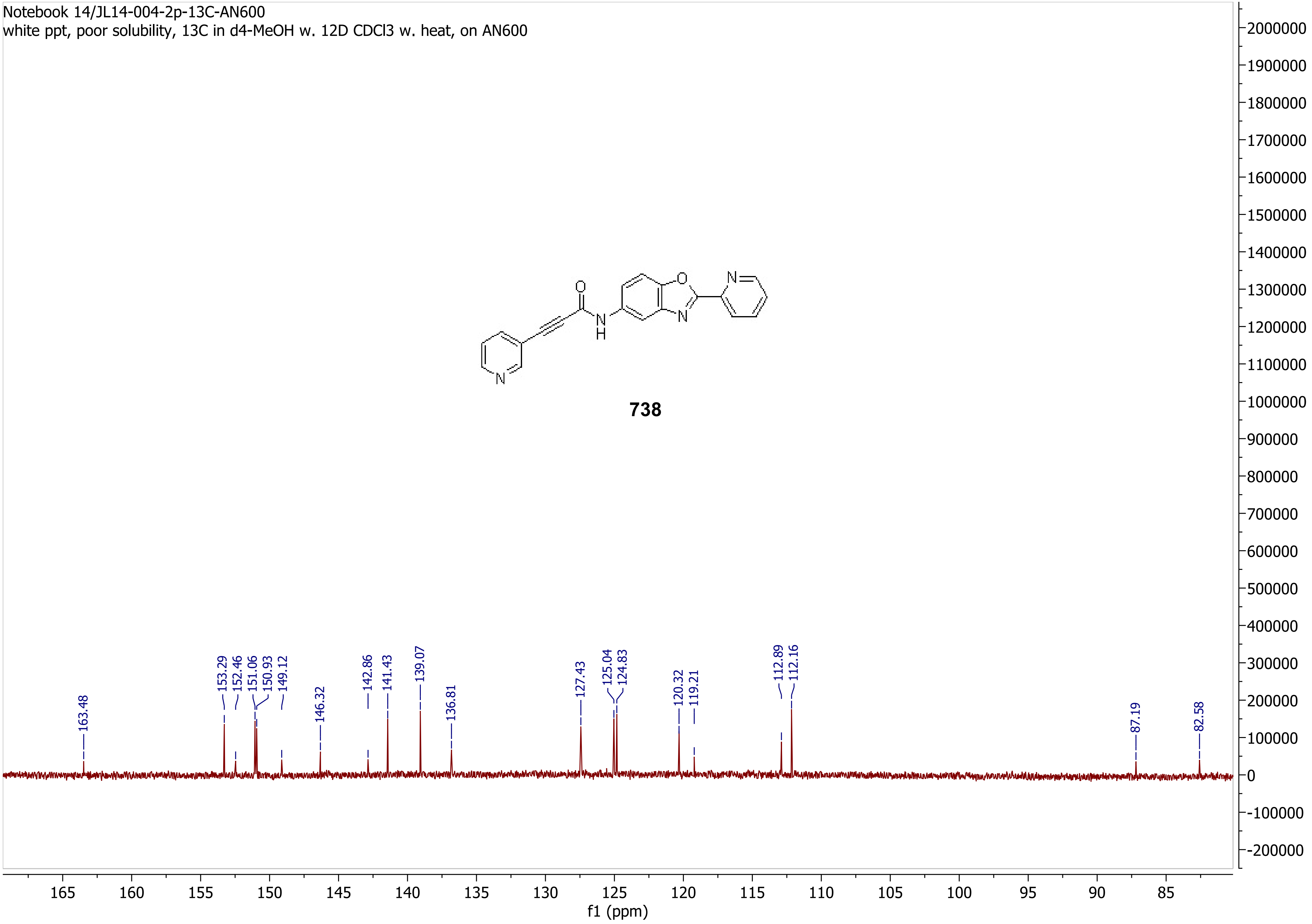

738

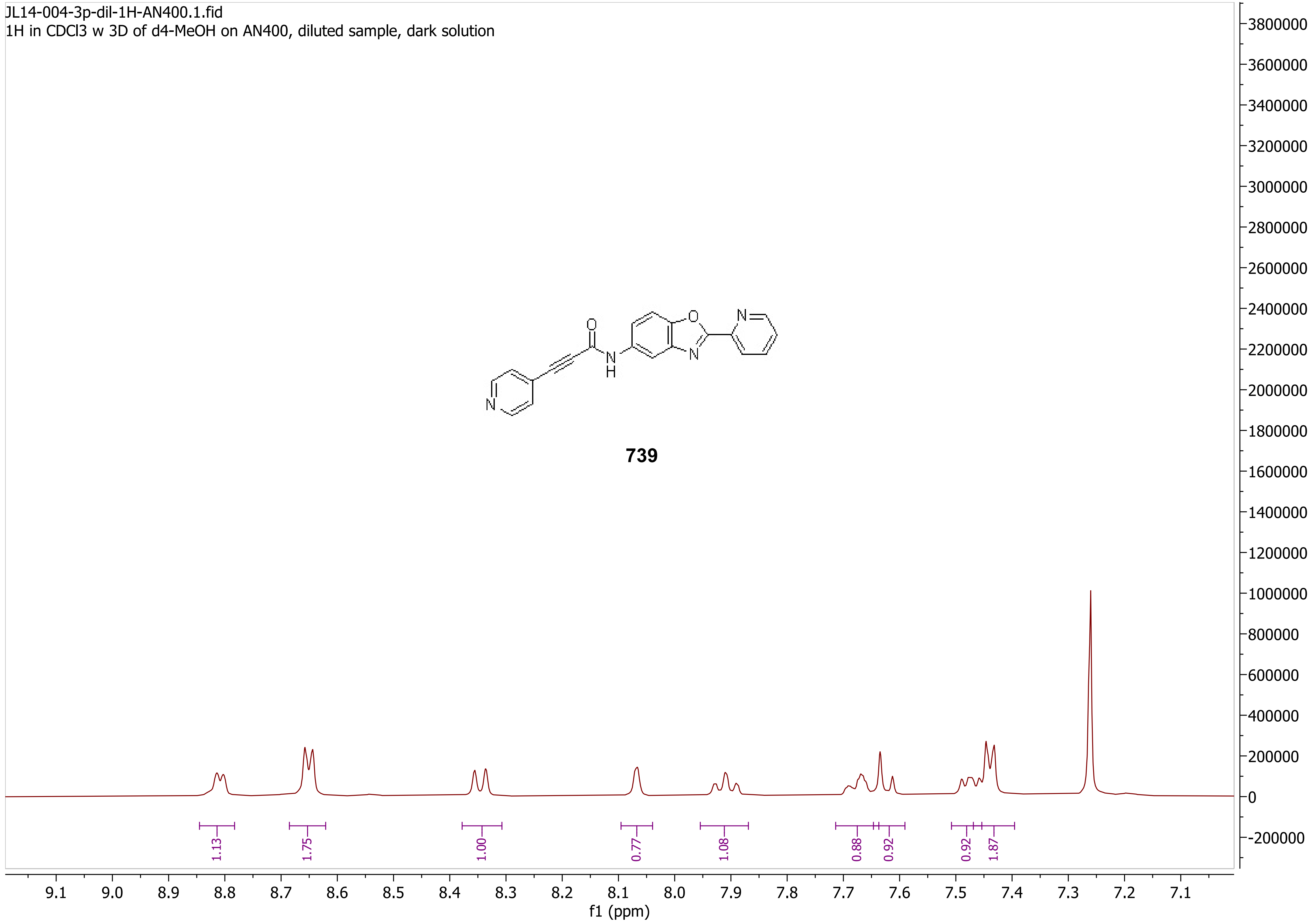
JL14-004-3p-dil-1H-AN400.1.fid
1H in CDCl3 w 3D of d4-MeOH on AN400, diluted sample, dark solution
O
N
H
O
N
N
N
739
1.13
1.75
1.00
0.77
1.08
0.88
0.92
0.92
1.87
9.1
9.0
8.9
8.8
8.7
8.6
8.5
8.4
8.3
8.2
8.1
8.0
7.9
7.8
7.7
7.6
7.5
7.4
7.3
7.2
7.1
f1 (ppm)
3800000
3600000
3400000
3200000
3000000
2800000
2600000
2400000
2200000
2000000
1800000
1600000
1400000
1200000
1000000
800000
600000
400000
200000
0
-200000

Notebook 14/JL14-004-3p-b-13C-AN600
light-brown solution quickly turned to greenish, 13C in CDCl3 w 8D d4-MeOH on AN600

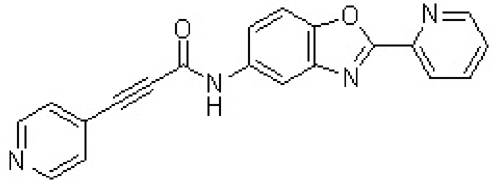

739

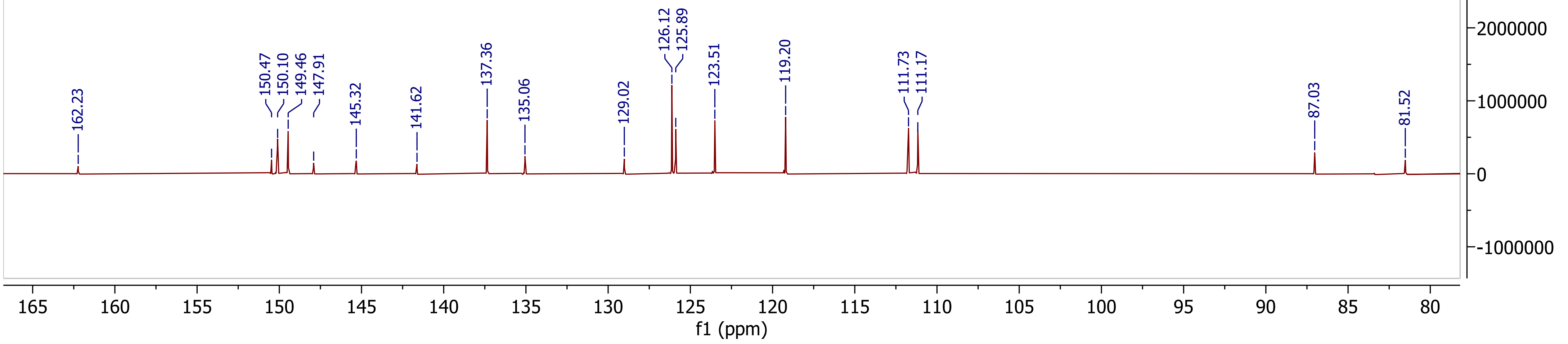

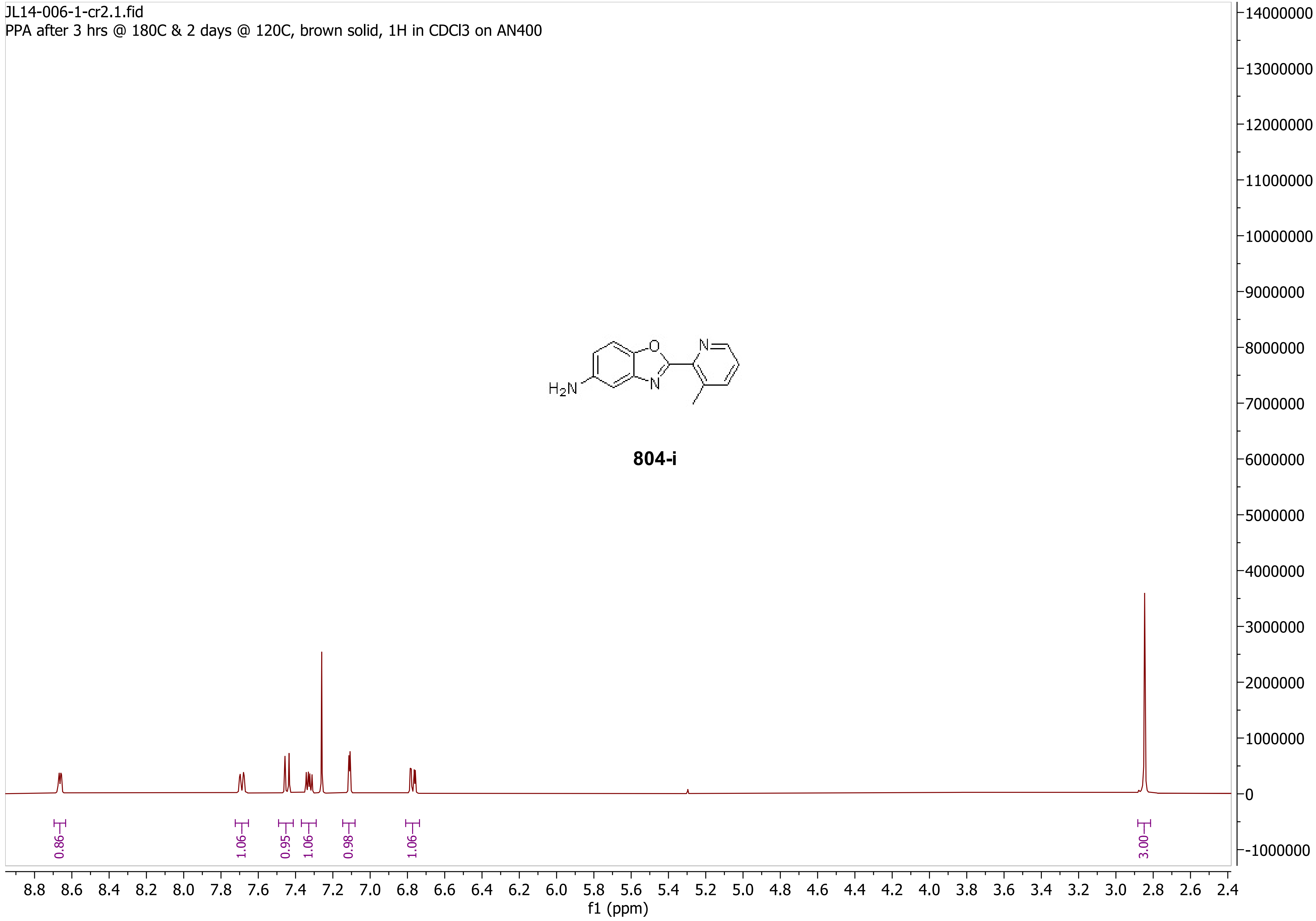

JL14-006-1-cr2.1.fid
PPA after 3 hrs @ 180C & 2 days @ 120C, brown solid, 1H in CDCl3 on AN400
H2N
O
N
N
804-i
0.86
1.06
0.95
1.06
0.98
1.06
3.00
f1 (ppm)
8.8 8.6 8.4 8.2 8.0 7.8 7.6 7.4 7.2 7.0 6.8 6.6 6.4 6.2 6.0 5.8 5.6 5.4 5.2 5.0 4.8 4.6 4.4 4.2 4.0 3.8 3.6 3.4 3.2 3.0 2.8 2.6 2.4
14000000
13000000
12000000
11000000
10000000
9000000
8000000
7000000
6000000
5000000
4000000
3000000
2000000
1000000
0
-1000000

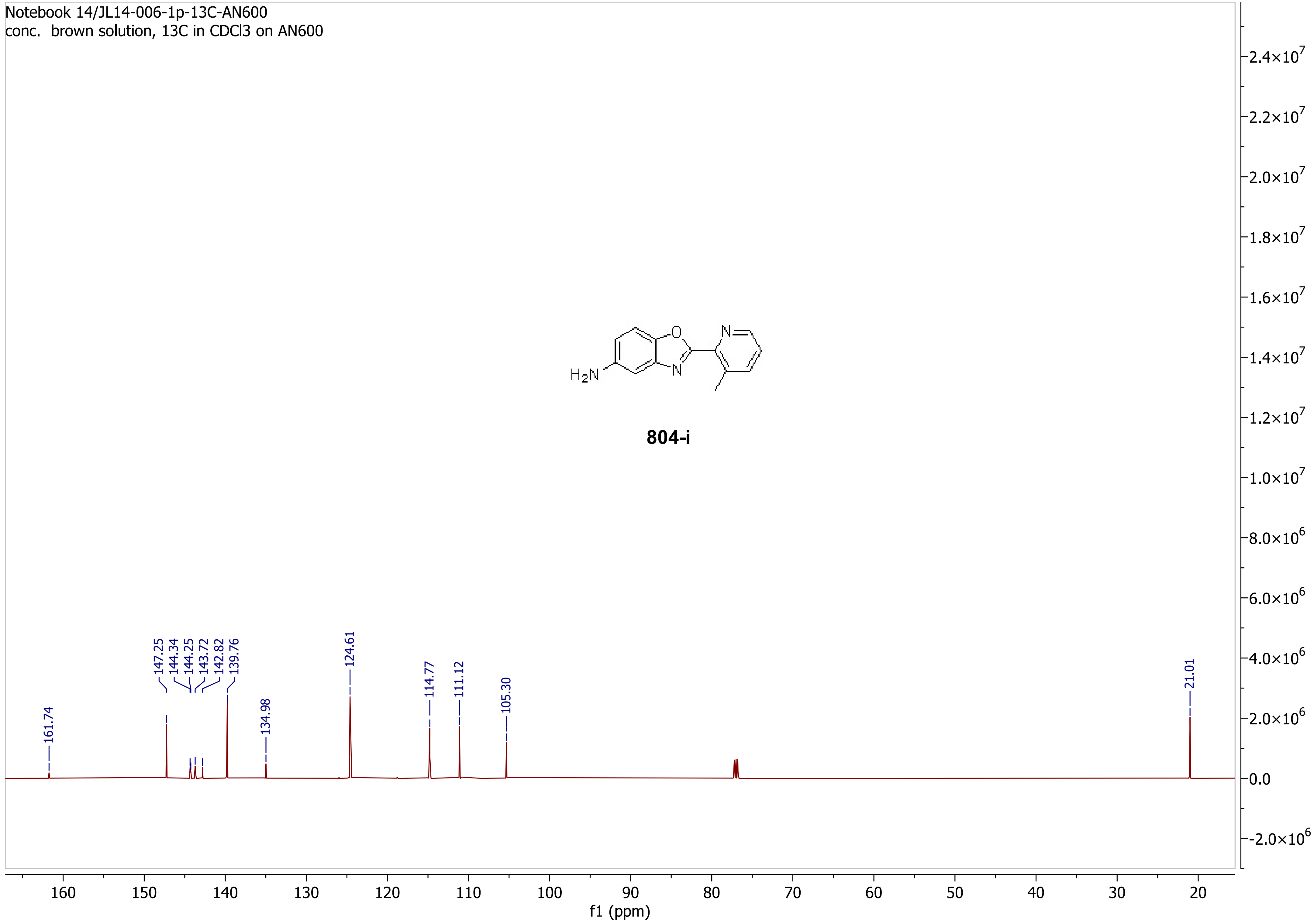
Notebook 14/JL14-006-1p-13C-AN600
conc. brown solution, 13C in CDCl3 on AN600
804-i
161.74
147.25
144.34
144.25
143.72
142.82
139.76
134.98
124.61
114.77
111.12
105.30
21.01
f1 (ppm)
160
150
140
130
120
110
100
90
80
70
60
50
40
30
20
2.4×10^7
2.2×10^7
2.0×10^7
1.8×10^7
1.6×10^7
1.4×10^7
1.2×10^7
1.0×10^7
8.0×10^6
6.0×10^6
4.0×10^6
2.0×10^6
0.0
-2.0×10^6

Notebook 14/JL14-009-1p-1H-AN600
yellow solid after 5% MeOH-Cm plug & DCM-hex wash, tube 3, 1H in d4-MeOH w. 15D of CDCl3 on AN600

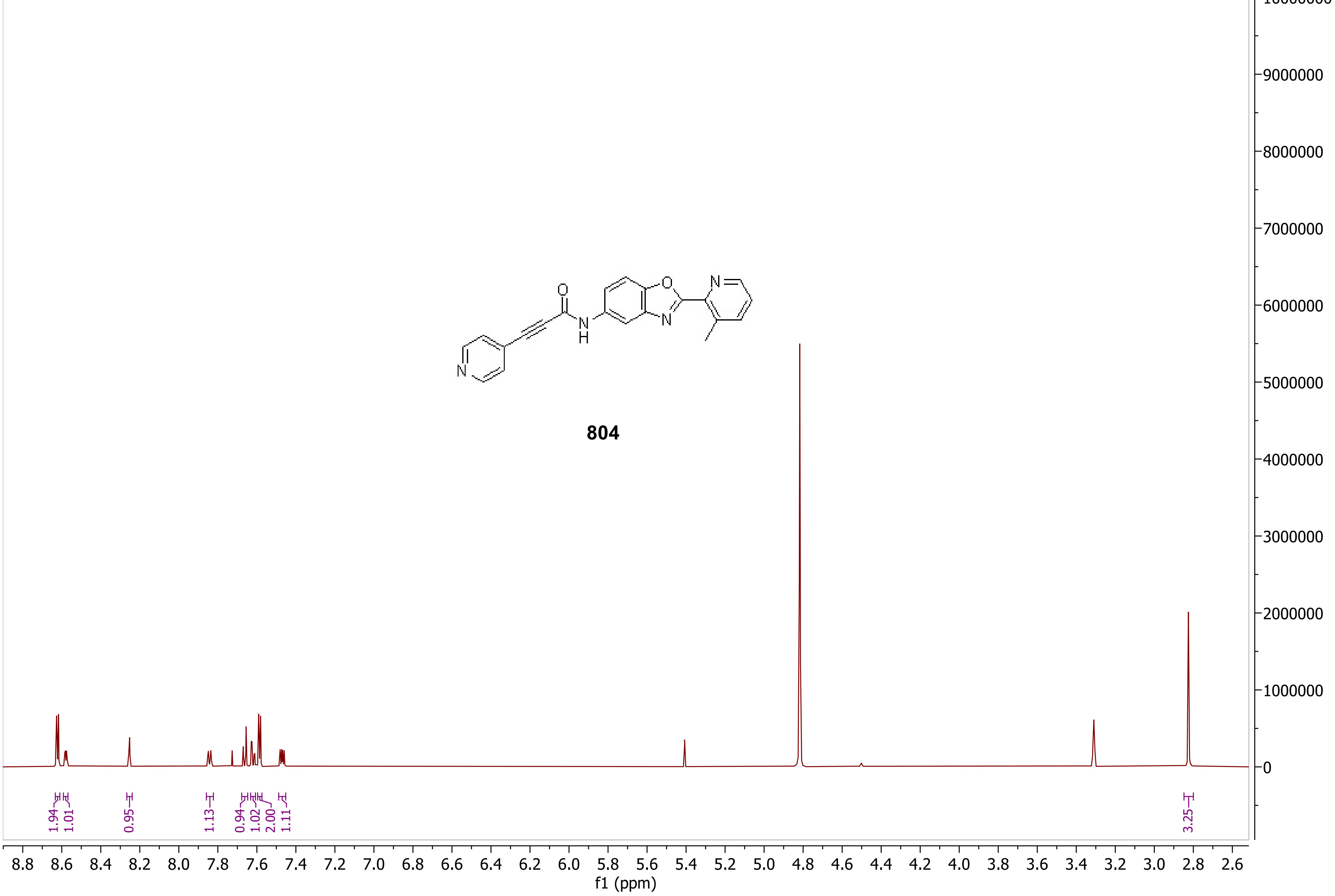

Notebook 14/JL14-009-1p-13C-AN600
yellow solid after 5% MeOH-Cm plug & DCM-hex wash, tube 3, 13C in d4-MeOH w. 15D of CDCl3 on AN600

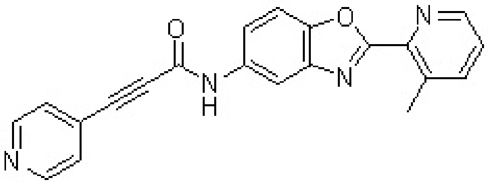

804

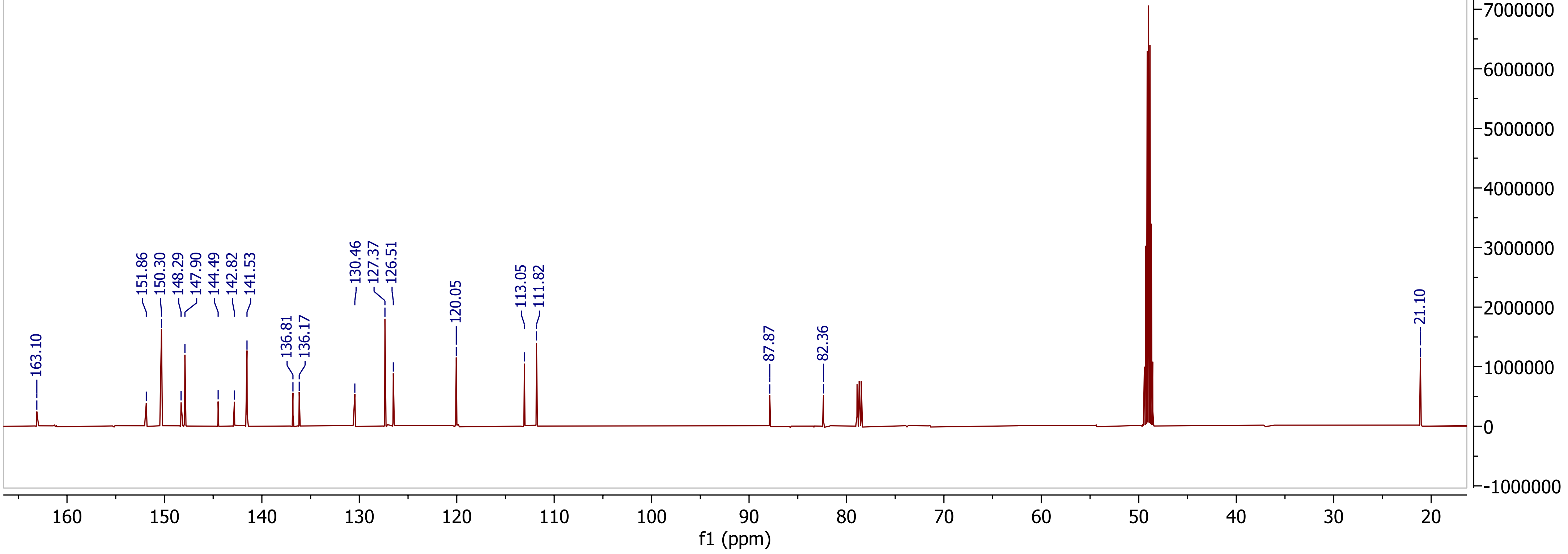

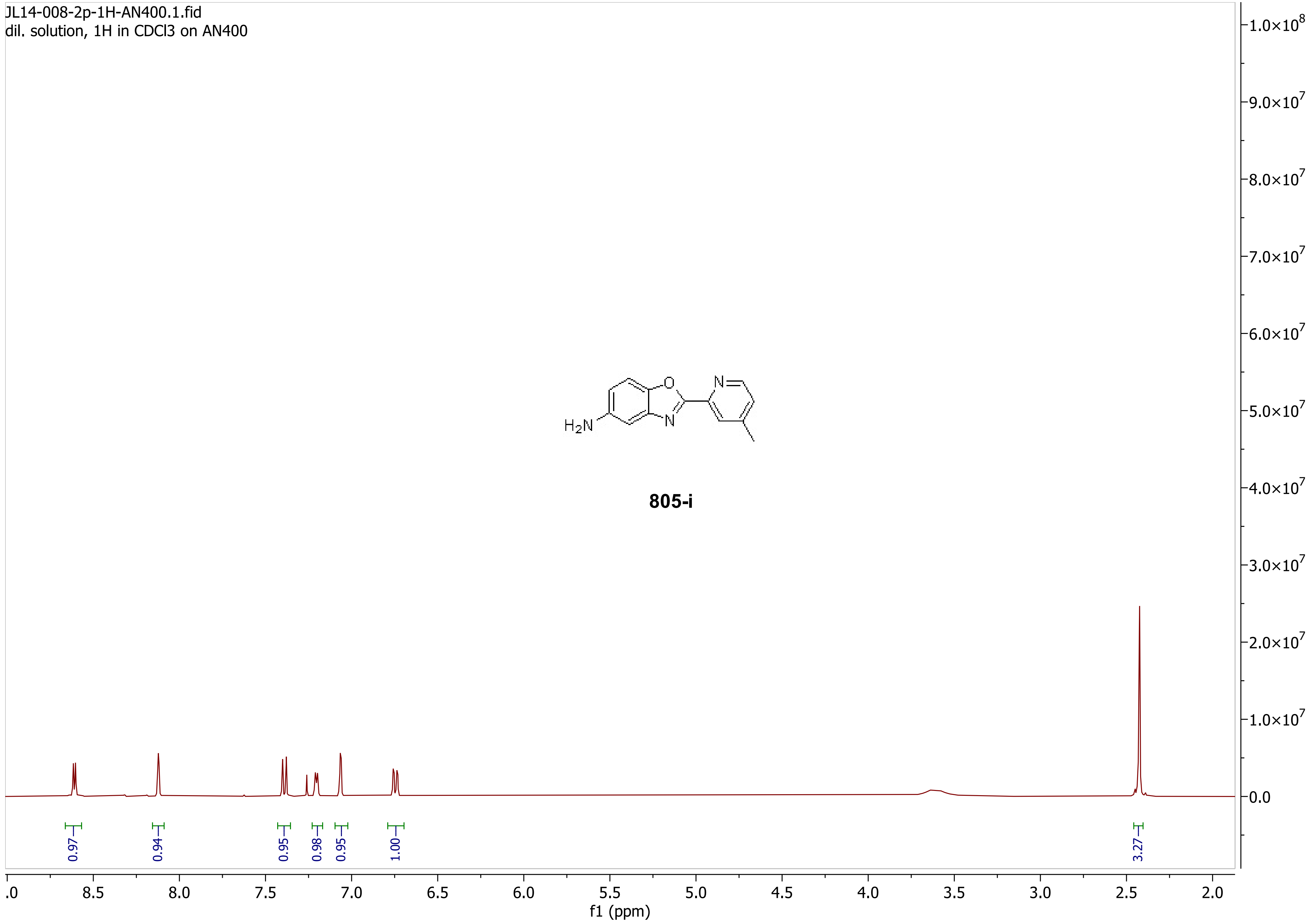

JL14-008-2p-1H-AN400.1.fid
dil. solution, 1H in CDCl3 on AN400
805-i
0.97
0.94
0.95
0.98
0.95
1.00
3.27
f1 (ppm)

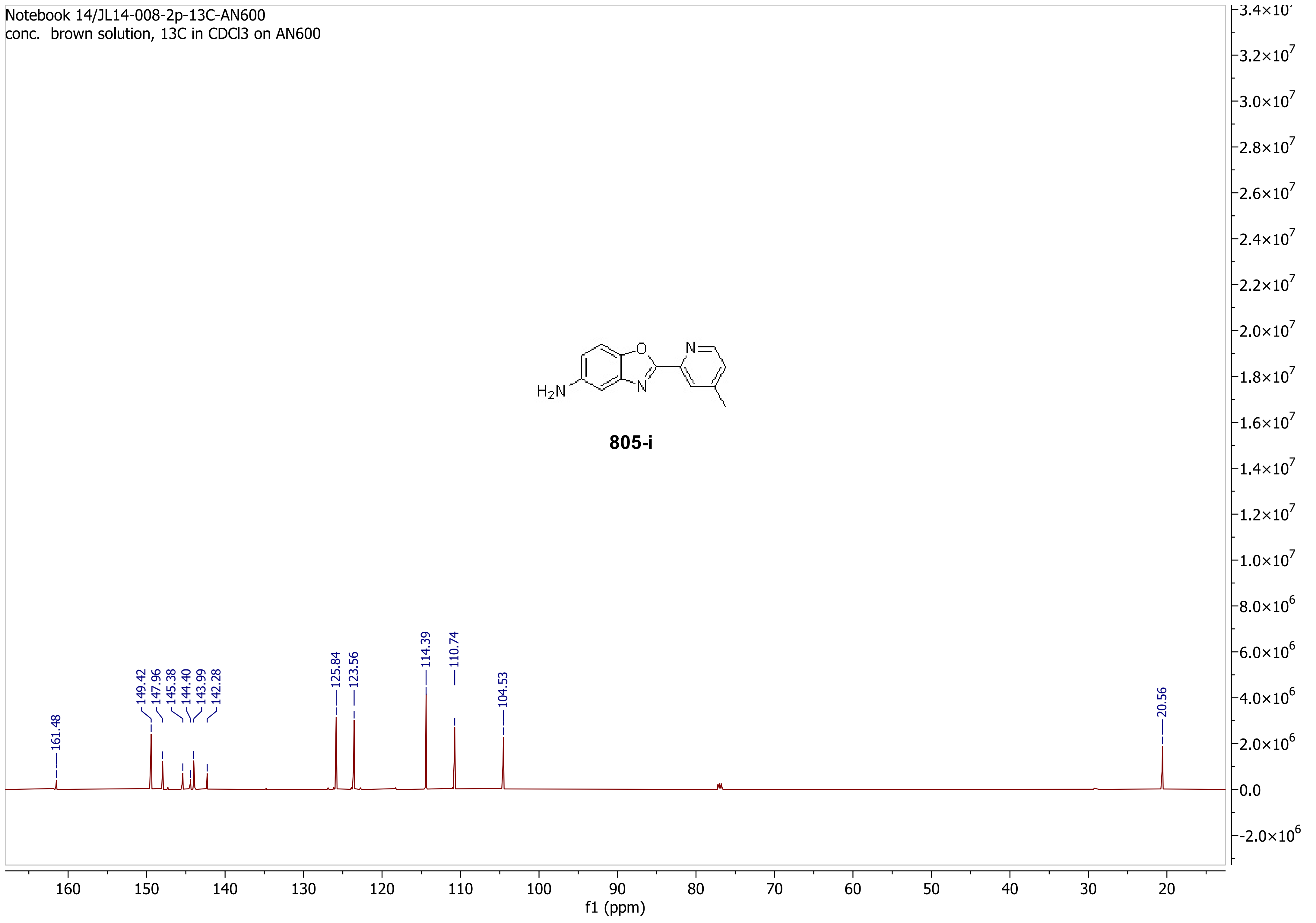

Notebook 14/JL14-008-2p-13C-AN600
conc. brown solution, 13C in CDCl3 on AN600
O
N
N
H2N
805-i
161.48
149.42
147.96
145.38
144.40
143.99
142.28
125.84
123.56
114.39
110.74
104.53
20.56
f1 (ppm)

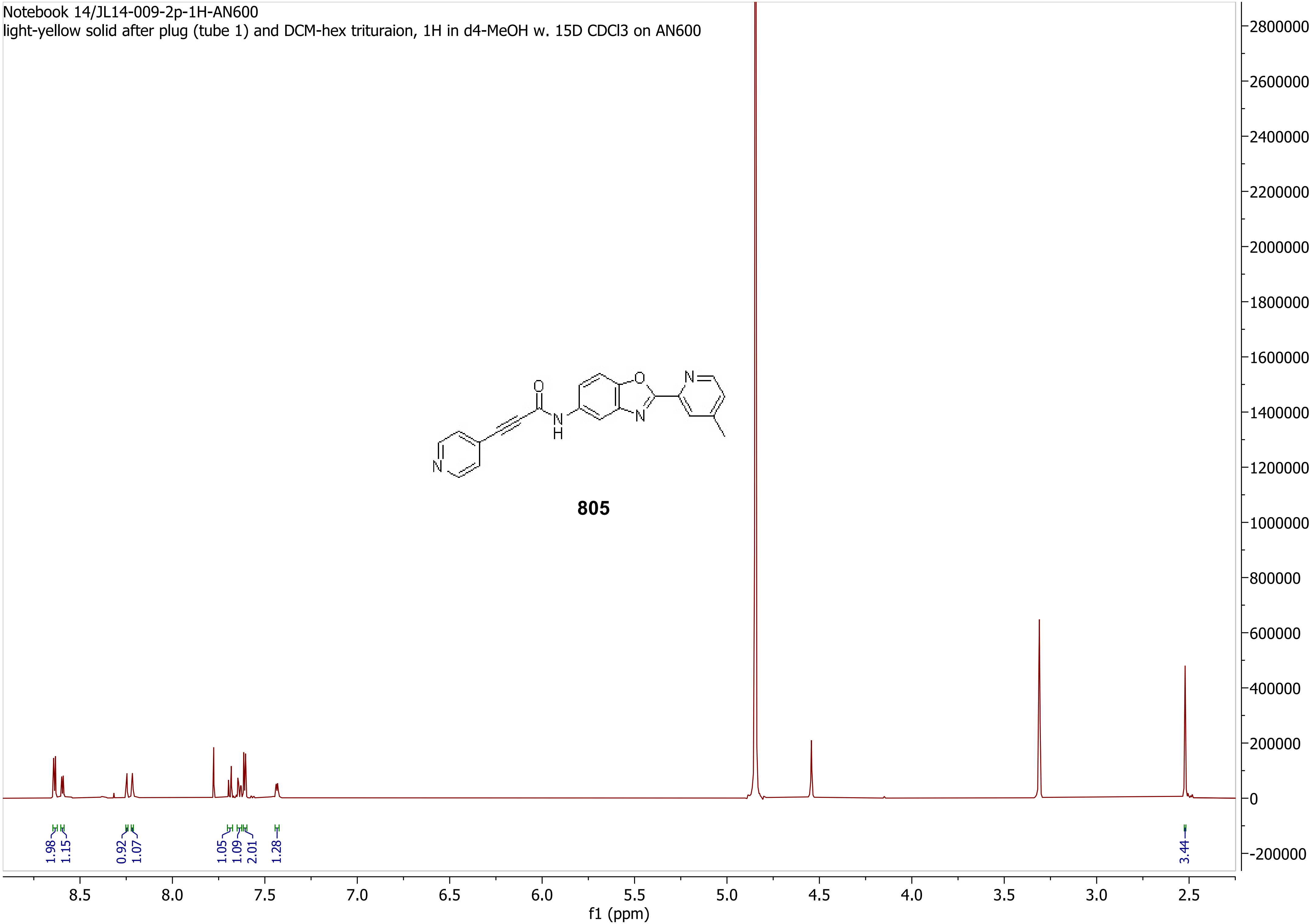
Notebook 14/JL14-009-2p-1H-AN600
light-yellow solid after plug (tube 1) and DCM-hex trituraion, 1H in d4-MeOH w. 15D CDCl3 on AN600
805
1.98
1.15
0.92
1.07
1.05
1.09
2.01
1.28
3.44
8.5
8.0
7.5
7.0
6.5
6.0
5.5
5.0
4.5
4.0
3.5
3.0
2.5
f1 (ppm)
2800000
2600000
2400000
2200000
2000000
1800000
1600000
1400000
1200000
1000000
800000
600000
400000
200000
0
-200000

Notebook 14/JL14-009-2p-13C-AN600
light-yellow solid after plug (tube 1) and DCM-hex trituraion, 1H in d4-MeOH w. 15D CDCl3 on AN600

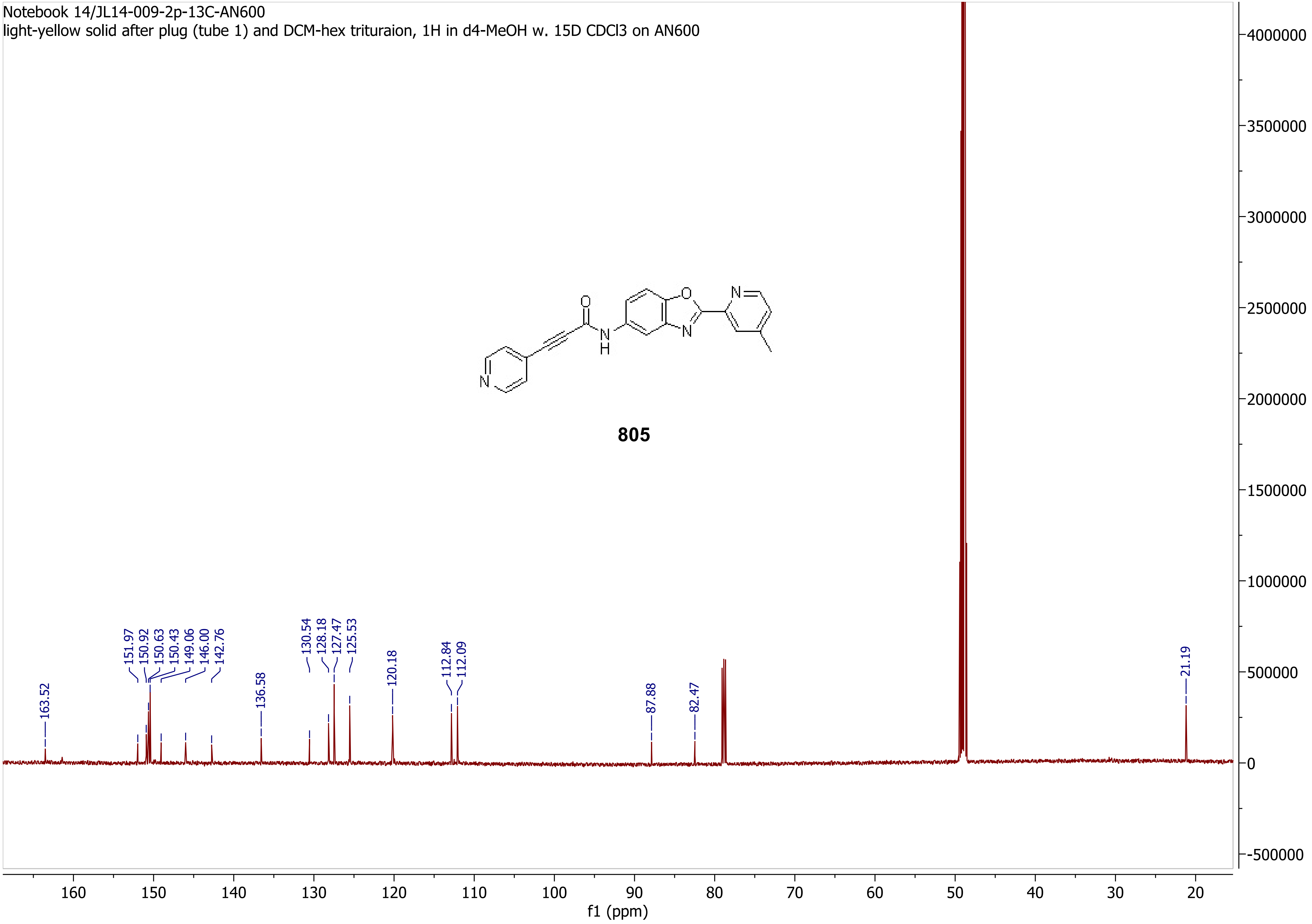

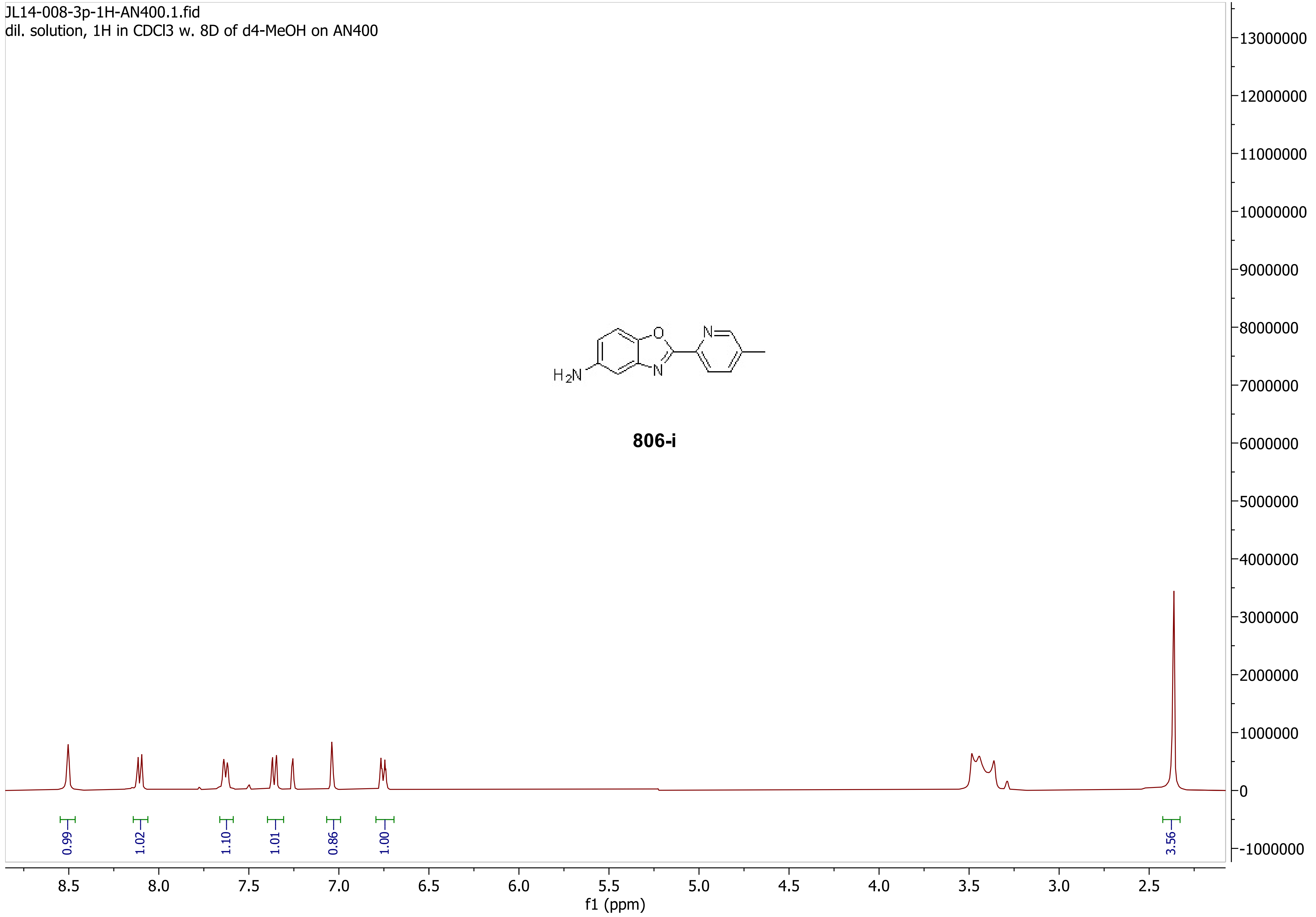

JL14-008-3p-1H-AN400.1.fid
dil. solution, 1H in CDCl3 w. 8D of d4-MeOH on AN400
H2N
O
N
N
806-i
0.99
1.02
1.10
1.01
0.86
1.00
3.56
8.5
8.0
7.5
7.0
6.5
6.0
5.5
5.0
4.5
4.0
3.5
3.0
2.5
f1 (ppm)
13000000
12000000
11000000
10000000
9000000
8000000
7000000
6000000
5000000
4000000
3000000
2000000
1000000
0
-1000000

Notebook 14/JL14-008-3p-13C-AN600
conc. yellow solution, 13C in CDCl3 w. 5D of d4-MeOH on AN600

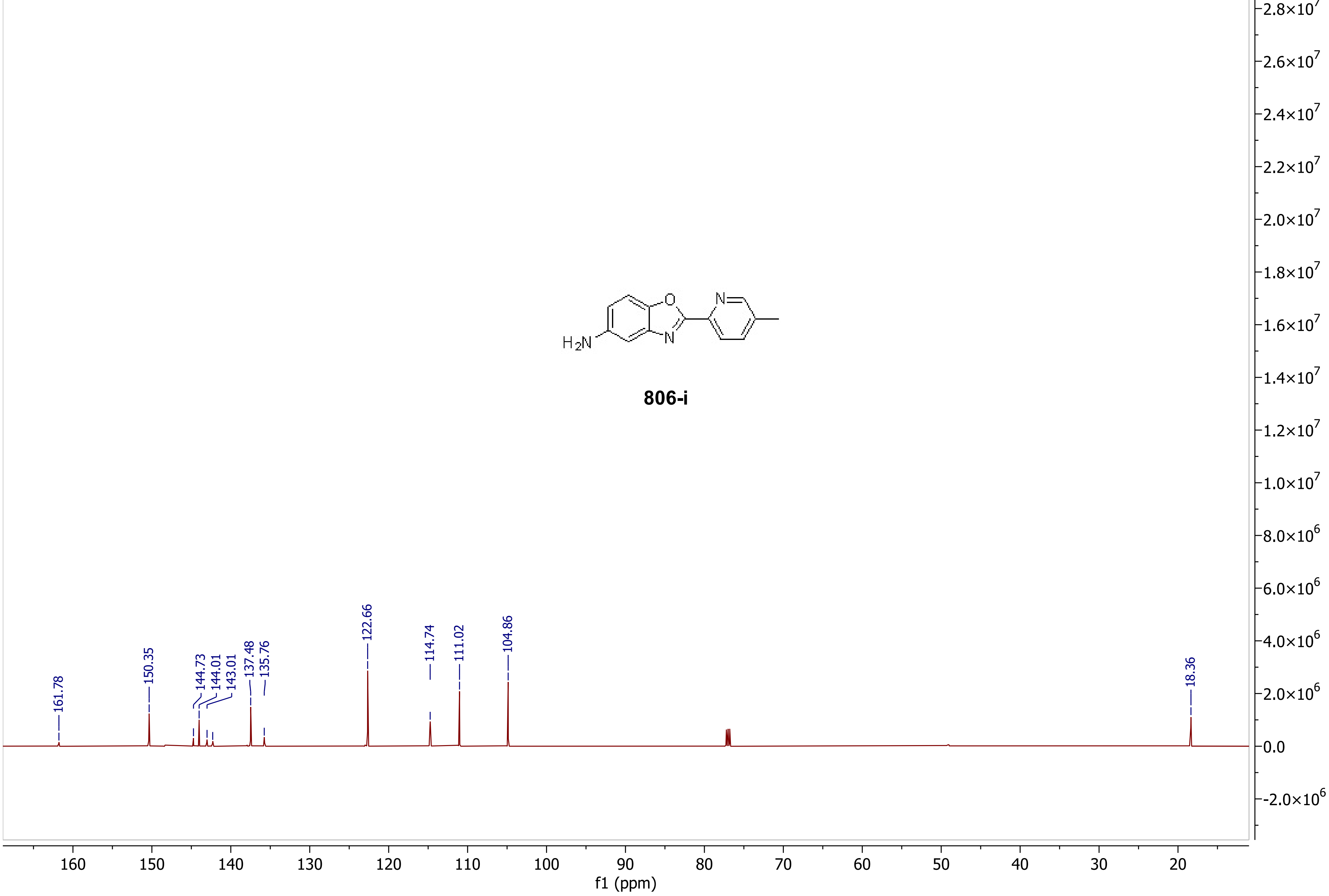

Notebook 14/JL14-009-3p-1H-AN600
light-yellow solid after 5% MeOH-DCM plug, tube 3, 1H in d4-MeOH w. 15D CDCl3 on AN600

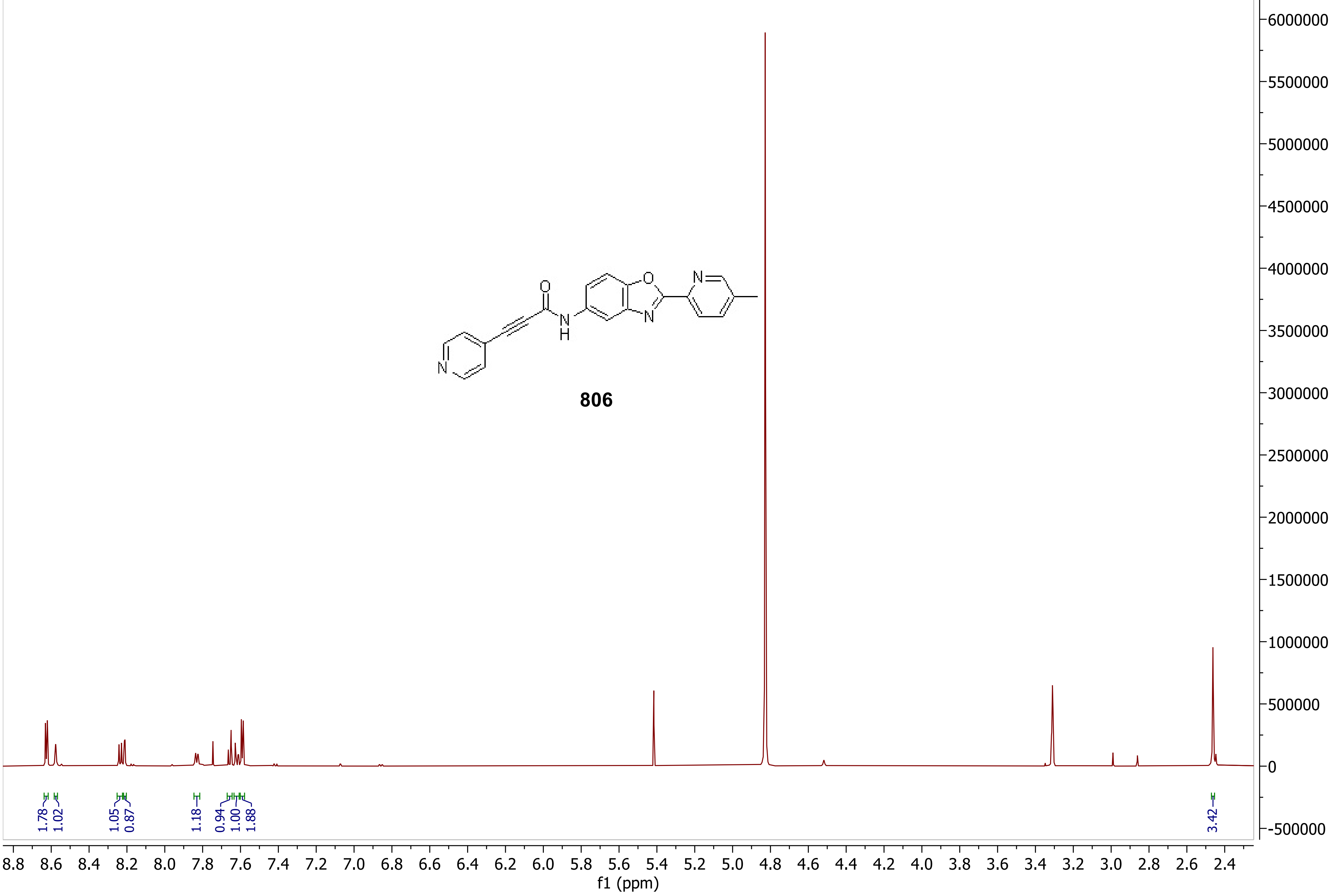

Notebook 14/JL14-009-3p-13C-AN600
light-yellow solid after 5% MeOH-DCM plug, tube 3, 13C in d4-MeOH w. 15D CDCl3 on AN600

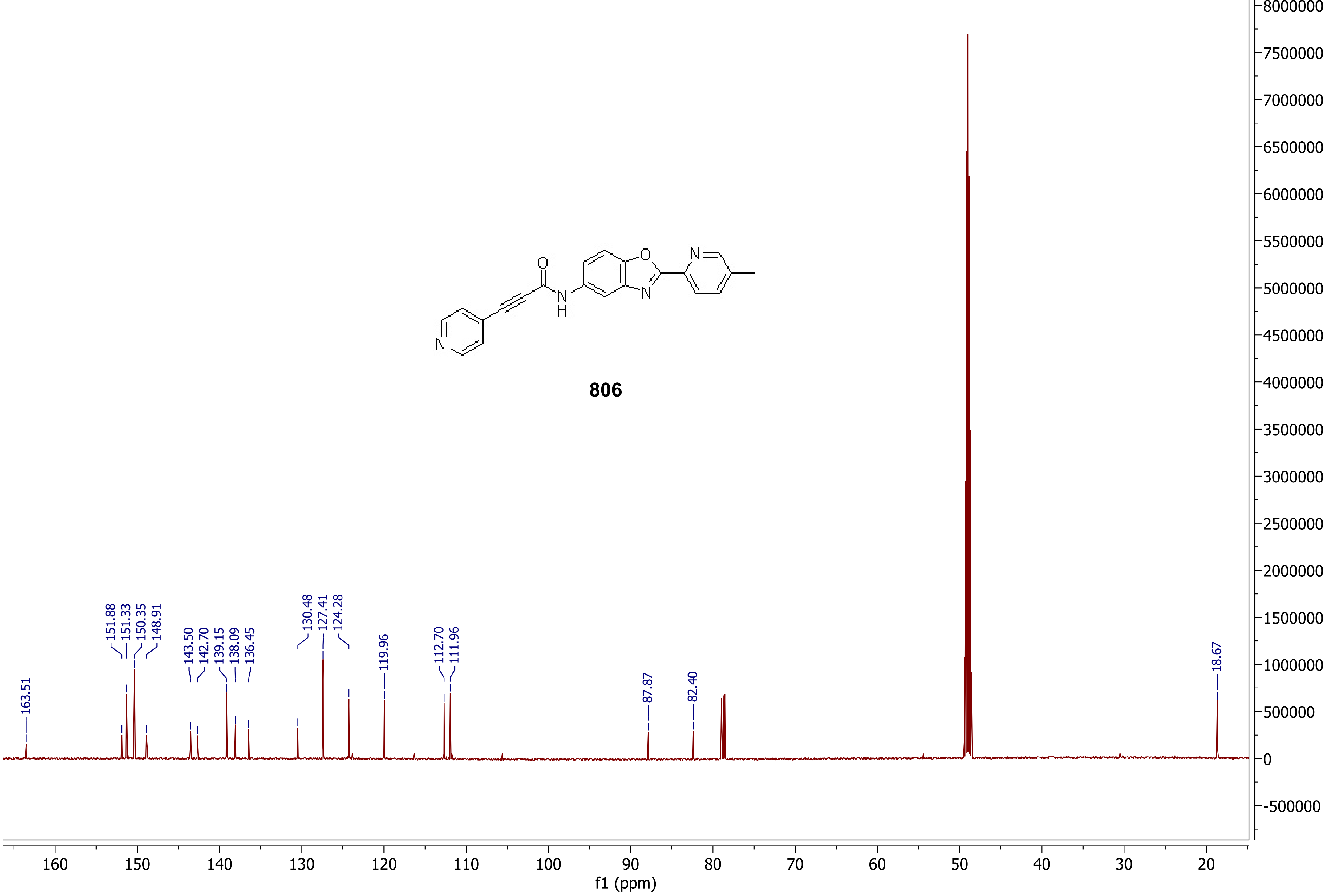

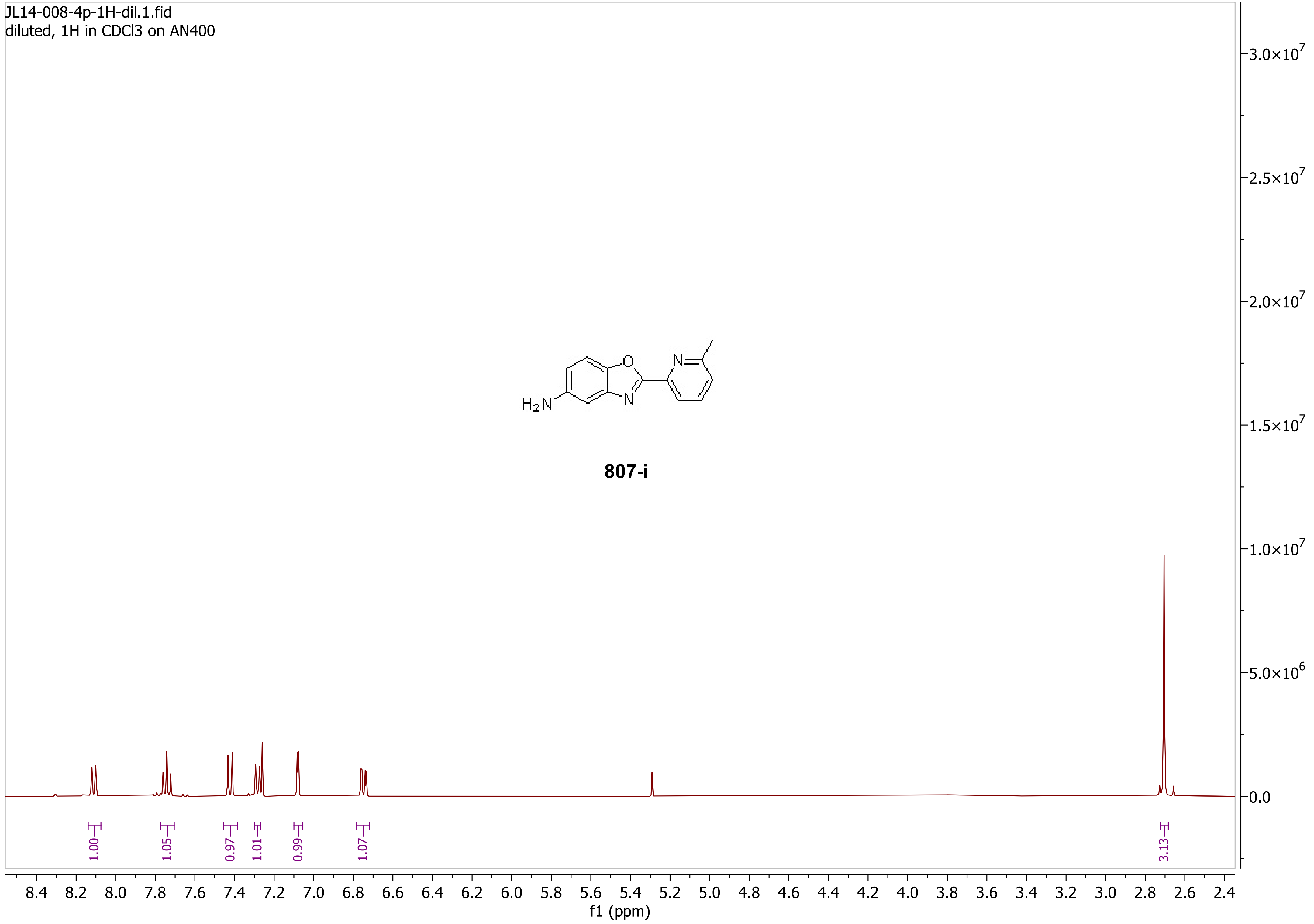
JL14-008-4p-1H-dil.1.fid
diluted, 1H in CDCl3 on AN400
H_2N
O
N
N
807-i
1.00
1.05
0.97
1.01
0.99
1.07
3.13
8.4 8.2 8.0 7.8 7.6 7.4 7.2 7.0 6.8 6.6 6.4 6.2 6.0 5.8 5.6 5.4 5.2 5.0 4.8 4.6 4.4 4.2 4.0 3.8 3.6 3.4 3.2 3.0 2.8 2.6 2.4
f1 (ppm)
3.0×10^7
2.5×10^7
2.0×10^7
1.5×10^7
1.0×10^7
5.0×10^6
0.0

JL14-008-4p-13C-AN400.3.fid
orange solid after extraction & 5% MeOH-DCM plug, 13C in CDCl3 on AN400

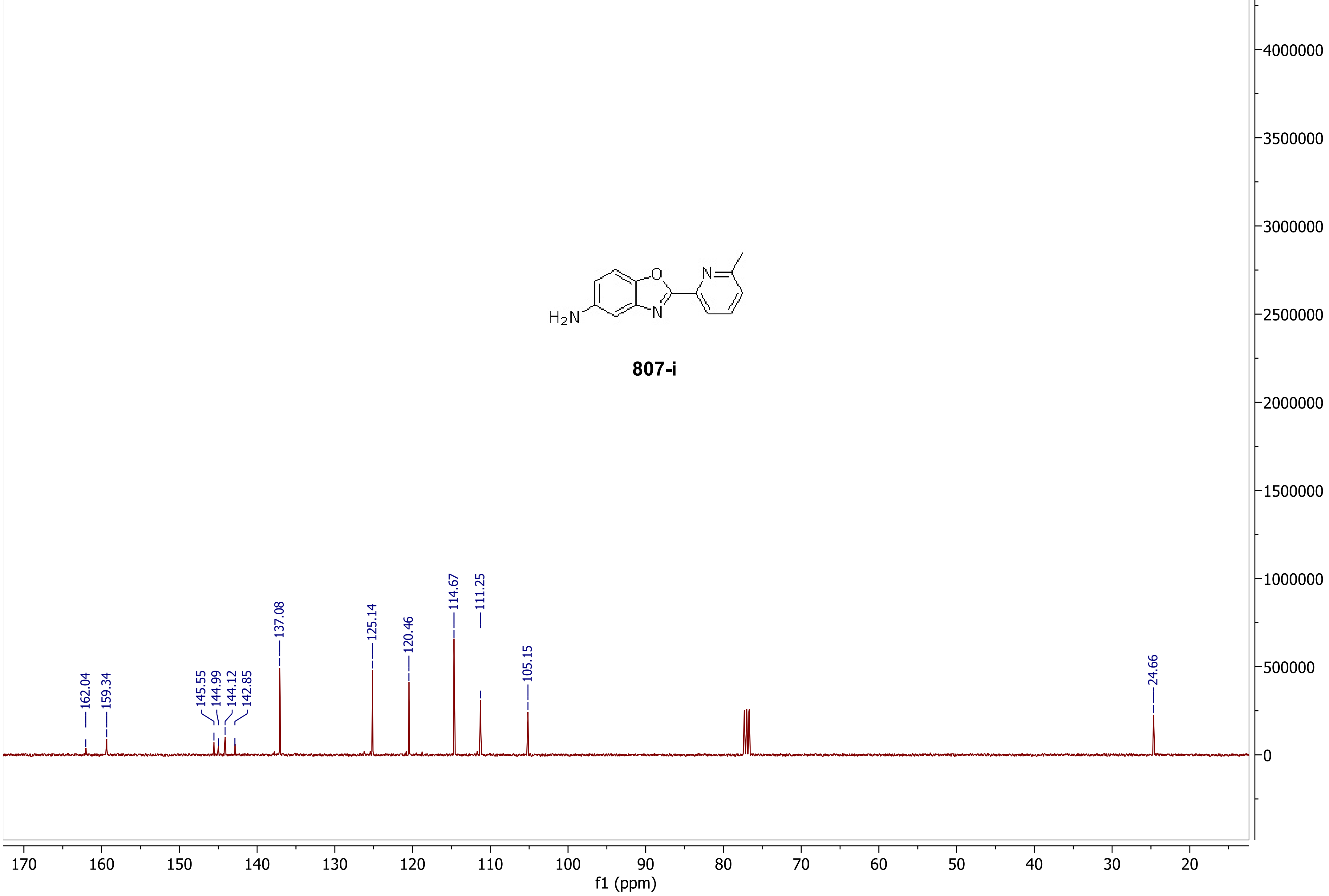

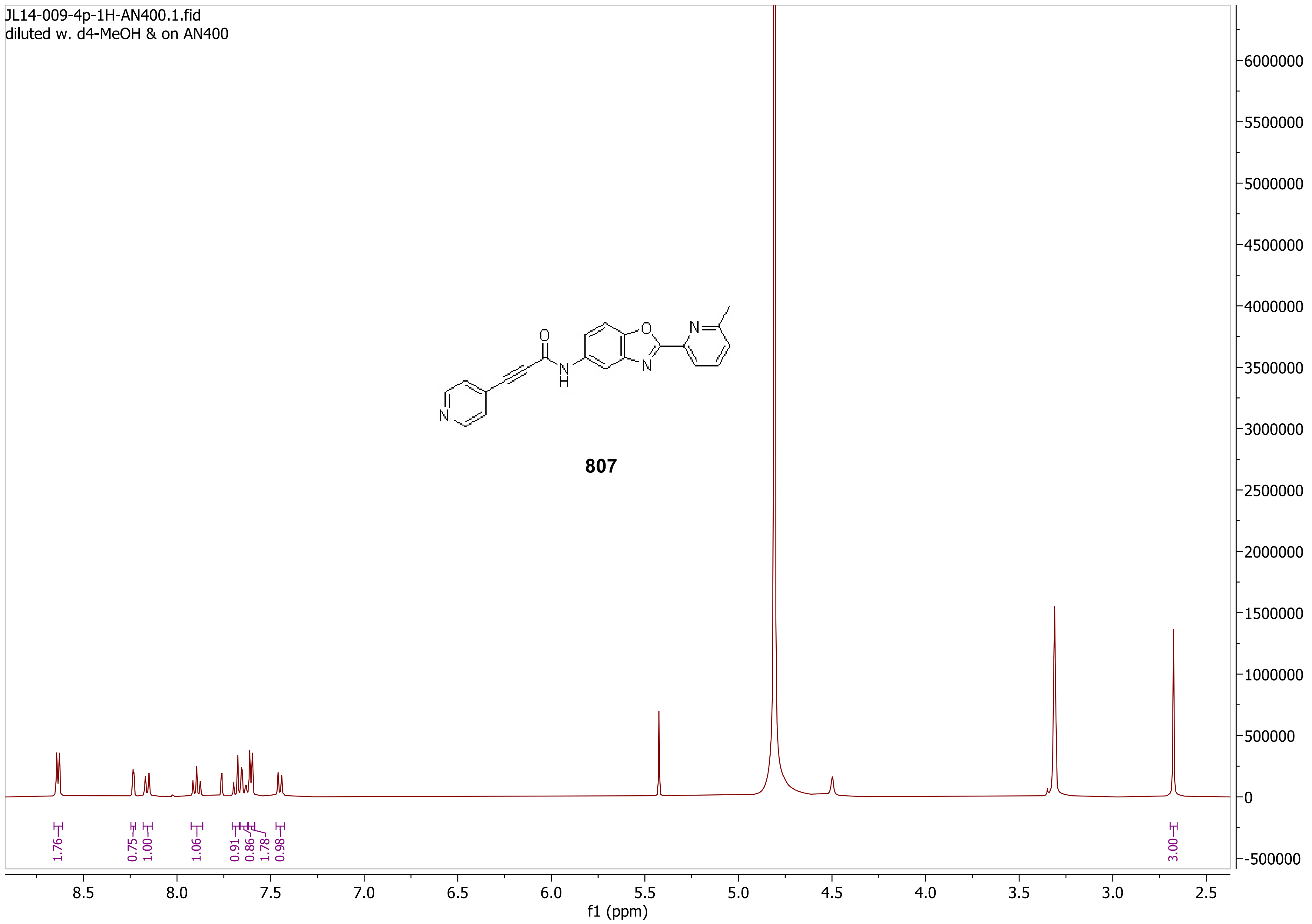
JL14-009-4p-1H-AN400.1.fid
diluted w. d4-MeOH & on AN400
O
N
H
O
N
N
N
807
1.76
0.75
1.00
1.06
0.91
0.86
1.78
0.98
3.00
8.5
8.0
7.5
7.0
6.5
6.0
5.5
5.0
4.5
4.0
3.5
3.0
2.5
f1 (ppm)
6000000
5500000
5000000
4500000
4000000
3500000
3000000
2500000
2000000
1500000
1000000
500000
0
-500000

Notebook 14/JL14-009-4p-13C-AN600-t2
light-yellow solid after 5% MeOH-DCM plug & DCM-hex trituration, tube 2, 13C in d4-MeOH w. 15D CDCl3 on AN600

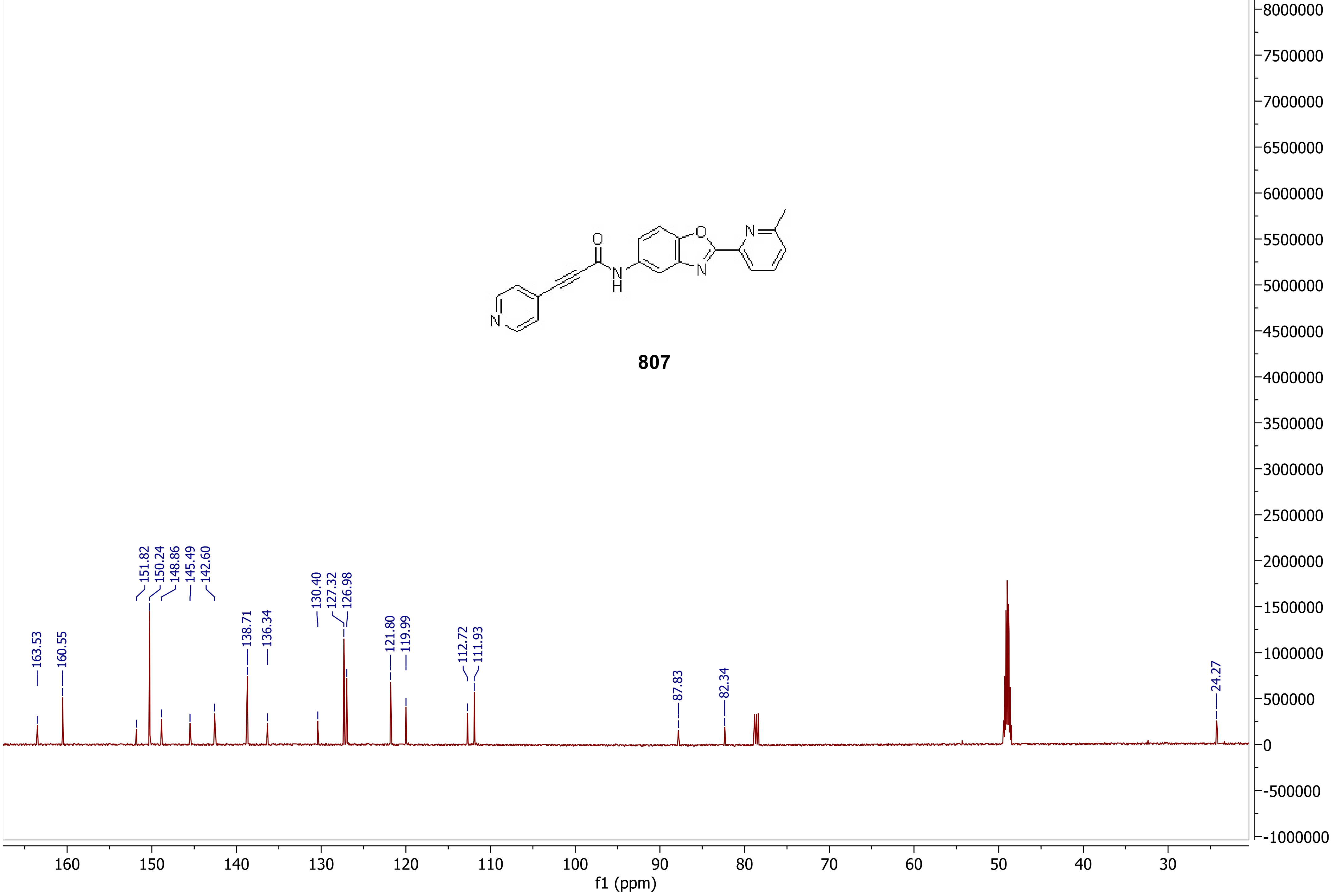

Notebook 14/JL14-008-5p-cr-1H-AN600
yellow solid after DCM extraction and triuration in DCM-hex, supernatant, 1H in CDCl3 on AN600

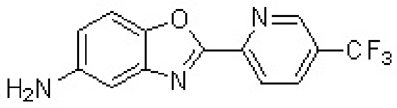

808-i

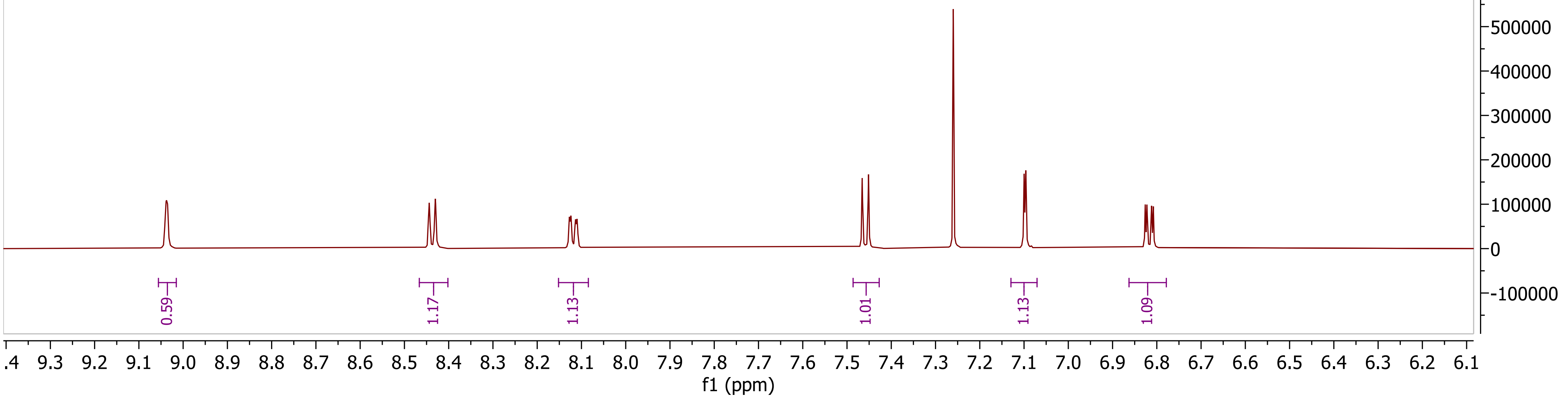

Notebook 14/JL14-008-5p-cr-13C-AN600
yellow solid after DCM extraction and triuration in DCM-hex, supernatant, 13C in CDCl3 on AN600

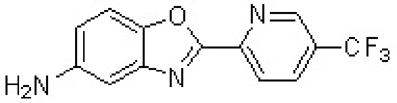

808-i

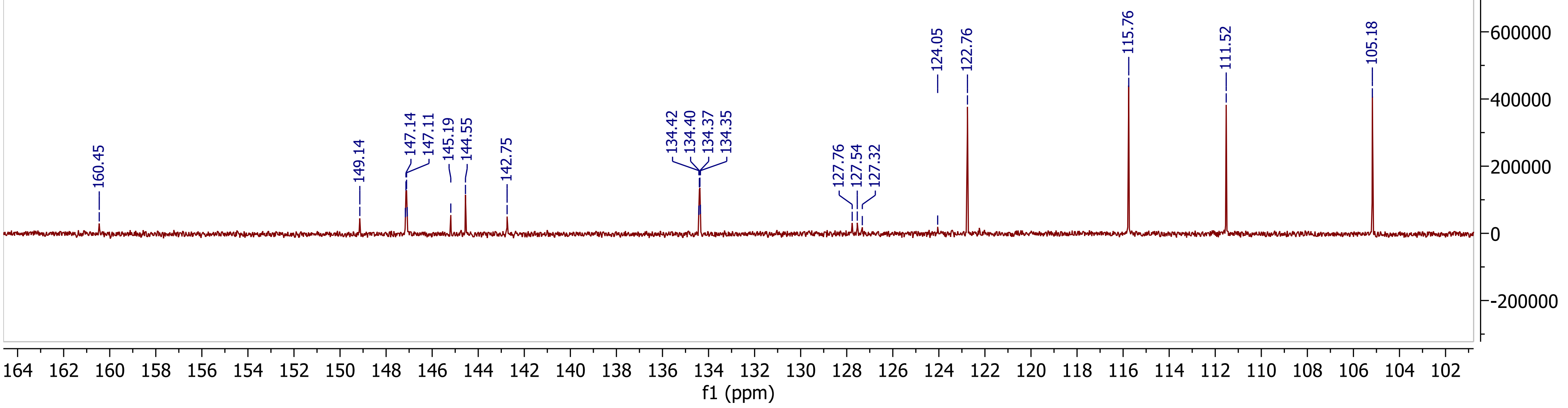

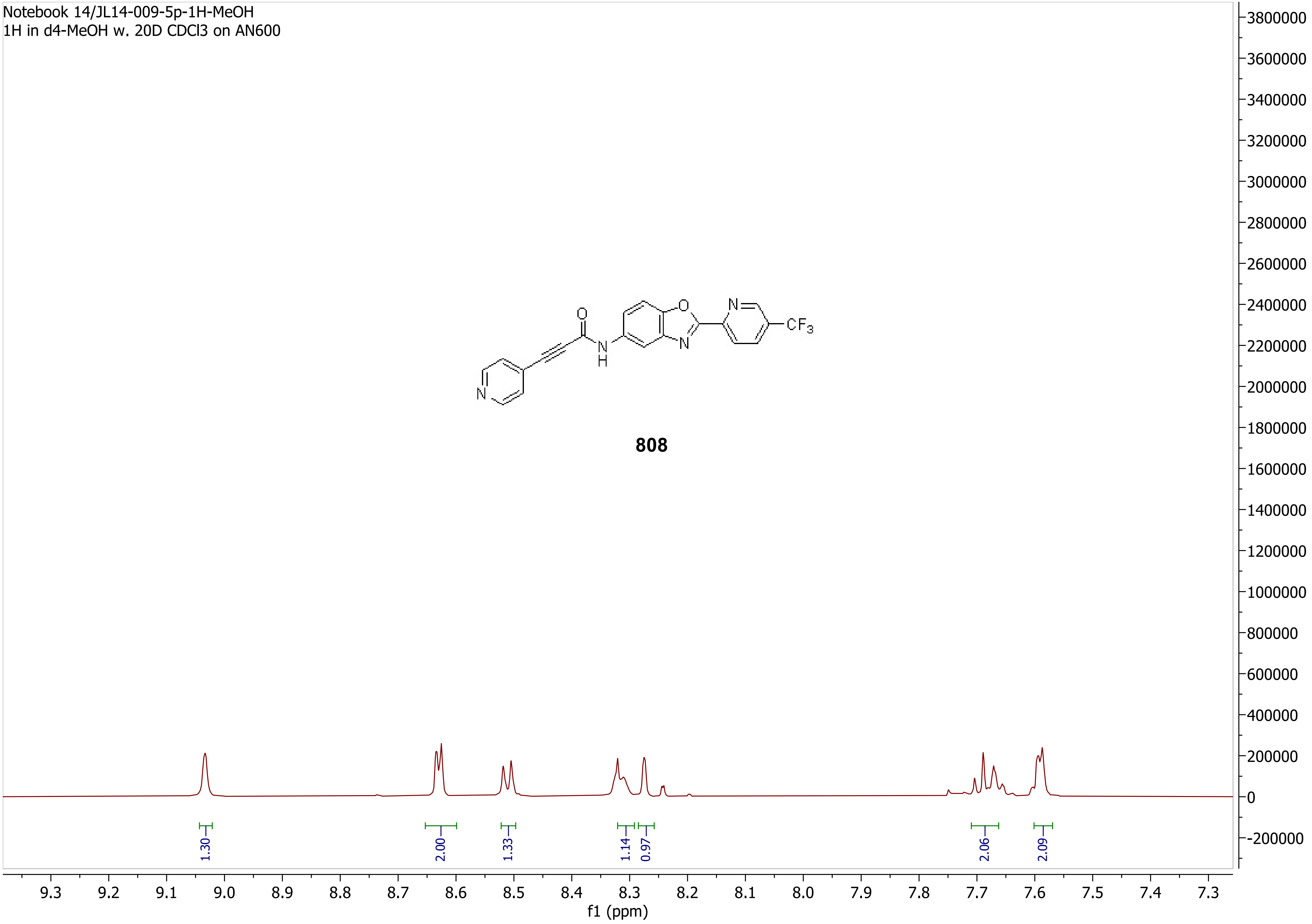

Notebook 14/JL14-009-5p-1H-MeOH
1H in d4-MeOH w. 20D CDCl3 on AN600
O
N
H
O
N
N
CF3
N
808
1.30
2.00
1.33
1.14
0.97
2.06
2.09
f1 (ppm)

Notebook 14/JL14-009-5p-13C-MeOH
13C in d4-MeOH w. 20D CDCl3 on AN600

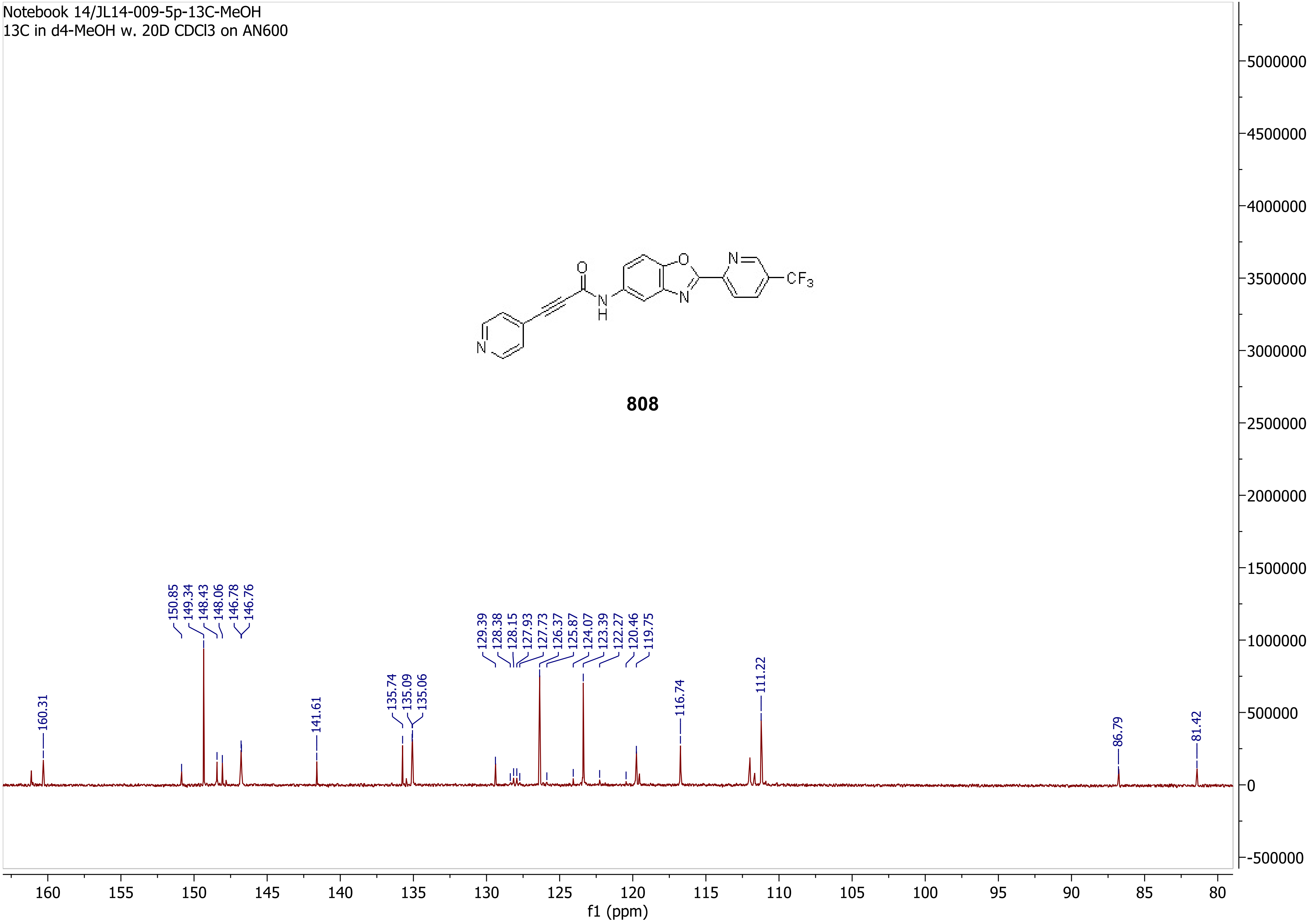

808
